# Diversity and Distribution of *Calonectria* Species from Plantation and Forest Soils in Fujian Province, China

**DOI:** 10.3390/jof8080811

**Published:** 2022-07-31

**Authors:** Qianli Liu, Michael J. Wingfield, Tuan A. Duong, Brenda D. Wingfield, Shuaifei Chen

**Affiliations:** 1 Department of Biochemistry, Genetics and Microbiology, Forestry and Agricultural Biotechnology Institute (FABI), University of Pretoria, Pretoria 0028, South Africa; qianli.liu@fabi.up.ac.za (Q.L.); mike.wingfield@fabi.up.ac.za (M.J.W.); tuan.duong@fabi.up.ac.za (T.A.D.); brenda.wingfield@fabi.up.ac.za (B.D.W.); 2 Research Institute of Fast-Growing Trees (RIFT), Chinese Academy of Forestry (CAF), Zhanjiang 524022, China

**Keywords:** Calonectria leaf blight, forest pathogens, fungal diversity, phylogeny, taxonomy

## Abstract

To meet the growing demand for wood and pulp products, *Eucalyptus* plantations have expanded rapidly during the past two decades, becoming an integral part of the southern China landscape. Leaf blight caused by various *Calonectria* spp., is a serious threat to these plantations. In order to explore the diversity and distribution of *Calonectria* spp. in Fujian Province soils, samples were collected in *Eucalyptus* plantations and adjacent plantings of *Cunninghamia lanceolata*, *Phyllostachys heterocycle* and *Pinus massoniana* as well as in natural forests. Three hundred and fifty-three *Calonectria* isolates were recovered from soil samples and they were identified based on a comparison of multilocus DNA sequence data for the *act* (actin), *cmdA* (calmodulin), *his3* (histone H3), *rpb2* (the second largest subunit of RNA polymerase), *tef1* (translation elongation factor 1-alpha) and *tub2* (β-tubulin) gene regions, as well as morphological characteristics. Six known taxa including *Calonectria aconidialis*, *Ca.* *hongkongensis*, *Ca.* *ilicicola*, *Ca.* *kyotensis*, *Ca.* *pacifica*, *Ca.* *pseudoreteaudii* and one novel species described here as *Ca*. *minensis* sp. nov. were identified. Of these, *Ca*. *aconidialis* and *Ca*. *kyotensis* were the most prevalent species, and found in eight and seven sites, and four and five forest types, respectively. *Calonectria* spp. were most abundant in soils from *Eucalyptus* stands, followed by *P. heterocycle* and natural forests. Relatively few species were found in the soils associated with *Cunninghamia lanceolata* and *Pinus massoniana*. The abundance of known *Calonectria* spp. suggests that these fungi have been relatively well sampled in Fujian. The results are also consistent with the fact that most Calonectria diseases are found on Angiosperm as opposed to Gymnosperm plants.

## 1. Introduction

Species of *Eucalyptus* are the most important trees used to establish plantations in the tropics and Southern Hemisphere, where they provide substantial resources for the global fibre market [1]. These trees were first introduced into China as ornamentals in 1890 and plantations of *Eucalyptus* spp. had reached 5.46 million hm^2^ by 2018 [1]. Plantations of these trees are mainly distributed in 11 provinces of China, and over 75% can be found in the Guangxi, Guangdong, Yunnan and Fujian Provinces of southern China [1]. The *Eucalyptus* plantations in China have been established with a relatively narrow genetic base and consequently many disease problems, caused by a variety of pathogens, have emerged as threats to their sustainability [2,3,4,5,6].

Among the diseases threatening *Eucalyptus* plantations, leaf blight caused by species of *Calonectria* De Not. has become a major constraint in southern China [4,7,8,9,10]. Symptoms of infection are characterised by water-soaked spots on leaves in the lower and middle parts of the tree crowns. These coalesce and gradually develop into extended necrotic areas, which result in blight and often severe defoliation [9]. In China, Calonectria Leaf Blight (CLB) has been observed in *Eucalyptus* plantations in Fujian, Guangdong, Guangxi, Hainan and Yunnan Provinces [4,7,9,10,11]. This is similar to the situation in Australia, Brazil, Indonesia, Thailand and Vietnam where *Eucalyptus* plantations have also suffered significant damage due to CLB [12,13,14,15,16].

The genus *Calonectria* includes many aggressive plant pathogens. These species are extensively distributed particularly in sub-tropical and tropical regions of the world, and they have a wide host range including more than 335 plant species [17]. *Calonectria* species are generally considered as soil-borne fungi and they can survive in the soil for extended periods due to their thick-walled microsclerotia [17].

A recent taxonomic revision of *Calonectria* by Liu and co-authors [18] accepted 120 species. Of these, 65 have been reported from soils samples; the remaining species are known from infections on plant tissues [10,18,19,20,21,22]. To date, 27 species of *Calonectria* have been recorded in China, 18 of which have been isolated from soil samples [4,7,10,11,18,21,23,24,25,26].

**Figure 1:**
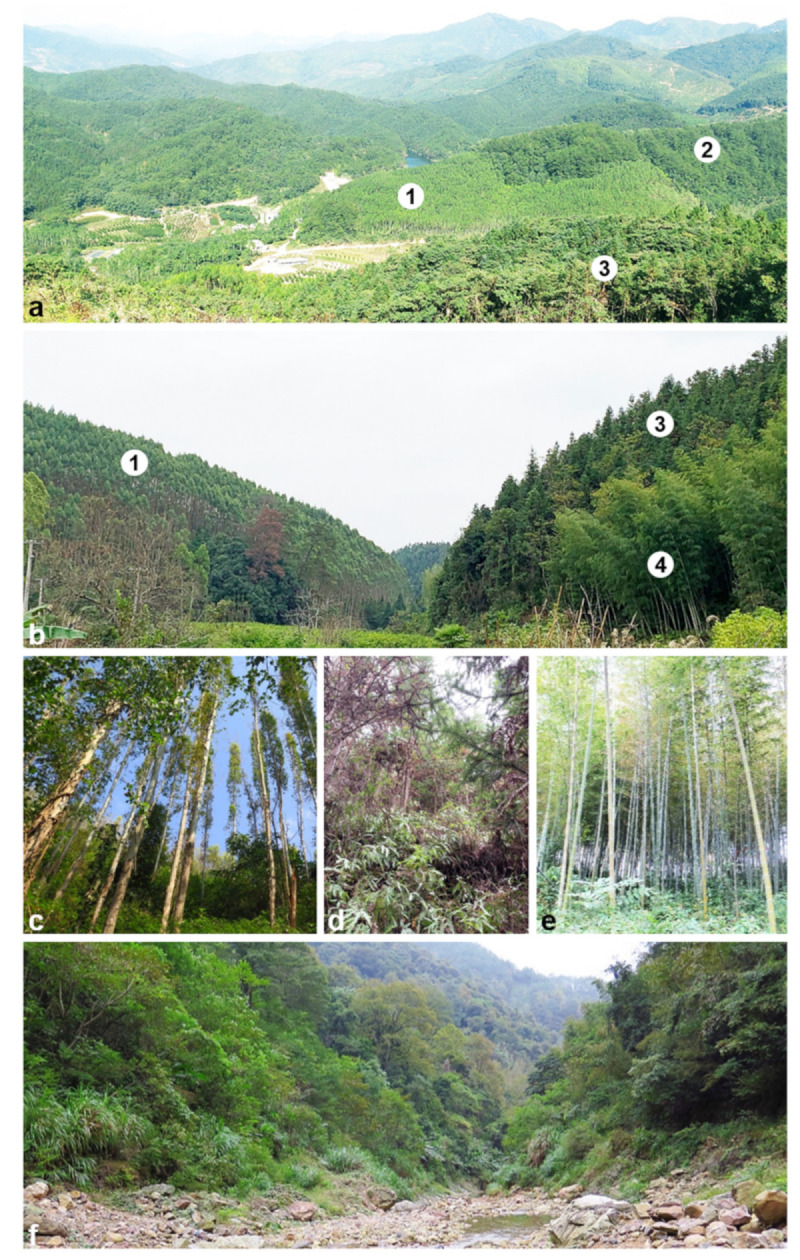
Different forest plantations and natural forests in southern China. (**a**). mixed species plantations in Zhangzhou Region, Fujian Province, 1: *Eucalyptus* sp., 2: *Pinus massoniana*, 3: *Cunninghamia lanceolata*; (**b**). mixed species plantations in Jiangxi Province, 1: *Eucalyptus* sp., 3: *Cunninghamia lanceolata*; 4: *Phyllostachys heterocycle*; (**c**). *Eucalyptus* sp. in Yongan Region, Fujian Province; (**d**). *Cunninghamia lanceolata* in JiangXi Province; (**e**). *Phyllostachys heterocycle* in Nanping Region, Fujian Province; (**f**). natural forests in Nanping Region, Fujian Province. Soil samples in this study were collected from Fujian Province.

Plantations of *Eucalyptus* spp. are commonly established alongside those of *Cunninghamia lanceolata*, *Phyllostachys heterocycle* and *Pinus massoniana* and can also be in mixed plantings in the Fujian Province (Figure 1). In recent years, leaf blight has become a serious threat to *Eucalyptus* plantations in this province [7,8]. *Calonectria* species including *Ca. crousiana*, *Ca*. *eucalypti*, *Ca*. *fujianensis*, *Ca*. *pauciromosa* and *Ca*. *pseudoreteaudii* [7,8,18] have been isolated from diseased *Eucalyptus* tissues and are regarded as the important causal agents of CLB in Fujian. *Calonectria* infections initially arise from inoculum in the soil but very little is known regarding the species diversity and distribution of these fungi in Fujian soils. The aim of this study was thus to determine the identity and distribution of *Calonectria* spp. from a wide variety of soils in Fujian, with a particular focus on *Eucalyptus* spp. but also including other trees that are found in the area.

## 2. Materials and Methods

### 2.1. Sample Collection and Fungal Isolation

**Figure 2:**
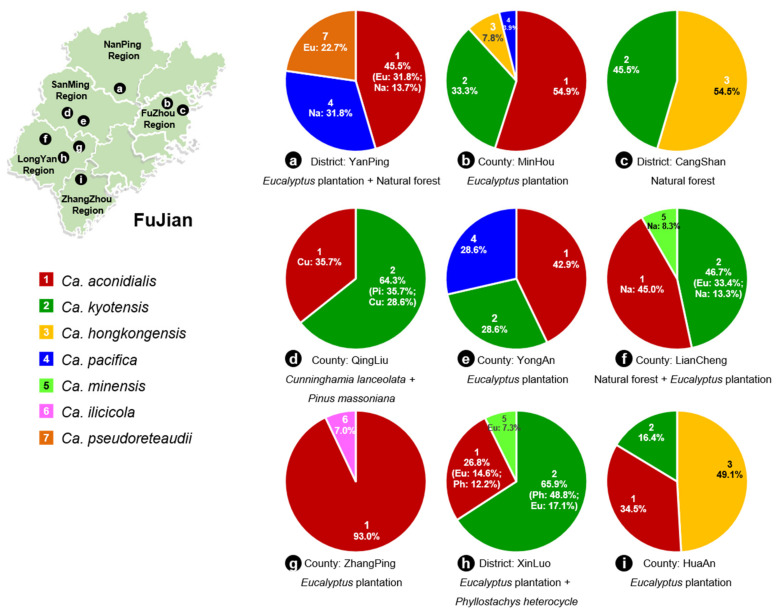
*Calonectria* species collected from nine counties (districts) in Fujian Province. (**a**–**i**). the percentage of each species in nine different counties (districts). Different species are indicated by numbers with different colours.

Soil samples were collected from *Eucalyptus* plantations and adjacent plantings, including those of *Cunninghamia lanceolata*, *Phyllostachys heterocycle* and *Pinus massoniana* as well as in natural forests (Figure 1). These plantations and forests were distributed in nine counties or districts in five regions of Fujian Province (one site in Nanping Region, two sites in Fuzhou Region, two sites in Sanming Region, three sites in Longyan Region, one site in Zhangzhou Region) of southern China (Figure 2). These forests typically have thick layers of leaf litter, which was removed before collecting soil samples from the upper 0–20 cm of the humid soil profile. Between three and 37 soil samples (Table 1) were collected randomly at each site. The soil samples were placed in re-sealable plastic bags to maintain moisture and transported to the laboratory for further study.

Soil samples were placed in plastic cups and moistened using distilled water. *Medicago sativa* (alfalfa) seeds were surface-disinfested in 75% ethanol for 30 s and scattered onto the surface of the moistened soil to bait for *Calonectria* spp. as described by Crous [17]. After eight to ten days at 25 °C, conidiophores typical of *Calonectria* spp. were observed with a Zeiss Stemi 2000C dissection microscope on the germinating alfalfa plants. Conidial masses were transferred to 2% MEA (Malt Extract Agar) using a sterile needle. After 12 h of incubation at 25 °C, single hyphal tips were transferred to fresh MEA plates using a sterile needle and these cultures were incubated at 25 °C for seven days. Cultures were sorted based on their morphological characteristics and one to five isolates were retained for each of the soil samples.

Cultures were deposited in the Culture Collection (CSF) at the Research Institute of Fast-growing Trees (RIFT) (previous institution: China Eucalypt Research Centre, CERC), Chinese Academy of Forestry (CAF), ZhanJiang, Guangdong Province, China. Representative isolates have also been maintained in the China General Microbiological Culture Collection Centre (CGMCC), Beijing, China. Dried specimens were deposited in the Mycological Fungarium of the Institute of Microbiology, Chinese Academy of Sciences (HMAS), Beijing, China.

### 2.2. DNA Extraction, PCR Amplifications and Sequencing

Mycelium was collected from axenic cultures grown on MEA for 5–7 days using a sterilised scalpel. Genomic DNA was extracted from the cultures using the CTAB method “5” described by Van Burik et al. [27]. Partial gene sequences were determined for the actin (*act*), calmodulin (*cmdA*), histone H3 (*his3*), the second largest subunit of RNA polymerase (*rpb2*), translation elongation factor 1-alpha (*tef1*) and β-tubulin (*tub2*) regions. Primer pairs ACT-512F/ACT-783R, CAL-228F/CAL-2Rd, CYLH3F/CYLH3R, fRpb2-5F/fRpb2-7cR, EF1-728F/EF2 and T1/CYLTUB1R [18] were used to amplify the six gene regions, respectively.

The PCR reaction mixtures contained 17.5 μL TopTaq^TM^ Master Mix, 1 μL of each primer (10 mM), 2 μL DNA sample and RNase-Free H_2_O to a final volume of 35 μL. The amplifications were conducted under conditions described by Liu and co-authors [18]. All PCR products were sequenced in both directions using the same primers used for amplification. Raw sequences were inspected and manually corrected in Geneious v. 9.1.4 (Biomatters, Auckland, New Zealand) [28]. All sequences generated in this study were submitted to GenBank (http://www.ncbi.nlm.nih.gov; accessed on 24 July 2022) (Table 2, Appendix A Table A1).

### 2.3. Phylogenetic Analyses

To obtain the preliminary identification of the isolates, a standard nucleotide BLAST search was conducted using sequences of the six (*act*, *cmdA*, *his3*, *rpb2*, *tef1* and *tub2*) gene regions. Furthermore, sequences obtained in this study (Table 2) and sequences of other phylogenetically closely related *Calonectria* species downloaded from NCBI (http://www.ncbi.nlm.nih.gov; accessed on 24 July 2022) (Table 3) were used in the analyses. Sequence alignments were conducted online with MAFFT v. 7 (Suita, Janpan) [29] and were manually adjusted in MEGA v. 6.0.5 software (Auckland, New Zealand) [30] when necessary. The final alignments used in phylogenetic analyses were submitted to TreeBASE (http://treebase.org; accessed on 3 October 2021).

Genotypes of all the isolates were determined based on the sequences for the six gene regions. Representative isolates for all the genotypes were selected for the phylogenetic analyses. All the isolates of the novel species were used in the analyses. Maximum Parsimony (MP) and Maximum Likelihood (ML) approaches were used for phylogenetic analyses. The sequence datasets for the six individual gene regions and a concatenated dataset for those regions were used to determine the phylogenetic relatedness of all the isolates. PAUP v. 4.0 b10 [31] was used to perform the MP analyses, and PhyML v. 3.0 [32] was applied to conduct the ML analyses. A partition homogeneity test (PHT) [33] was performed to assess whether the datasets for the six gene regions could be combined.

For MP analyses, all characters were unordered and equally weighted. Gaps were regarded as fifth character and phylogenetic trees were obtained using a heuristic tree search criterion including 1000 random stepwise additions and tree-bisection-reconstruction (TBR) branch swapping. Branches of zero-length were collapsed. Supports for tree-branching points were determined using bootstrap analyses with 1000 replicates [34]. Tree length (TL), retention index (RI), consistency index (CI), rescaled consistency indexes (RC) and homoplasy index (HI) (Table 4) were calculated for parsimony trees. For ML analyses, the best substitution model for each dataset was determined using JModeltest 2.1.7 [35]. Sequence data for two isolates of *Curvicladiella cignea* (CBS 109167 and CBS 109168) were used as outgroup taxa (Table 3).

**Table 3:** Isolates from other studies and used in the phylogenetic analyses.

Code B ^a^	Species	Isolates No. ^b,c^	Other Collection Number ^b^	Substrate	Area of Occurrence	Collector	GenBank accession No. ^d^	References
							*act*; *cmdA*; *his3*; *rpb2*; *tef1*; *tub2*	
B1	*Calonectria* *acaciicola*	CMW 47173^T^	CBS 143557	Soil (*Acacia auriculiformis* plantation)	Do Luong, Nghe An, Vietnam	N.Q. Pham and T.Q. Pham	MT334933; MT335160; MT335399; MT412474; MT412690; MT412930	[16,18]
		CMW 47174	CBS 143558	Soil (*A. auriculiformis* plantation)	Do Luong, Nghe An, Vietnam	N.Q. Pham and T.Q. Pham	MT334934; MT335161; MT335400; MT412475; MT412691; MT412931	[16,18]
B2	*Ca. acicola*	CMW 30996^T^	–	*Phoenix canariensis*	Northland, New Zealand	H. Pearson	MT334935; MT335162; MT335401; MT412476; MT412692; MT412932	[18,36,37]
		CBS 114812	CMW 51216	*P. canariensis*	Northland, New Zealand	H. Pearson	MT334936; MT335163; MT335402; MT412477; MT412693; MT412933	[18,36,37]
B3	*Ca. aciculata*	CERC 5342^T^	CBS 142883; CMW 47645	*Eucalyptus urophylla* × *E. grandis*	Yunnan, China	S.F. Chen and J.Q. Li	MT334937; MT335164; MT335403; MT412478; MT412694; MT412934	[4,18]
B4	*Ca. aconidialis*	CMW 35174^T^	CBS 136086; CERC 1850	Soil (*Eucalyptus* plantation)	Hainan, China	X. Mou and S.F. Chen	MT334938; MT335165; MT335404; MT412479; MT412695; N/A ^e^	[11,18]
		CMW 35384	CBS 136091; CERC 1886	Soil (*Eucalyptus* plantation)	Hainan, China	X. Mou and S.F. Chen	MT334939; MT335166; MT335405; N/A; MT412696; N/A	[11,18]
B5	*Ca. aeknauliensis*	CMW 48253^T^	CBS 143559	Soil (*Eucalyptus* plantation)	Aek Nauli, North Sumatra, Indonesia	M.J. Wingfield	MT334953; MT335180; MT335419; MT412486; MT412710; N/A	[16,18]
		CMW 48254	CBS 143560	Soil (*Eucalyptus* plantation)	Aek Nauli, North Sumatra, Indonesia	M.J. Wingfield	MT334954; MT335181; MT335420; MT412487; MT412711; N/A	[16,18]
B8	*Ca. asiatica*	CBS 114073^T^	CMW 23782; CPC 3900	Debris (leaf litter)	Prathet Thai, Thailand	N.L. Hywel-Jones	GQ280428; AY725741; AY725658; N/A; AY725705; AY725616	[23,37]
B10	*Ca. australiensis*	CMW 23669^T^	CBS 112954; CPC 4714	*Ficus pleurocarpa*	Queensland, Australia	C. Pearce and B. Paulus	MT334965; MT335192; MT335432; MT412496; MT412723; MT412946	[18,37,38]
B17	*Ca. brassicicola*	CBS 112841^T^	CMW 51206; CPC 4552	Soil at *Brassica* sp.	Indonesia	M.J. Wingfield	N/A; KX784561; N/A; N/A; KX784689; KX784619	[39]
B19	*Ca. bumicola*	CMW 48257^T^	CBS 143575	Soil (*Eucalyptus* plantation)	Aek Nauli, North Sumatra, Indonesia	M.J. Wingfield	MT334975; MT335205; MT335445; MT412509; MT412736; N/A	[16,18]
B20	*Ca. canadiana*	CMW 23673^T^	CBS 110817; STE-U 499	*Picea* sp.	Canada	S. Greifenhagen	MT334976; MT335206; MT335446; MT412510; MT412737; MT412958	[17,18,40,41]
		CERC 8952	–	Soil	Henan, China	S.F. Chen	MT335058; MT335290; MT335530; MT412587; MT412821; MT413035	[18,25]
B23	*Ca. chinensis*	CMW 23674^T^	CBS 114827; CPC 4101	Soil	Hong Kong, China	E.C.Y. Liew	MT334990; MT335220; MT335460; MT412524; MT412751; MT412972	[18,23,37]
		CMW 30986	CBS 112744; CPC 4104	Soil	Hong Kong, China	E.C.Y. Liew	MT334991; MT335221; MT335461; MT412525; MT412752; MT412973	[18,23,37]
B26	*Ca. cochinchinensis*	CMW 49915^T^	CBS 143567	Soil (*Hevea brasiliensis* plantation)	Duong Minh Chau, Tay Ninh, Vietnam	N.Q. Pham, Q.N. Dang and T.Q. Pham	MT334995; MT335225; MT335465; MT412529; MT412756; MT412977	[16,18]
		CMW 47186	CBS 143568	Soil (*A. auriculiformis* plantation)	Song May, Dong Nai, Vietnam	N.Q. Pham and T.Q. Pham	MT334996; MT335226; MT335466; MT412530; MT412757; MT412978	[16,18]
B27	*Ca. colhounii*	CBS 293.79^T^	CMW 30999	*Camellia sinensis*	Mauritius	A. Peerally	GQ280443; GQ267373; DQ190639; KY653376; GQ267301; DQ190564	[17,37,38,42]
B29	*Ca. colombiensis*	CMW 23676^T^	CBS 112220; CPC 723	Soil (*E. grandis* trees)	La Selva, Colombia	M.J. Wingfield	MT334998; MT335228; MT335468; MT412532; MT412759; MT412980	[18,23]
		CMW 30985	CBS 112221; CPC 724	Soil (*E. grandis* trees)	La Selva, Colombia	M.J. Wingfield	MT334999; MT335229; MT335469; MT412533; MT412760; MT412981	[18,23]
B30	*Ca. crousiana*	CMW 27249^T^	CBS 127198	*E. grandis*	Fujian, China	M.J. Wingfield	MT335000; MT335230; MT335470; MT412534; MT412761; MT412982	[7,18]
		CMW 27253	CBS 127199	*E. grandis*	Fujian, China	M.J. Wingfield	MT335001; MT335231; MT335471; MT412535; MT412762; MT412983	[7,18]
B31	*Ca. curvispora*	CMW 23693^T^	CBS 116159; CPC 765	Soil	Tamatave, Madagascar	P.W. Crous	MT335002; MT335232; MT335472; MT412536; MT412763; N/A	[11,17,18,37,43]
		CMW 48245	CBS 143565	Soil (*Eucalyptus* plantation)	Aek Nauli, North Sumatra, Indonesia	M.J. Wingfield	MT335003; MT335233; MT335473; MT412537; MT412764; N/A	[16,18]
B36	*Ca. eucalypti*	CMW 18444^T^	CBS 125275	*E. grandis*	Aek Nauli, Sumatra Utara, Indonesia	M.J. Wingfield	MT335013; MT335243; MT335483; MT412545; MT412774; MT412992	[18,37]
		CMW 18445	CBS 125276	*E. grandis*	Aek Nauli, Sumatra Utara, Indonesia	M.J. Wingfield	MT335014; MT335244; MT335484; MT412546; MT412775; MT412993	[18,37]
B39	*Ca. fujianensis*	CMW 27257^T^	CBS 127201	*E. grandis*	Fujian, China	M.J. Wingfield	MT335019; MT335249; MT335489; MT412551; MT412780; MT412998	[7,18]
		CMW 27254	CBS 127200	*E. grandis*	Fujian, China	M.J. Wingfield	MT335020; MT335250; MT335490; MT412552; MT412781; MT412999	[7,18]
B46	*Ca. heveicola*	CMW 49913^T^	CBS 143570	Soil (*H. brasiliensis* plantation)	Bau Bang, Binh Duong, Vietnam	N.Q. Pham, Q.N. Dang and T.Q. Pham	MT335025; MT335255; MT335495; N/A; MT412786; MT413004	[16,18]
		CMW 49928	CBS 143571	Soil	Bu Gia Map National Park, Binh Phuoc, Vietnam	N.Q. Pham, Q.N. Dang and T.Q. Pham	MT335048; MT335280; MT335520; MT412577; MT412811; MT413025	[16,18]
B47	*Ca. honghensis*	CERC 5572^T^	CBS 142885; CMW 47669	Soil (*Eucalyptus* plantation)	Honghe, Yunnan, China	S.F. Chen and J.Q. Li	MT335026; MT335256; MT335496; MT412557; MT412787; MT413005	[4,18]
		CERC 5571	CBS 142884; CMW 47668	Soil (*Eucalyptus* plantation)	Honghe, Yunnan, China	S.F. Chen and J.Q. Li	MT335027; MT335257; MT335497; MT412558; MT412788; MT413006	[4,18]
B48	*Ca. hongkongensis*	CBS 114828^T^	CMW 51217; CPC 4670	Soil	Hong Kong, China	M.J. Wingfield	MT335028; MT335258; MT335498; MT412559; MT412789; MT413007	[18,23]
		CERC 3570	CMW 47271	Soil (*Eucalyptus* plantation)	Beihai, Guangxi, China	S.F. Chen, J.Q. Li and G.Q. Li	MT335030; MT335260; MT335500; MT412561; MT412791; MT413009	[4,18]
B51	*Ca. ilicicola*	CMW 30998^T^	CBS 190.50; IMI 299389; STE-U 2482	*Solanum tuberosum*	Bogor, Java, Indonesia	K.B. Boedijn and J. Reitsma	MT335036; MT335266; MT335506; MT412564; MT412797; N/A	[17,18,37,44]
B52	*Ca. indonesiae*	CMW 23683^T^	CBS 112823; CPC 4508	*Syzygium* *aromaticum*	Warambunga, Indonesia	M.J. Wingfield	MT335037; MT335267; MT335507; MT412565; MT412798; MT413015	[18,23]
		CBS 112840	CMW 51205; CPC 4554	*S. aromaticum*	Warambunga, Indonesia	M.J. Wingfield	MT335038; MT335268; MT335508; MT412566; MT412799; MT413016	[18,23]
B53	*Ca. indusiata*	CBS 144.36^T^	CMW 23699	*Camellia sinensis*	Sri lanka	N/A	GQ280536; GQ267453; GQ267262; KY653396; GQ267332; GQ267239	[17,37,39,45]
		CBS 114684	CMW 51213; CPC 2446; UFV16	*Rhododendron* sp.	Florida, USA	N.E. El-Gholl	GQ280537; GQ267454; DQ190653; N/A; GQ267333; AF232862	[17,38,46]
B55	*Ca. kyotensis*	CBS 114525^T^	ATCC 18834; CMW 51824; CPC 2367	*Robinia* *pseudoacacia*	Japan	T. Terashita	MT335039; MT335271; MT335511; MT412569; MT412802; MT413019	[17,18,39,47]
		CBS 114550	CMW 51825; CPC 2351	Soil	China	M.J. Wingfield	MT335016; MT335246; MT335486; MT412548; MT412777; MT412995	[18,39]
B57	*Ca. lantauensis*	CERC 3302^T^	CBS 142888; CMW 47252	Soil	LiDao, Hong Kong, China	M.J. Wingfield and S.F. Chen	MT335040; MT335272; MT335512; MT412570; MT412803; N/A	[4,18]
		CERC 3301	CBS 142887; CMW 47251	Soil	LiDao, Hong Kong, China	M.J. Wingfield and S.F. Chen	MT335041; MT335273; MT335513; N/A; MT412804; N/A	[4,18]
B58	*Ca. lateralis*	CMW 31412^T^	CBS 136629	Soil (*Eucalyptus* plantation)	Guangxi, China	X. Zhou, G. Zhao and F. Han	MT335042; MT335274; MT335514; MT412571; MT412805; MT413020	[11,18]
B62	*Ca. lichi*	CERC 8866^T^	–	Soil	Henan, China	S.F. Chen	MT335046; MT335278; MT335518; MT412575; MT412809; MT413023	[18,25]
		CERC 8850	–	Soil	Henan, China	S.F. Chen	MT335047; MT335279; MT335519; MT412576; MT412810; MT413024	[18,25]
B63	*Ca. lombardiana*	CMW 30602^T^	CBS 112634; CPC 4233; Lynfield 417	*Xanthorrhoea australis*	Victoria, Australia	T. Baigent	MT335156; MT335395; MT335635; MT412686; MT412926; MT413133	[17,18,24,38]
B64	*Ca. macroconidialis*	CBS 114880^T^	CMW 51219; CPC 307; PPRI 4000	*E. grandis*	Sabie, Mpumalanga, South Africa	P.W. Crous	MT335050; MT335282; MT335522; MT412579; MT412813; MT413027	[17,18,37,48]
B65	*Ca. madagascariensis*	CMW 23686^T^	CBS 114572; CPC 2252	Soil	Rona, Madagascar	J.E. Taylor	MT335052; MT335284; MT335524; MT412581; MT412815; MT413029	[17,18,37,38]
		CMW 30993	CBS 114571; CPC 2253	Soil	Rona, Madagascar	J.E. Taylor	MT335053; MT335285; MT335525; MT412582; MT412816; MT413030	[17,18,37,38]
B66	*Ca. malesiana*	CMW 23687^T^	CBS 112752; CPC 4223	Soil	Northern Sumatra, Indonesia	M.J. Wingfield	MT335054; MT335286; MT335526; MT412583; MT412817; MT413031	[18,23]
		CBS 112710	CMW 51199; CPC 3899	Leaf litter	Prathet, Thailand	N.L. Hywel-Jones	MT335055; MT335287; MT335527; MT412584; MT412818; MT413032	[18,23]
B70	*Ca. monticola*	CBS 140645^T^	CPC 28835	Soil	Chiang Mai, Thailand	P.W. Crous	N/A; KT964771; N/A; N/A; KT964773; KT964769	[49]
		CPC 28836	–	Soil	Chiang Mai, Thailand	P.W. Crous	N/A; KT964772; N/A; N/A; KT964774; KT964770	[49]
B74	*Ca. multiseptata*	CMW 23692^T^	CBS 112682; CPC 1589	*E. grandis*	North Sumatra, Indonesia	M.J. Wingfield	MT335067; MT335299; MT335539; MT412596; MT412830; MT413044	[17,18,38,50]
B80	*Ca. pacifica*	CMW 16726^T^	A1568; CBS 109063; IMI 354528; STE-U 2534	*Araucaria* *heterophylla*	Hawaii, USA	M. Aragaki	MT335079; MT335311; MT335551; MT412604; MT412842; N/A	[17,18,23,40]
		CMW 30988	CBS 114038	*Ipomoea aquatica*	Auckland, New Zealand	C.F. Hill	MT335080; MT335312; MT335552; MT412605; MT412843; N/A	[17,18,23,37]
B81	*Ca. paracolhounii*	CBS 114679^T^	CMW 51212; CPC 2445	N/A	USA	A.Y. Rossman	N/A; KX784582; N/A; KY653423; KX784714; KX784644	[39,45]
		CBS 114705	CMW 51215; CPC 2423	Fruit of *Annona reticulata*	Australia	D. Hutton	N/A; N/A; N/A; KY653424; KX784715; KX784645	[39,45]
B86	*Ca. penicilloides*	CMW 23696^T^	CBS 174.55; STE-U 2388	*Prunus* sp.	Hatizyo Island, Japan	M. Ookubu	MT335106; MT335338; MT335578; MT412631; MT412869; MT413081	[17,18,51]
B97	*Ca. pseudoreteaudii*	CMW 25310^T^	CBS 123694	*E. urophylla* × *E. grandis*	Guangdong, China	M.J. Wingfield and X.D. Zhou	MT335119; MT335354; MT335594; MT412647; MT412885; MT413096	[18,24]
		CMW 25292	CBS 123696	*E. urophylla* × *E. grandis*	Guangdong, China	M.J. Wingfield and X.D. Zhou	MT335120; MT335355; MT335595; MT412648; MT412886; MT413097	[18,24]
B104	*Ca. queenslandica*	CMW 30604^T^	CBS 112146; CPC 3213	*E. urophylla*	Lannercost, Queensland, Australia	B. Brown	MT335132; MT335367; MT335607; MT412660; MT412898; MT413108	[18,24]
		CMW 30603	CBS 112155; CPC 3210	*E. pellita*	Lannercost, Queensland, Australia	P.Q Thu and K.M. Old	MT335133; MT335368; MT335608; MT412661; MT412899; MT413109	[18,24]
B106	*Ca. reteaudii*	CMW 30984^T^	CBS 112144; CPC 3201	*E. camaldulensis*	Chon Thanh, Binh Phuoc, Vietnam	M.J. Dudzinski and P.Q. Thu	MT335135; MT335370; MT335610; MT412663; MT412901; MT413111	[17,18,38,52]
		CMW 16738	CBS 112143; CPC 3200	*Eucalyptus* leaves	Binh Phuoc, Vietnam	M.J. Dudzinski and P.Q. Thu	MT335136; MT335371; MT335611; MT412664; MT412902; MT413112	[17,18,38,52]
B112	*Ca. sumatrensis*	CMW 23698^T^	CBS 112829; CPC 4518	Soil	Northern Sumatra, Indonesia	M.J. Wingfield	MT335145; MT335382; MT335622; MT412674; MT412913; N/A	[18,23]
		CMW 30987	CBS 112934; CPC 4516	Soil	Northern Sumatra, Indonesia	M.J. Wingfield	MT335146; MT335383; MT335623; MT412675; MT412914; N/A	[18,23]
B113	*Ca. syzygiicola*	CBS 112831^T^	CMW 51204; CPC 4511	*S. aromaticum*	Sumatra, Indonesia	M.J. Wingfield	N/A; N/A; N/A; N/A; KX784736; KX784663	[39]
B116	*Ca. uniseptata*	CBS 413.67^T^	CMW 23678; CPC 2391; IMI 299577	*Paphiopedilum callosum*	Celle, Germany	W. Gerlach	GQ280451; GQ267379; GQ267248; N/A; GQ267307; GQ267208	[39]
B123	*Ca. xianrensis*	CSF12909^T^	CGMCC3.19584	Soil (near *Eucalyptus* plantation)	Dacheng Town, Gaozhou County, Maoming Region, Guangdong, China	S.F. Chen, Q.C. Wang and W. Wang	N/A; MK962845; MK962857; N/A; MK962869; MK962833	[21]
		CSF12908	CGMCC3.19518	Soil (near *Eucalyptus* plantation)	Dacheng Town, Gaozhou County, Maoming Region, Guangdong, China	S.F. Chen, Q.C. Wang and W. Wang	N/A; MK962844; MK962856; N/A; MK962868; MK962832	[21]
B120	*Ca. yunnanensis*	CERC 5339^T^	CBS 142897; CMW 47644	Soil (*Eucalyptus* plantation)	Yunnan, China	S.F. Chen and J.Q. Li	MT335157; MT335396; MT335636; MT412687; MT412927; MT413134	[4,18]
		CERC 5337	CBS 142895; CMW 47642	Soil (*Eucalyptus* plantation)	Yunnan, China	S.F. Chen and J.Q. Li	MT335158; MT335397; MT335637; MT412688; MT412928; MT413135	[4,18]
	*Curvicladiella cignea*	CBS 109167^T^	CPC 1595; MUCL 40269	Decaying leaf	French Guiana	C. Decock	KM231122; KM231287; KM231461; KM232311; KM231867; KM232002	[11,38,53]
		CBS 109168	CPC 1594; MUCL 40268	Decaying seed	French Guiana	C. Decock	KM231121; KM231286; KM231460; KM232312; KM231868; KM232003	[11,38,53]

^a^ Codes (B1 to B120) of the 120 accepted *Calonectria* species resulting from Liu and co-authors [18]. ^b^ *ATCC* = American Type Culture Collection, Virginia, USA; *CBS* = Westerdijk Fungal Biodiversity Institute, Utrecht, The Netherlands; *CERC* = China Eucalypt Research Centre, ZhanJiang, Guangdong Province, China; *CGMCC* = China General Microbiological Culture Collection Center, Beijing, China; *CMW* = Culture collection of the Forestry and Agricultural Biotechnology Institute (FABI), University of Pretoria, Pretoria, South Africa; *CPC* = Pedro Crous working collection housed at Westerdijk Fungal Biodiversity Institute; *CSF* = Culture Collection from Southern Forests (CSF), ZhanJiang, Guangdong Province, China; *IMI* = International Mycological Institute, CABI Bioscience, Egham, Bakeham Lane, UK; *MUCL* = Mycotheque, Laboratoire de Mycologie Systematique st Appliqee, I’Universite, Louvian-la-Neuve, Belgium; *PPRI* = Plant Protection Research Institute, Pretoria, South Africa; *STE-U* = Department of Plant Pathology, University of Stellenbosch, South Africa; ‘–’ represent no other collection number. ^c^ *T* = ex-type isolates of the species. ^d^ *act* = actin; *cmdA* = calmodulin; *his3* = histone H3; *rpb2* = the second largest subunit of RNA polymerase; *tef1* = translation elongation factor 1-alpha; *tub2* = β-tubulin. ^e^ *N/A* represents information not available.

### 2.4. Sexual Compatibility

The mating system as either homothallic or heterothallic was determined for the novel species identified in this study. Representative isolates of this species were crossed with each other in all possible combinations. These crosses were made on minimum salt agar (MSA) [54] with autoclaved toothpicks randomly placed on the agar surface. Petri dishes were then incubated at 25 °C for 2–8 wk, and they were observed regularly for the appearance of perithecia. When perithecia extruding ascospores emerged, germination tests were conducted to determine if the spores were viable. Production of viable ascospores was accepted as an indication of successful mating.

### 2.5. Morphology

Representative isolates of the novel species identified in this study were selected for morphological characterisation. Synthetic nutrient-poor agar (SNA) [55] was used to induce the asexual morphs. Agar plugs from axenic cultures were transferred to SNA and incubated at 25 °C for seven days. Fungal structures were lifted from the plates using a sterile needle and transferred to a drop of 85% lactic acid on microscope slides. Microscopic structures were examined under a Zeiss Axio Imager A1 microscope (Carl Zeiss Ltd., Jena, Germany).

In the case of sexual structures, the perithecia were transferred to Jung tissue freezing medium (Leica Biosystems, Wetzlar, Germany), which was frozen at −20 °C for ten minutes. Vertical sections (10 µm thick) were cut through the perithecia on a HM550 cryostat microtome (Microme International GmbH, Termo Fisher Scientifc, Walldorf, Germany) at −20 °C and examined under an Axio Imager A1 microscope.

For cultures selected as the ex-type isolates, 50 replicate measurements were made for each taxonomically characteristic structure. For other isolates, 30 replicate measurements were made. Minimum, maximum and average (mean) measurements were recorded as (minimum–) (average–standard deviation)–(average + standard deviation) (–maximum).

Optimal growth temperatures for the novel species were determined on MEA. Agar plugs were removed from the actively growing edges of 7-day-old cultures with a 5 mm diam. cork borer and transferred to the centres of 90 mm Petri dishes containing MEA. Cultures were grown at seven different temperatures ranging from 5 °C to 35 °C, at 5 °C intervals with five replicates per isolate. Colony diameters were measured after seven days. Colony colours were described using the colour charts of Rayner [56] using seven-day-old cultures on MEA incubated at 25 °C. All descriptions were deposited in MycoBank (www.mycobank.org, accessed on 3 October 2021).

## 3. Results

### 3.1. Sample Collection and Fungal Isolation

A total of 209 soil samples were collected and 353 isolates having a morphology typical of *Calonectria* were isolated from 79 of these samples (Table 1, Appendix A Table A1). Of these, 121 soil samples were from seven *Eucalyptus* plantations, of which 57 samples yielded 253 *Calonectria* isolates. Forty-three soil samples were collected from four natural forests, of which 14 samples yielded 61 *Calonectria* isolates; 21 soil samples were collected from two *C. lanceolata* plantations, two of which yielded nine *Calonectria* isolates; and 14 soil samples collected from a single *P. heterocycle* plantation, of which five samples yielded 25 *Calonectria* isolates. In addition, ten soil samples were collected from the *Pi. massoniana* plantation, only one of which yielded five *Calonectria* isolates (Table 1).

### 3.2. Phylogenetic Analyses

The *tef1* fragment was amplified for all of the 353 isolates (Appendix A Table A1), and based on sequence differences for this region and the sampling sites, 144 isolates were selected to amplify the *cmdA*, *his3* and *tub2* gene regions. Subsequently, based on the 37 genotypes revealed by these four gene regions, 71 representative isolates were chosen to amplify the *act* and *rpb2* gene regions (Appendix A Table A1). All of the 71 isolates, representing the 40 genotypes determined from the sequence data for the six gene regions, were used for phylogenetic inference (Table 2). Amplicons generated for the *act*, *cmdA*, *his3*, *rpb2*, *tef1*, and *tub2* gene regions were approximately 300, 700, 500, 860, 550, and 600 bp, respectively.

**Figure 3:**
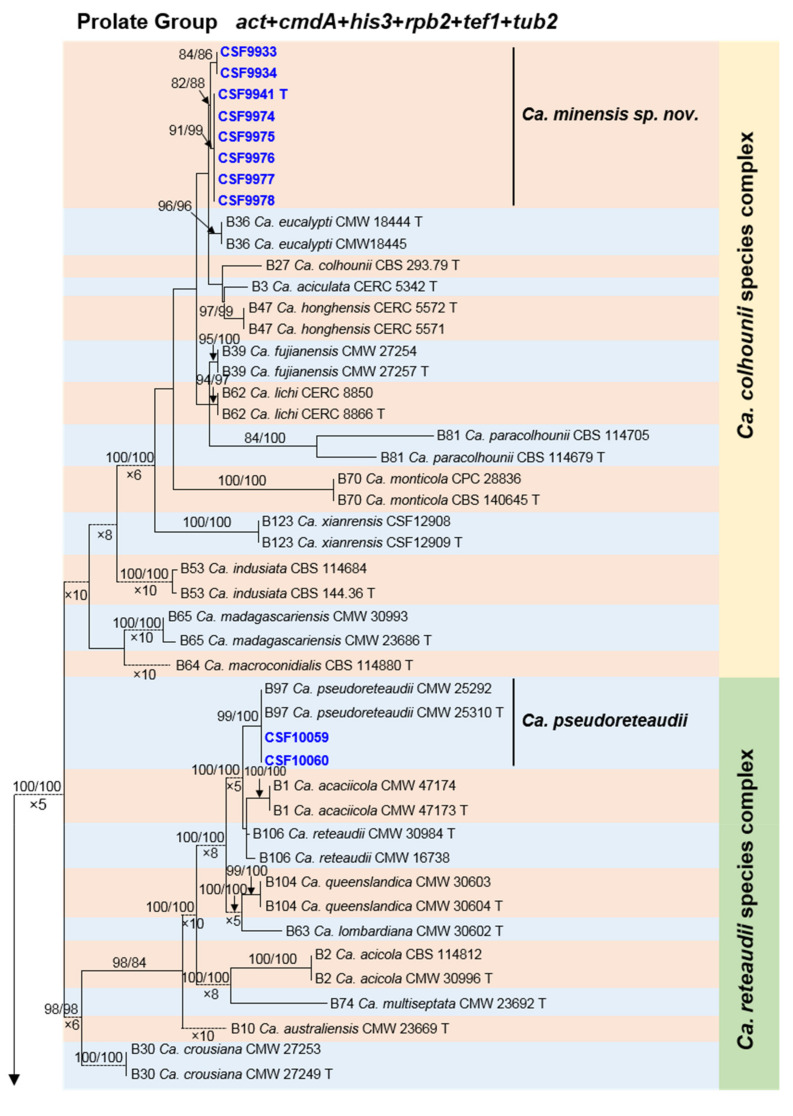

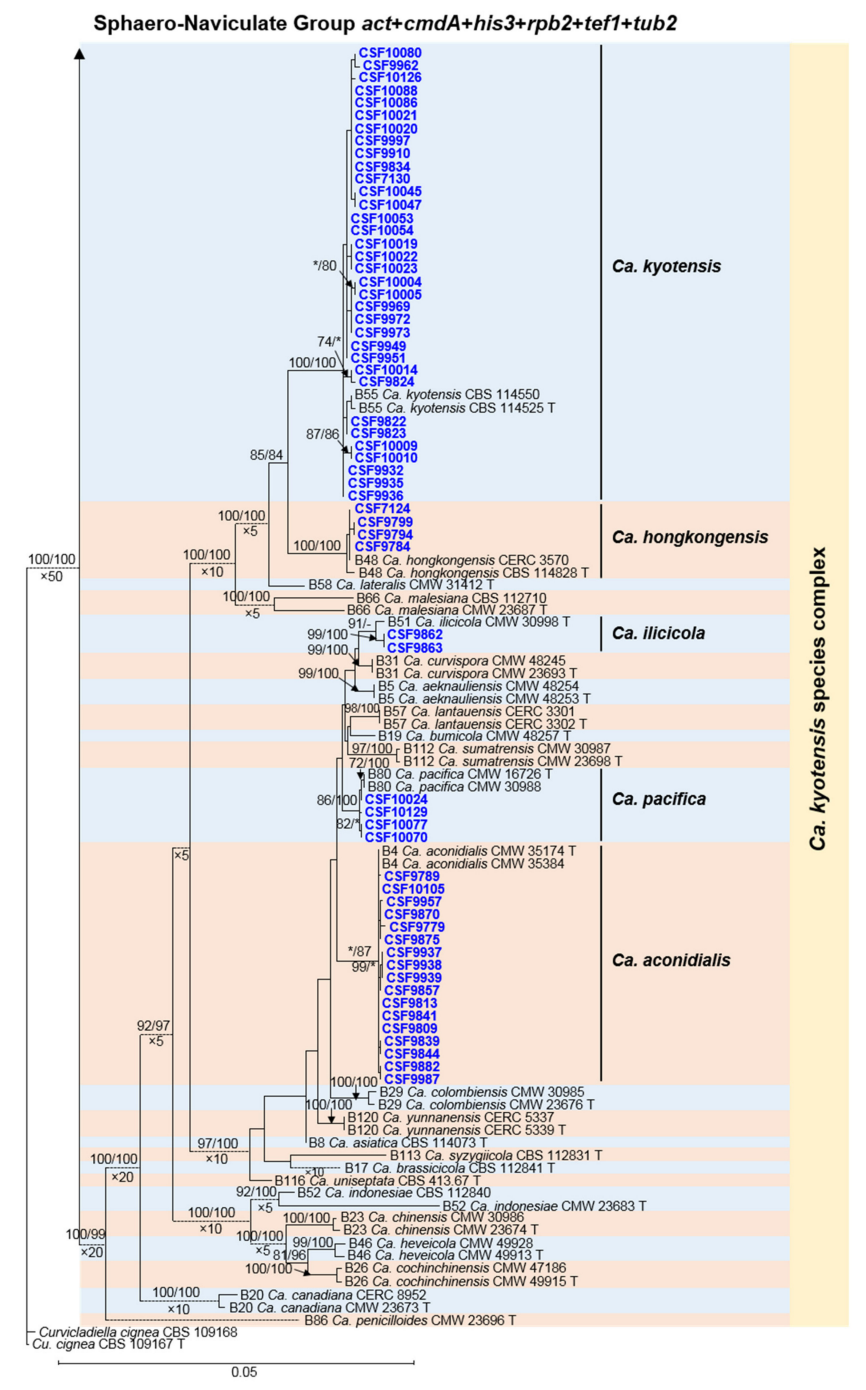
Phylogenetic tree of *Calonectria* species based on maximum likelihood (ML) analyses of combined DNA dataset of *act*, *cmdA*, *his3*, *rpb2*, *tef1*, and *tub2* gene sequences. Bootstrap value ≥70% for ML and MP analyses are presented above the branches. Bootstrap values lower than 70% are marked with “*”, and absent analyses values are marked with “-”. Ex-type isolates are marked with “T”. Isolates sequenced in this study are highlighted in blue and bold type. The “B” species codes are consistent with the recently published results in Liu and co-authors [18]. The tree was rooted to *Curvicladiella cignea* (CBS 109167 and CBS 109168).

**Figure A1:**
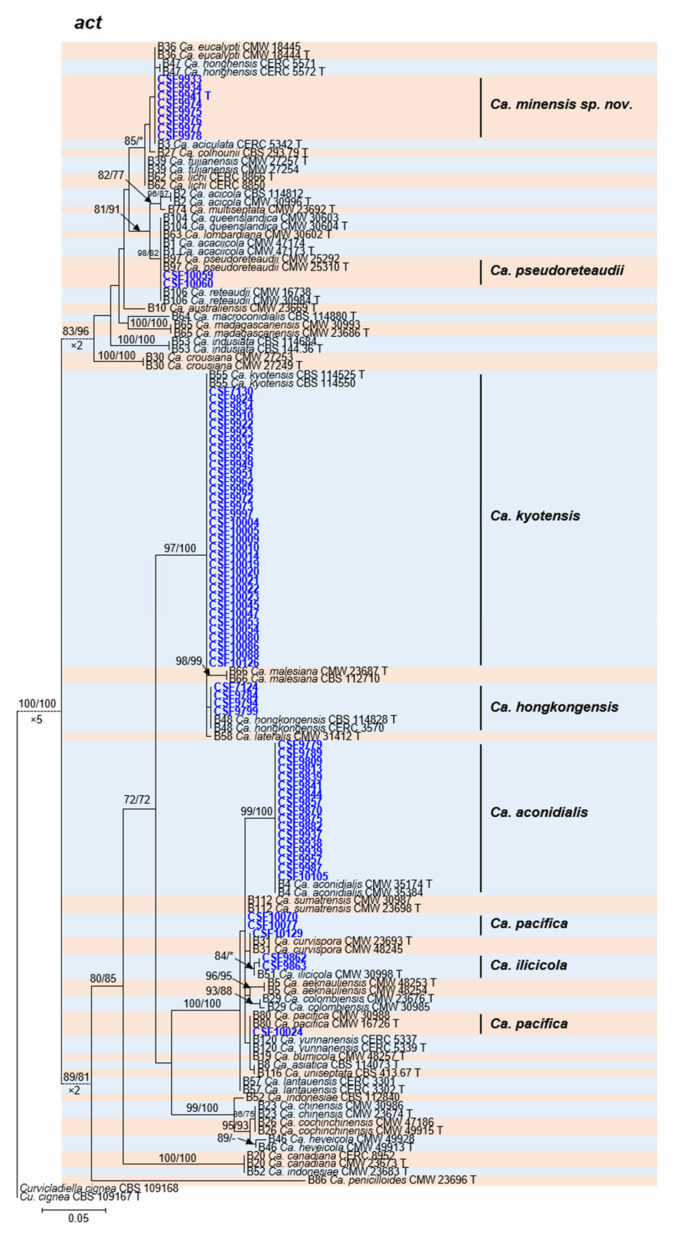
Phylogenetic tree of *Calonectria* species based on maximum likelihood (ML) analyses of *act* gene sequences. Bootstrap value ≥70% for ML and MP analyses are presented at the branches. Bootstrap values lower than 70% are marked with “*”, and absent analyses values are marked with “-”. Ex-type isolates are marked with “T”. Isolates sequenced in this study are highlighted in blue and bold type. The “B” species codes are consistent with the recently published results in Liu and co-authors [18]. The tree was rooted to *Curvicladiella cignea* (CBS 109167 and CBS 109168).

**Figure A2:**
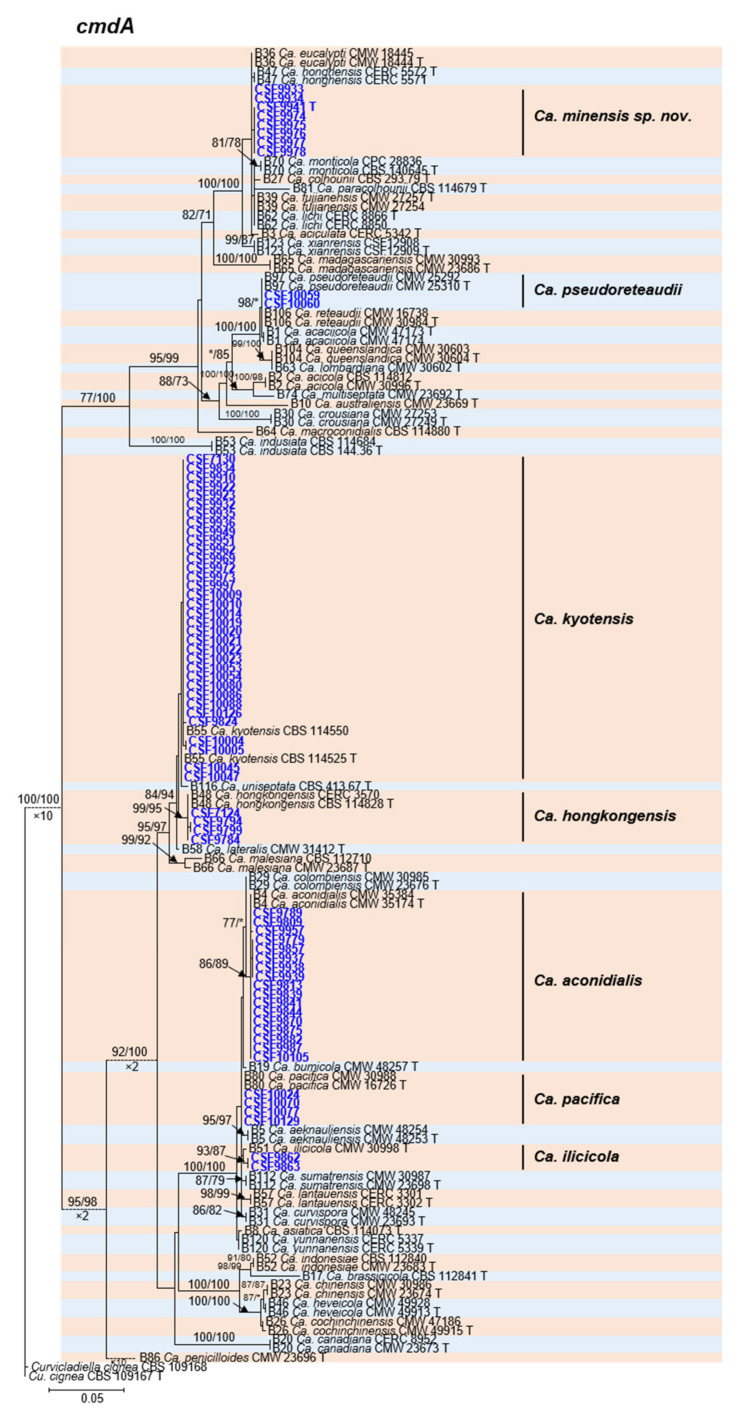
Phylogenetic tree of *Calonectria* species based on maximum likelihood (ML) analyses of *cmdA* gene sequences. Bootstrap value ≥70% for ML and MP analyses are presented at the branches. Bootstrap values lower than 70% are marked with “*”, and absent analyses values are marked with “-”. Ex-type isolates are marked with “T”. Isolates sequenced in this study are highlighted in blue and bold type. The “B” species codes are consistent with the recently published results in Liu and co-authors [18]. The tree was rooted to *Curvicladiella cignea* (CBS 109167 and CBS 109168).

**Figure A3:**
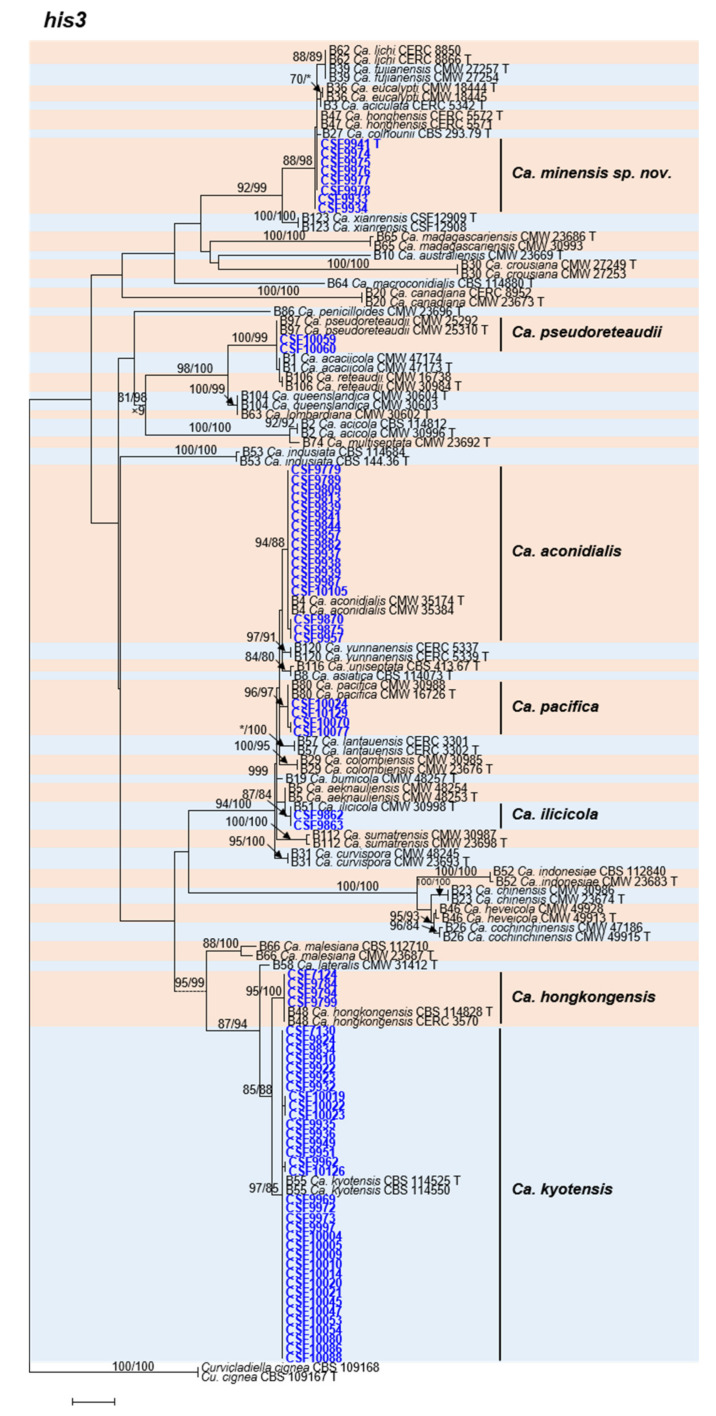
Phylogenetic tree of *Calonectria* species based on maximum likelihood (ML) analyses of *his3* gene sequences. Bootstrap value ≥70% for ML and MP analyses are presented at the branches. Bootstrap values lower than 70% are marked with “*”, and absent analyses values are marked with “-”. Ex-type isolates are marked with “T”. Isolates sequenced in this study are highlighted in blue and bold type. The “B” species codes are consistent with the recently published results in Liu and co-authors [18]. The tree was rooted to *Curvicladiella cignea* (CBS 109167 and CBS 109168).

**Figure A4:**
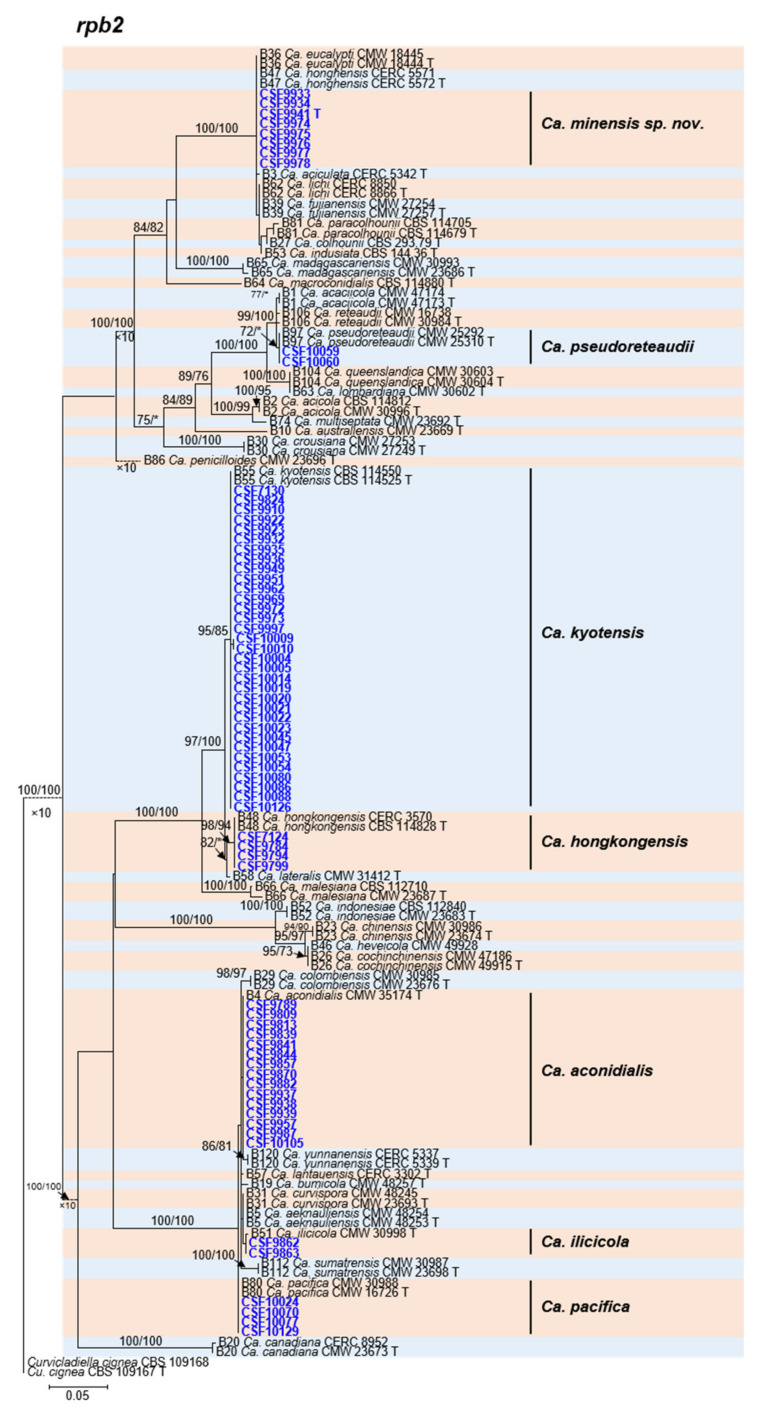
Phylogenetic tree of *Calonectria* species based on maximum likelihood (ML) analyses of *rpb2* gene sequences. Bootstrap value ≥70% for ML and MP analyses are presented at the branches. Bootstrap values lower than 70% are marked with “*”, and absent analyses values are marked with “-”. Ex-type isolates are marked with “T”. Isolates sequenced in this study are highlighted in blue and bold type. The “B” species codes are consistent with the recently published results in Liu and co-authors [18]. The tree was rooted to *Curvicladiella cignea* (CBS 109167 and CBS 109168).

**Figure A5:**
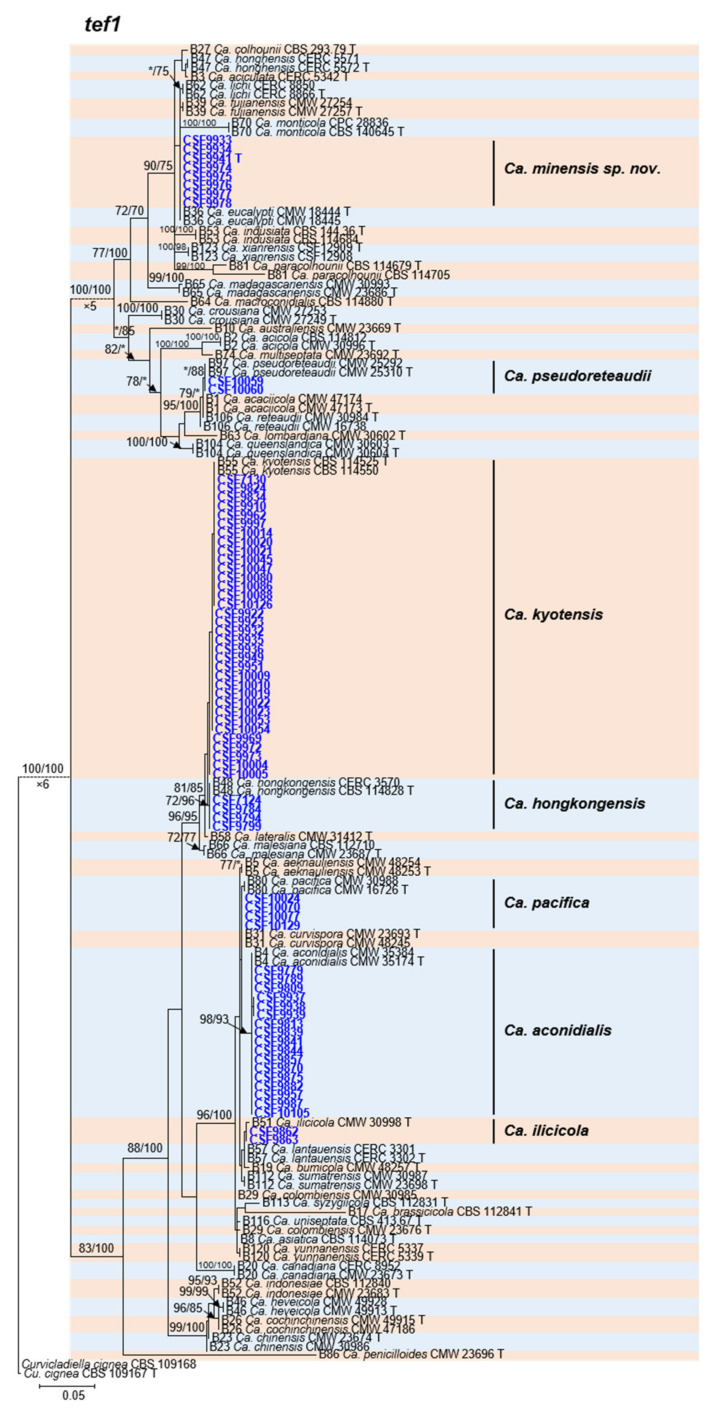
Phylogenetic tree of *Calonectria* species based on maximum likelihood (ML) analyses of *tef1* gene sequences. Bootstrap value ≥70% for ML and MP analyses are presented at the branches. Bootstrap values lower than 70% are marked with “*”, and absent analyses values are marked with “-”. Ex-type isolates are marked with “T”. Isolates sequenced in this study are highlighted in blue and bold type. The “B” species codes are consistent with the recently published results in Liu and co-authors [18]. The tree was rooted to *Curvicladiella cignea* (CBS 109167 and CBS 109168).

**Figure A6:**
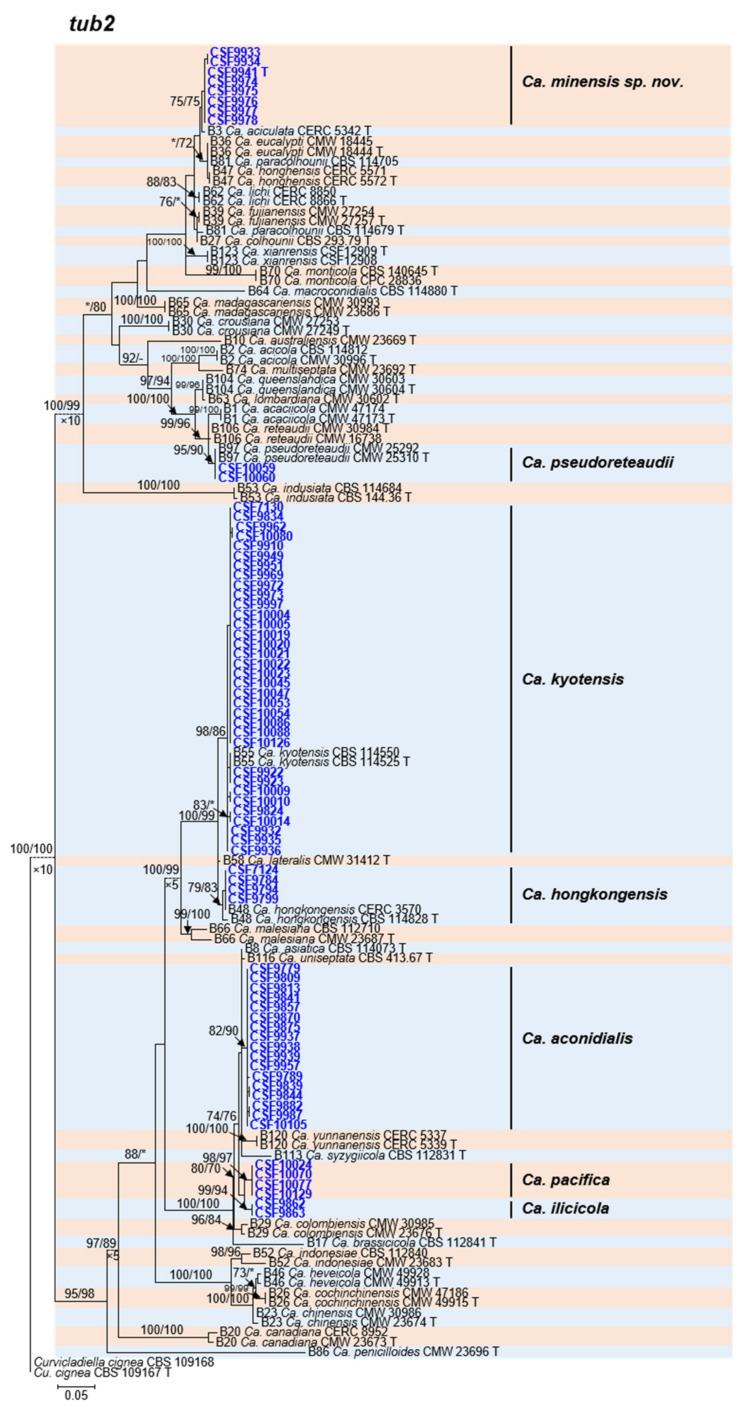
Phylogenetic tree of *Calonectria* species based on maximum likelihood (ML) analyses of *tub2* gene sequences. Bootstrap value ≥70% for ML and MP analyses are presented at the branches. Bootstrap values lower than 70% are marked with “*”, and absent analyses values are marked with “-”. Ex-type isolates are marked with “T”. Isolates sequenced in this study are highlighted in blue and bold type. The “B” species codes are consistent with the recently published results in Liu and co-authors [18]. The tree was rooted to *Curvicladiella cignea* (CBS 109167 and CBS 109168).

Sequence data for 46 *Calonectria* species closely related to those collected in this study were downloaded from GenBank and a total of 78 sequences (for ex-type and other strains) from previous studies were included in the phylogenetic analyses (Table 3). Phylogenetic analyses based on the six individual gene regions and the concatenated dataset for those regions were conducted using both MP and ML methods. The results showed that the overall topologies generated from the MP analyses were essentially similar to those from the ML analyses, and consequently, only the ML trees are presented (Figure 3, Appendix B Figure A1, Figure A2, Figure A3, Figure A4, Figure A5 and Figure A6).

The partition homogeneity test carried out on the datasets, for the combined six gene regions, generated *p* values of 0.001. This showed that the accuracy of the combined data did not suffer relative to the individual partitions [57]. Sequence data for the six gene regions were thus combined for analyses. The sequence alignments based on the individual six gene regions and the combination of these were deposited in TreeBASE (No. S28845). Statistics and important parameters emerging from the phylogenetic analyses are presented in Table 4.

Based on the six-gene combined phylogenetic tree (Figure 3), for the 71 isolates used in the phylogenetic analyses, eight isolates resided in the *Ca*. *colhounii* species complex, two isolates in the *Ca*. *reteaudii* species complex and 61 isolates in the *Ca*. *kyotensis* species complex.

### 3.3. Species in the Calonectria colhounii Species Complex

Six isolates (CSF9941, CSF9974, CSF9975, CSF9976, CSF9977 and CSF9978), representing one genotype, formed a distinct lineage in the *cmdA* and *tub2* analyses as well as in the six-gene combined phylogenetic tree (Figure 3, Appendix B Figure A2 and Figure A6). The total number of SNP differences between the six isolates and other phylogenetically closely related species [*Ca. aciculata* (ex-type isolate CERC 5342), *Ca. colhounii* (ex-type isolate CBS 293.79), *Ca. eucalypti* (ex-type isolate CMW 18444) and *Ca. honghensis* (ex-type isolate CERC 5572)] for six gene regions combined, varied between 13 and 31. Thus, this fungus can be regarded as a novel species. Two isolates (CSF9933 and CSF9934) formed an independent clade and were phylogenetically most closely related to the six isolates in the six-gene phylogenetic tree (Figure 3). These two isolates were consequently considered as the same species as the six isolates CSF9941, CSF9974, CSF9975, CSF9976, CSF9977 and CSF9978 and were identified as the novel species.

### 3.4. Species in the Calonectria reteaudii Species Complex

Two isolates (CSF10059 and CSF10060) were phylogenetically closely related to *Ca. pseudoreteaudii* and various other species based on *act* and *cmdA* trees (Appendix B Figure A1 and Figure A2), and clustered with *Ca. pseudoreteaudii* based on *his3*, *rpb2*, *tef1*, *tub2* and the six-gene combined trees (Figure 3, Appendix B Figure A3, Figure A4, Figure A5 and Figure A6). In comparisons of DNA sequences for these six gene regions, all the sequences for the two isolates (CSF10059 and CSF10060) were 100% identical to the ex-type isolate (CMW 25310) of *Ca. pseudoreteaudii*. Consequently, they were identified as *Ca. pseudoreteaudii* (Figure 3).

### 3.5. Species in the Calonectria kyotensis Species Complex

Thirty-four isolates representing 20 genotypes were phylogenetically closest to *Ca. kyotensis* in each of the *cmdA*, *his3*, *rpb2* (sequence data for the *rpb2* were not available for isolate CSF9834), *tef1*, *tub2* and the six-gene combined trees (Figure 3, Appendix B Figure A2, Figure A3, Figure A4, Figure A5 and Figure A6), and clustered with *Ca. kyotensis* based on the *act* tree (Appendix B Figure A1). Some isolates formed distinct clades based on the six-gene combined trees (Figure 3), while the total number of SNP differences between the 34 isolates and the ex-type isolate of *Ca. kyotensis* (CBS 114525) for six gene regions combined varied between 2 and 8. Based on the phylogenetic analyses, these 34 isolates were identified as *Ca. kyotensis*.

Four isolates (CSF7124, CSF9784, CSF9794 and CSF9799), representing two genotypes, were phylogenetically closest to *Ca. hongkongensis* in each of the *cmdA*, *tub2* and six-gene combined tree (Figure 3, Appendix B Figure A2 and Figure A6), and clustered with *Ca. hongkongensis* based on *act*, *his3*, *rpb2* and *tef1* trees (Appendix B Figure A1, Figure A3, Figure A4 and Figure A5). There were only three or four SNP differences between these four isolates and the ex-type isolate of *Ca. hongkongensis* (CBS 114828) when sequences for six gene regions were combined. Thus, these four isolates were identified as *Ca. hongkongensis*.

Two isolates (CSF9862 and CSF9863), representing one genotype clustered with *Ca. ilicicola* in the *his3* tree (Appendix B Figure A3), formed independent clades but closely related to *Ca. ilicicola* in the *act*, *cmdA*, *rpb2*, *tef1* and six-gene combined trees (Figure 3, Appendix B Figure A1, Figure A2, Figure A4, Figure A5 and Figure A6). There were only six SNP differences between the two isolates and the ex-type isolate of *Ca. ilicicola* (CMW 30998) for five gene regions (*tub2* sequence data were not available for *Ca. ilicicola*) combined. Consequently, these isolates were regarded as *Ca. ilicicola*.

Four isolates (CSF10024, CSF10070, CSF10077 and CSF10129), representing three genotypes, were phylogenetically related to *Ca. pacifica* and various other closely related species based on *act* and *tef1* trees (Appendix B Figure A1 and Figure A5). They were, however, phylogenetically closest to *Ca. pacifica* based on *his3* and six-gene combined trees (Appendix B Figure A3), and clustered with *Ca. pacifica* based on *cmdA* and *rpb2* trees (Appendix B Figure A2 and Figure A4). There were only one or three SNP difference(s) between the four isolates and the ex-type isolate of *Ca. pacifica* (CMW 16726) for five gene regions (*tub2* sequence data were not available for *Ca. pacifica*) combined. These four isolates were thus identified as *Ca. pacifica*.

Seventeen isolates representing 11 genotypes were phylogenetically closest to *Ca. aconidialis* based on *cmdA*, *his3*, *tef1* and six-gene combined trees (Figure 3, Appendix B Figure A2, Figure A3 and Figure A5), and clustered with *Ca. aconidialis* based on *act* and *rpb2* (*rpb2* sequence data were not available for CSF9779 and CSF9875) trees (Appendix B Figure A1 and Figure A4). Some isolates formed distinct clades based on the six-gene combined trees (Figure 3), while the total number of SNP differences between the 17 isolates and the ex-type isolate of *Ca. aconidialis* (CMW 35174) for five gene regions (sequence data for the *tub2* region were not available for *Ca. aconidialis*) combined varied between 0 and 4. Therefore, the 17 isolates were identified as *Ca. aconidialis*.

Seventy-one of the 353 isolates collected in this study were identified based on the DNA sequence of the six gene regions. According to the species identification results, we further identified the remaining 282 isolates based on the DNA sequences for two or four gene regions (Appendix A Table A1). Consequently, for the entire collection of 353 isolates, these were identified as *Ca. aconidialis* (178), *Ca. kyotensis* (103), *Ca. hongkongensis* (37), *Ca. pacifica* (17), *Ca. ilicicola* (five), *Ca. pseudoreteaudii* (five) and a novel species (eight), respectively.

### 3.6. Sexual Compatibility

Three isolates (CSF9933, CSF9941 and CSF9975) of the novel species were used in the mating tests (Table 2). All of these isolates formed protoperithecia readily within two weeks, and perithecia with viable ascospores were produced within four weeks. This was irrespective of whether they were crossed with each other or with themselves. The species was thus shown to be homothallic.

### 3.7. Morphology and Taxonomy

**Figure A7:**
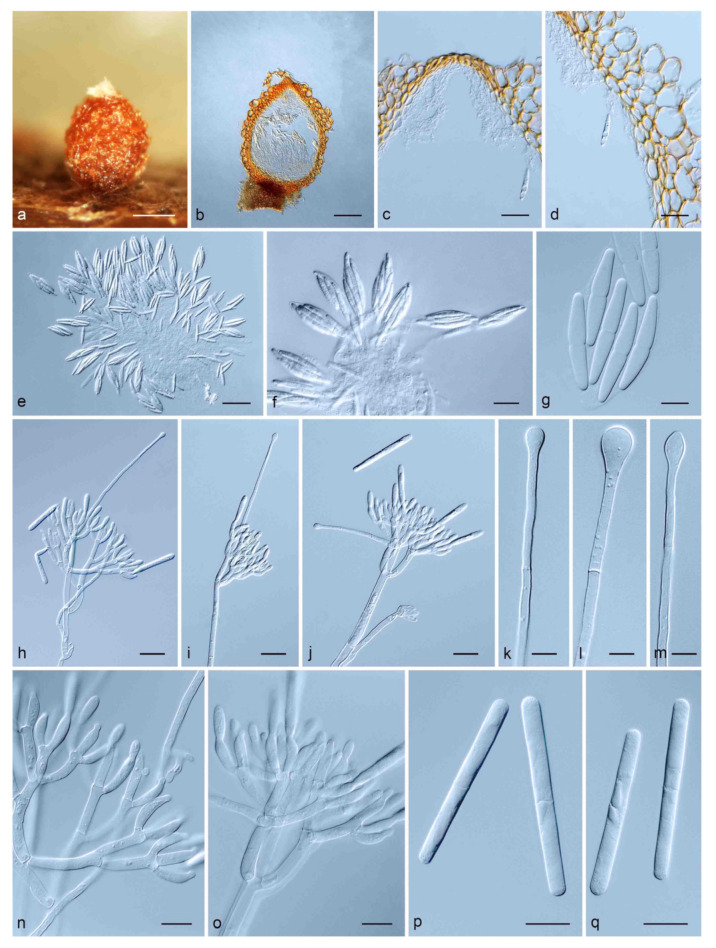
*Calonectria aconidialis*. (**a**). Perithecium; (**b**). vertical section through a perithecium; (**c**). cells around ostiolar region of perithecium; (**d**). section through lateral perithecial wall; (**e**,**f**). asci; (**g**). ascospores; (**h**–**j**). macroconidiophore; (**k**–**m**). obpyriform to sphaeropedunculate vesicles; (**n**,**o**). conidiogenous apparatus with conidiophore branches and elongate doliiform to reniform phialides; (**p**,**q**). macroconidia.—Scale bars: a = 200 μm; b = 100 μm; c, d, f and h–j = 20 μm; e = 50 μm; g and n–q = 10 μm; k–m = 5 μm.

**Figure A8:**
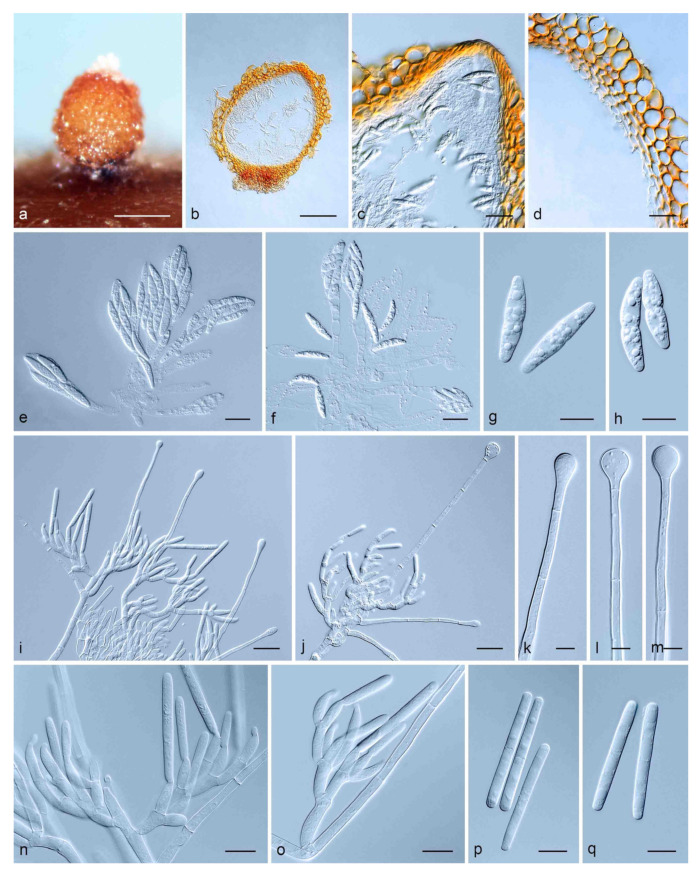
*Calonectria hongkongensis*. (**a**). Perithecium; (**b**). vertical section through a perithecium; (**c**). cells around ostiolar region of perithecium; (**d**). section through lateral perithecial wall; (**e**,**f**). asci; (**g**,**h**). ascospores; (**i**,**j**). macroconidiophore; (**k**–**m**). sphaeropedunculate vesicles; (**n**,**o**). conidiogenous apparatus with conidiophore branches and elongate doliiform to reniform phialides; (**p**,**q**). macroconidia.—Scale bars: a = 200 μm; b = 100 μm; c–f and i,j = 20 μm; g,h and n–q = 10 μm; k–m = 5 μm.

**Figure A9:**
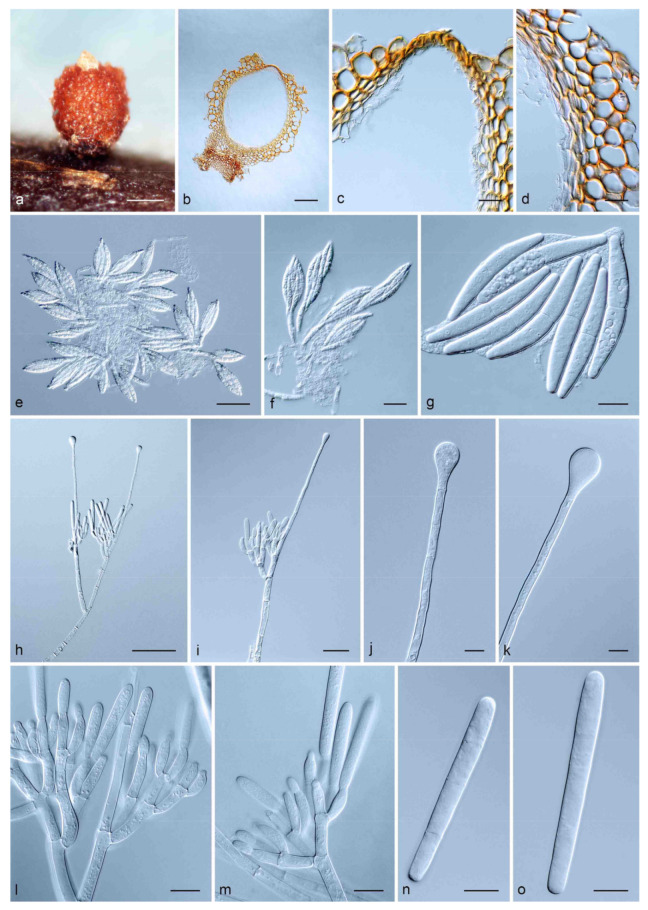
*Calonectria ilicicola*. (**a**). Perithecium; (**b**). vertical section through a perithecium; (**c**). cells around ostiolar region of perithecium; (**d**). section through lateral perithecial wall; (**e**,**f**). asci; (**g**). ascospores; (**h**,**i**). macroconidiophore; (**j**,**k**). ovoid to sphaeropedunculate vesicles; (**l**,**m**). conidiogenous apparatus with conidiophore branches and elongate doliiform to reniform phialides; (**n**,**o**). macroconidia.—Scale bars: a = 200 μm; b = 100 μm; c, d, f and i = 20 μm; e and h = 50 μm; g and l–o = 10 μm; j, k = 5 μm.

**Figure A10:**
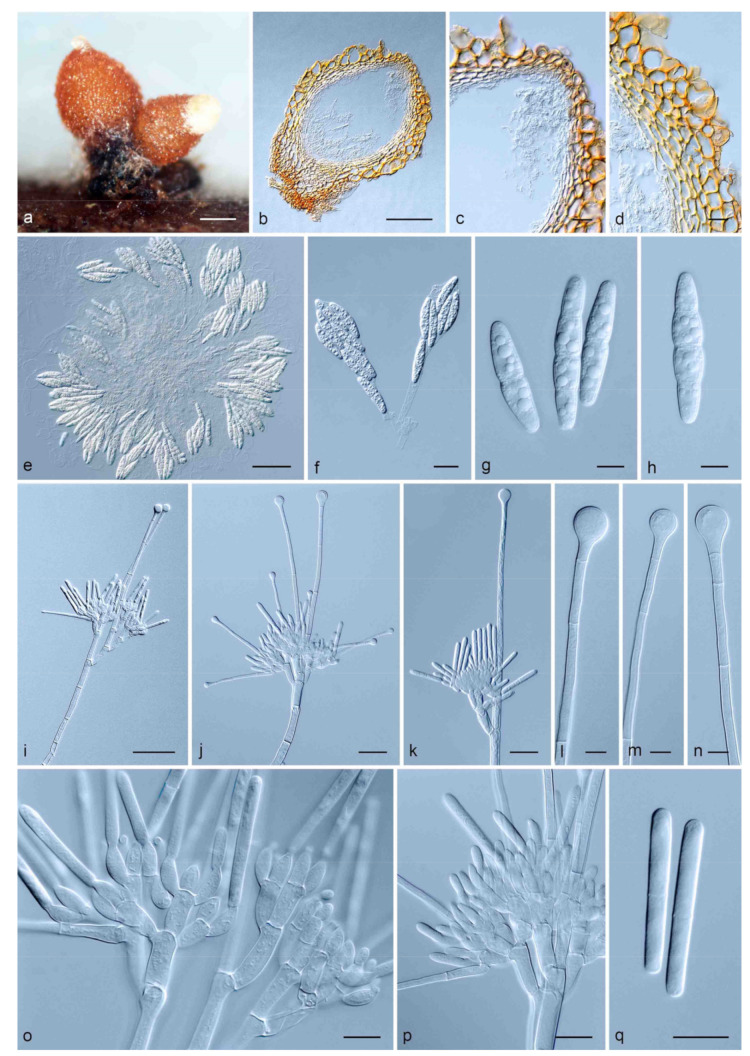
*Calonectria kyotensis*. (**a**). Perithecium; (**b**). vertical section through a perithecium; (**c**). cells around ostiolar region of perithecium; (**d**). section through lateral perithecial wall; (**e**,**f**). asci; (**g**,**h**). ascospores; (**i**–**k**). macroconidiophore; (**l**–**n**). sphaeropedunculate vesicles; (**o**,**p**). conidiogenous apparatus with conidiophore branches and elongate doliiform to reniform phialides; (**q**). macroconidia.—Scale bars: a = 200 μm; b = 100 μm; c, d, f, j and k = 20 μm; e and i = 50 μm; g, h and o–q = 10 μm; l–n = 5 μm.

**Figure A11:**
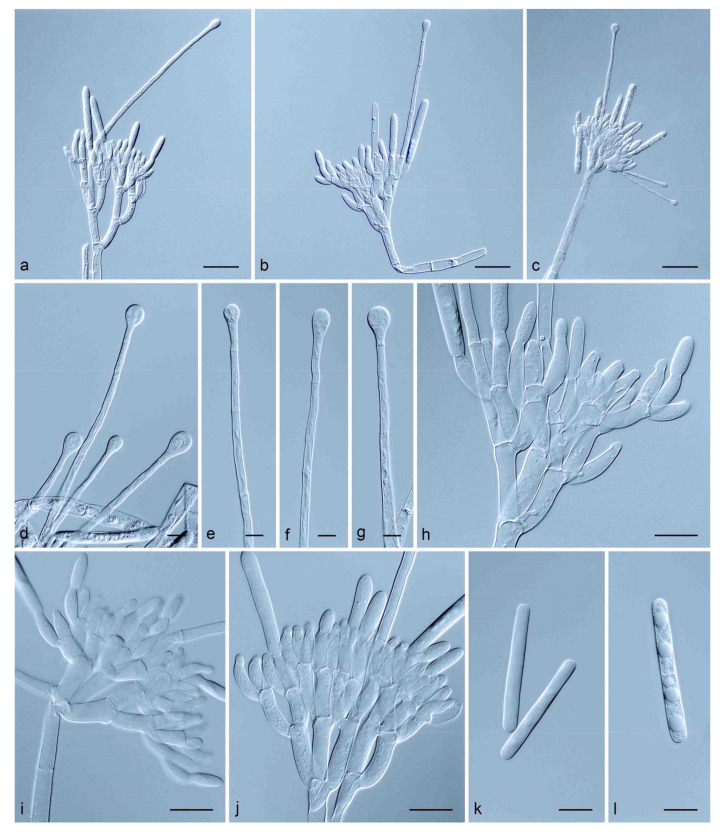
*Calonectria pacifica*. (**a**–**c**). Macroconidiophore; (**d**–**g**). ovoid to sphaeropedunculate vesicles; (**h**–**j**). conidiogenous apparatus with conidiophore branches and doliiform to reniform phialides; (**k**,**l**). macroconidia.—Scale bars: a–c = 20 μm; d–g = 5 μm; h–l = 10 μm.

**Figure A12:**
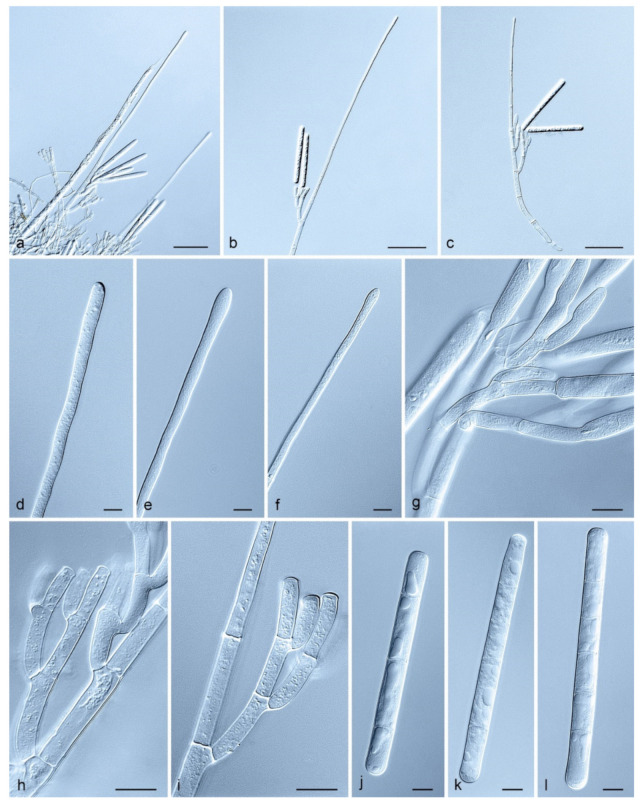
*Calonectria pseudoreteaudii*. (**a**–**c**). Macroconidiophore; (**d**–**f**). clavate to narrowly clavate vesicle; (**g**–**i**) conidiogenous apparatus with conidiophore branches and cylindrical to allantoid phialides; (**j**–**l**). macroconidia.—Scale bars: a–c = 20 μm; d–f = 5 μm; g–l = 10 μm.

Based on multi-gene phylogenetic analyses (Figure 3, Appendix B Figure A1, Figure A2, Figure A3, Figure A4, Figure A5 and Figure A6) and morphological characteristics, seven *Calonectria* species were identified in this study, including six described species, i.e., *Ca. aconidialis*, *Ca. kyotensis*, *Ca. hongkongensis*, *Ca. pacifica*, *Ca. ilicicola*, *Ca. pseudoreteaudii* and one novel species. To facilitate future studies, complete morphological descriptions and illustrations have been made for the known species and these are presented in Appendix C (Figure A7, Figure A8, Figure A9, Figure A10, Figure A11 and Figure A12). The novel species can be distinguished from the phylogenetically most closely related species (*Ca. aciculata*, *Ca. colhounii*, *Ca. eucalypti* and *Ca. honghensis*) by the dimensions of its macroconidia and ascospores (Table 5). This species is described as follows:

#### Taxonomy

***Calonectria minensis*** Q.L. Liu and S.F. Chen, sp. nov.

**Figure 4:**
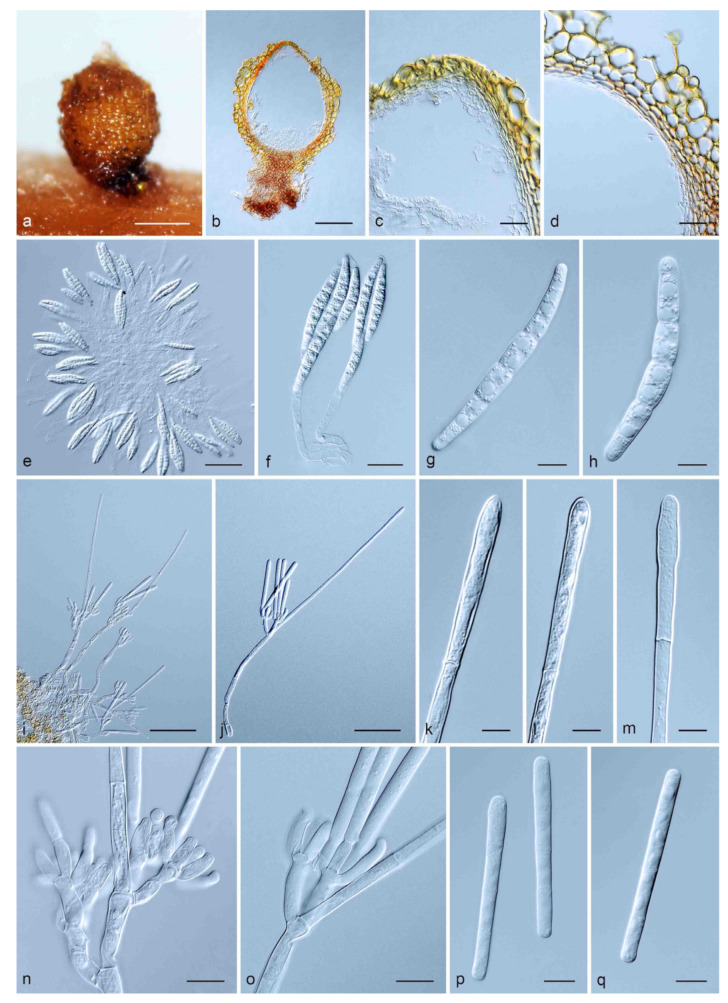
*Calonectria minensis*. (**a**) Perithecium; (**b**) vertical section through a perithecium; (**c**) cells around ostiolar region of perithecium; (**d**) section through lateral perithecial wall; (**e**,**f**) asci; (**g**,**h**) ascospores; (**i**,**j**) macroconidiophore; (**k**,**m**) clavate vesicles; (**n**,**o**) conidiogenous apparatus with conidiophore branches and elongate doliiform to reniform phialides; (**p**,**q**) macroconidia.—Scale bars: a = 200 μm; b = 100 μm; c, d and f = 20 μm; e and i, j = 50 μm; g, h and n–q = 10 μm; k, m = 5 μm.

MycoBank MB841412. (Figure 4).

Etymology: Name refers to the short name of Fujian Province in Chinese “Min”, where this fungus was isolated.

Diagnosis: *Calonectria minensis* can be distinguished from the phylogenetically closely related species *Ca. aciculata*, *Ca. colhounii*, *Ca. eucalypti* and *Ca. honghensis* by its distinct ascospore and macroconidia dimensions.

Type: China: Fujian Province, Longyan Region, Xinluo District (25°07′08.597″ N, 116°44′42.257″ E), from soil collected in a *Eucalyptus* plantation, 6 November 2016, *S.F. Chen*, *Q.L. Liu* and *F.F. Liu* (HMAS249935–—holotype, CSF9941 = CGMCC3.18877—ex-type culture).

Description: *Ascomata* perithecial, solitary or in groups of four, bright yellow, becoming orange with age; in section, apex and body yellow, base red-brown, sub-globose to ovoid, 258–395 μm high, 227–330 μm diam, body turning dark yellow, and base dark red-brown in 3% KOH+; ascomatal wall rough, consisting of two thick-walled layers; outer layer of *textura globulosa*, 22–66 μm thick, cells becoming more compressed towards the inner layer of *textura angularis*, 9–21 μm thick, cells becoming thin-walled and hyaline towards the centre; outermost cells 16–31 × 8–16 μm, cells of inner layer 8–33 × 2–8 μm; ascomatal base up to 196 μm wide, consisting of dark red, angular cells, merging with an erumpent stroma; cells of the outer wall layer continuous with the pseudoparenchymatous cells of the erumpent stroma. *Asci* 4-spored, clavate, 80–163 × 11–27 μm, tapering into a long thin stalk. *Ascospores* aggregated in the upper third of the ascus, hyaline, guttulate, fusoid with rounded ends, straight to slightly curved, (1–)3-septate, constricted at the septum, (38.5–)46.5–64.5(–80.5) × (6–)6.5–8(–8.5) μm (av. = 55.5 × 7 μm). *Macroconidiophores* consisting of a stipe, a suite of penicillately arranged fertile branches, a stipe extension, and a terminal vesicle; stipe septate, hyaline, smooth, 33–144 × 4–9 μm, stipe extension septate, straight to flexuous 63–240 μm long, 2–3 μm wide at the apical septum, terminating in a clavate vesicle, 3–5 μm diam; lateral stipe extensions (90° to main axis) absent. *Conidiogenous apparatus* 28–97 μm wide, and 35–83 μm long; primary branches aseptate, 13–40 × 3–7 μm; secondary branches aseptate, 9–31 × 3–6 μm; tertiary branches aseptate, 8–14 × 3–5 μm, quaternary branches aseptate, 7−12 × 3–5 μm, each terminal branch producing 2–4 phialides; phialides allantoid to elongate doliiform to reniform, hyaline, aseptate, 4–14 × 2–7 μm, apex with minute periclinal thickening and inconspicuous collarette. *Macroconidia* cylindrical, rounded at both ends, straight, (51–)55–66(–79) × (4.5–)5–6(–7.5) μm (av. = 60.5 × 5.5 μm), (1–)3-septate, lacking a visible abscission scar, held in parallel cylindrical clusters by colourless slime. Mega- and microconidia not observed.

Culture characteristics: Colonies forming abundant woolly white to sienna (8) aerial mycelium at 25 °C on MEA, profuse sporulation; surface rust-coloured (39); reverse sienna (8) to rust-coloured (39) after 7 d. Chlamydospores extensive throughout the medium forming microsclerotia. Optimal growth temperature 25 °C, no growth at 5 °C and 35 °C, after 7 d, colonies at 10 °C, 15 °C, 20 °C, 25 °C and 30 °C reached 18.1 mm, 27.0 mm, 58.2 mm, 69.5 mm and 42.4 mm, respectively.

Additional specimens examined: China: Fujian Province, Longyan Region, Xinluo District (25°07′08.597″ N, 116°44′42.257″ E), from soil collected in a *Eucalyptus* plantation, 6 November 2016, S.F. Chen, Q.L. Liu and F.F. Liu (HMAS249936, culture CSF9933 = CGMCC3.18875); Fujian Province, Longyan Region, Liancheng County (25°26′14.348″ N, 116°38′42.400″ E), from soil under a natural forest, 6 November 2016, S.F. Chen, Q.L. Liu and F.F. Liu (HMAS249937, culture CSF9975 = CGMCC3.18881).

Notes: *Calonectria minensis* is a new species in the *Ca. colhounii* species complex. It is closely related to *Ca. aciculata*, *Ca. colhounii*, *Ca. eucalypti*, and *Ca. honghensis*, and can be distinguished from those species by the dimensions of its ascospores and macroconidia. The ascospores of *Ca. minensis* (av. = 55.5 × 7 µm) are larger than those of *Ca. eucalypti* (av. = 33 × 6 µm) [37] and *Ca. honghensis* (av. = 49 × 6 µm) [4]. The macroconidia of *Ca. minensis* (av. = 60.5 × 5.5 µm) are shorter than those of *Ca. aciculata* (av. = 69 × 5.5 µm) [4], *Ca. colhounii* (av. = 65 × 5 µm) [17] and *Ca. eucalypti* (av. = 72 × 6 µm) [37], but longer than those of *Ca. honghensis* (av. = 54 × 5.5 µm) [4]. The total number of SNP differences between the ex-type isolate of *Ca. minensis* (CSF9941), and the ex-type isolates of *Ca. aciculata* (CERC 5342), *Ca. colhounii* (CBS 293.79), *Ca. eucalypti* (CMW 18444) and *Ca. honghensis* (CERC 5572) for six gene regions combined, varied between 13 and 31.

### 3.8. Distribution of Calonectria Species in Fujian Province

**Figure 5:**
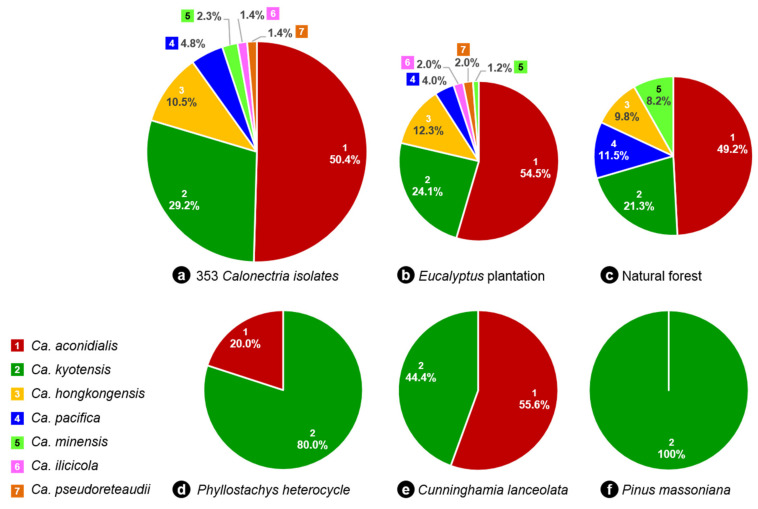
*Calonectria* species collected from soils of five different types of forests in Fujian Province. (**a**). the percentage of each *Calonectria* species accounted for all of the species isolated in this study. Different species are indicated by numbers with different colours; (**b**–**f**). the percentage of each *Calonectria* species obtained from five different types of forests.

Of the seven *Calonectria* species identified, *Ca. aconidialis* accounted for 50.4% of all the isolates. This was followed in order of occurrence by *Ca. kyotensis* (29.2%), *Ca. hongkongensis* (10.5%), *Ca. pacifica* (4.8%), *Ca. minensis* (2.3%), *Ca. ilicicola* (1.4%) and *Ca. pseudoreteaudii* (1.4%) (Figure 5). *Calonectria aconidialis* and *Ca. kyotensis* can be regarded as the most prevalent species (Figure 5).

Between two and four *Calonectria* species were isolated from soils sampled at each of the nine Counties or Districts (Figure 2). *Calonectria aconidialis* was found at all sites other than Cangshan District, *Ca. kyotensis* was found at all sites other than Yanping District and Zhangping County, and the remaining five species were found at between one and three sampling sites (Figure 2).

All seven species were isolated from soils collected in *Eucalyptus* plantations. Five of the species were isolated from soils in natural forests, the exception being *Ca. ilicicola* and *Ca. pesudoreteaudii*. Only *Ca. aconidialis* and *Ca. kyotensis* were isolated from soils in *P. heterocycle* and *C. lanceolata* plantations, and only *Ca. kyotensis* was collected from soils in the *Pi. massoniana* plantation (Figure 5). Based on the percentage of soil samples that obtained *Calonectria* from each of the five forest types, the results showed that *Calonectria* was widely distributed in *Eucalyptus* plantation soils (47.1%, 57 of 121 sampled soils), followed by *P. heterocycle* (35.7%, 5 of 14 sampled soils) and natural forests (32.6%, 14 of 43 sampled soils), only 10% of soil samples obtained *Calonectria* from *C. lanceolata* (2 of 21 sampled soils) or *Pi. massoniana* (1 of 10 sampled soils).

*Calonectria kyotensis* was detected in soils in all of the soil types sampled, while *Ca. aconidialis* was isolated from soils in all forest types other than *Pi. massoniana*. *Calonectria hongkongensis*, *Ca. pacifica* and *Ca. minensis* were found both in *Eucalyptus* plantations and natural forests and the remaining two species were found only in *Eucalyptus* plantations (Figure 5).

## 4. Discussion

A total of 353 *Calonectria* isolates were collected from soils in *Eucalyptus* plantations and adjacent plantations of other species or natural forests in Fujian Province. Multilocus phylogenetic inference and morphological characteristics revealed seven *Calonectria* species including *Ca. aconidialis*, *Ca. hongkongensis*, *Ca. ilicicola*, *Ca. kyotensis*, *Ca. pacifica* and *Ca. pseudoreteaudii*, and a novel species described here as *Ca. minensis*.

Results in this study showed that *Ca. aconidialis* and *Ca. kyotensis* were the most prevalent species in the soils sampled. *Calonectria aconidialis* accounted for 50.4% of all the isolates, which was found in eight of the nine sampled sites and soils of all forest types other than those of *Pi. massoniana*. The next most common species was *Ca. kyotensis*, accounting for 29.2% of the isolates, which was isolated from seven sites and soils of all five different forest types. The remaining five species were less common, and isolated only from one to three sites, either from *Eucalyptus* plantations or natural forests, or from both of these forest types.

Among the identified species, *Ca. aconidialis* is newly reported in Fujian Province and *Ca. pacifica* represents a first record for China. Eight *Calonectria* species were previously known in Fujian Province. These include *Ca. crousiana*, *Ca. eucalypti*, *Ca. fujianensis*, *Ca. pauciramosa* and *Ca. pseudoreteaudii* collected from diseased *Eucalyptus* leaves [7,8], *Ca. hongkongensis* and *Ca. kyotensis* isolated from soils in unknown forest types [4,18] and *Ca. ilicicola* collected from diseased peanuts (*Arachis hypogaea*) in Longyan Region [58].

The *Calonectria* species diversity in soils was clearly dependent on the forest types sampled. Of the seven species detected, all were obtained from *Eucalyptus* plantations, five were obtained from natural forests and only one or two species were from other forest types. While these observations are convincing in terms of broad patterns, they must be tempered by the fact that the greatest number of soil samples were from *Eucalyptus* plantations and natural forests, which could have influenced the results.

The newly described *Ca. minensis* isolated from soils both in *Eucalyptus* plantations and natural forest, adds a new species to the *Ca. colhounii* species complex. As a consequence, 13 species are now accommodated in this complex [4,7,17,18,21,25,37,39,46,49]. With the exception of *Ca. macroconidialis* [46], *Ca. madagascariensis* [17] and *Ca. paracolhounii* [39], all of the other 10 species have been recorded in southeastern Asia [4,7,17,21,25]. Species in this complex include some important causal agents of CLB on *Eucalyptus* spp. including *Ca. aciculata*, *Ca. eucalypti* and *Ca. fujianensis*, which have all been reported from diseased *Eucalyptus* trees in China plantations [4,7].

Five species residing in the *Ca. kyotensis* species complex were identified in the present study. Of these, *Ca. aconidialis* accounted for more than half of all the isolates collected, and has previously been shown to be widely distributed in soils of *Eucalyptus* plantation in many regions of southern China, including Guangdong [11,18], Guangxi [4,10,11] and Hainan Provinces [11]. In the present study, *Ca. aconidialis* was collected from soils of four types of forests and in eight of the nine sampling sites in Fujian Province (Figure 2), providing new geographic records for this pathogen in China. This species has previously been shown to infect inoculated *Eucalyptus* seedlings [10] and could pose a threat to *Eucalyptus* plantation forestry. *Calonectria pacifica* was isolated from soils both in the *Eucalyptus* plantations (Minhou and Yongan Counties) and natural forests (Yanping District) in this study. This species was originally described on *Araucaria heterophylla* from Hawaii, USA [40], and this is the first report of the fungus in China.

This study elucidated the diversity and distribution characteristics of *Calonectria* species in soils collected from plantations and natural forests in Fujian Province. Broad patterns of occurrence were clear with *Eucalyptus* soils yielding the largest number of species. The conifer forests had the lowest number of species, which is consistent with the fact that most *Calonectria* spp. are known from Angiosperm hosts or from soils associated with these plants. The results of the present study bring the number of *Calonectria* species recorded in Fujian to 11. Most of these species have also been shown to be pathogenic to *Eucalyptus* in previous studies [7,9,10]. The surprisingly high species diversity in this region suggests that *Calonectria* species will pose long-term challenges for the development of *Eucalyptus* forestry in southern China.

## Figures and Tables

**Table 1:** Details of soils sampled, associated forest types and *Calonectria* spp. isolated.

Code	Sampling Site	Substrate	Number of Samples	Number of Samples Obtained *Calonectria*	Number of *alonectria* Isolates Obtained	*Calonectria* spp. (Number of Isolates)
a	Yanping District	*Eucalyptus* plantation	5	3	12	*Ca. aconidialis* (7); *Ca. pseudoreteaudii* (5)
		natural forest	13	3	10	*Ca. aconidialis* (3); *Ca*. *pacifica* (7)
		*Cunninghamia lanceolata*	10	0	0	N/A ^a^
b	Minhou County	*Eucalyptus* plantation	15	12	51	*Ca. aconidialis* (28); *Ca*. *kyotensis* (17); *Ca. hongkongensis* (4); *Ca*. *pacifica* (2)
c	Cangshan District	natural forest	3	3	11	*Ca. kyotensis* (5); *Ca*. *hongkongensis* (6)
d	Qingliu County	natural forest	10	0	0	N/A
		*Cunninghamia lanceolata*	11	2	9	*Ca. aconidialis* (5); *Ca*. *kyotensis* (4)
		*Pinus massoniana*	10	1	5	*Ca. kyotensis* (5)
e	Yongan County	*Eucalyptus* plantation	27	7	28	*Ca. aconidialis* (12); *Ca*. *kyotensis* (8); *Ca. pacifica* (8)
f	Liancheng County	*Eucalyptus* plantation	20	4	20	*Ca. kyotensis* (20)
		natural forest	17	8	40	*Ca. aconidialis* (27); *Ca*. *kyotensis* (8); *Ca. minensis* (5)
g	Zhangping County	*Eucalyptus* plantation	20	15	71	*Ca. aconidialis* (66); *Ca*. *ilicicola* (5)
h	Xinluo District	*Eucalyptus* plantation	19	4	16	*Ca. aconidialis* (6); *Ca*. *kyotensis* (7); *Ca. minensis* (3)
		*Phyllostachys heterocycle*	14	5	25	*Ca. aconidialis* (5); *Ca*. *kyotensis* (20)
i	Hua’an County	*Eucalyptus* plantation	15	12	55	*Ca. aconidialis* (19); *Ca*. *kyotensis* (9); *Ca. hongkongensis* (27)
		In total	209	79	353	

^a^ N/A refers to samples that did not yield *Calonectria* isolates.

**Table 2:** Isolates sequenced in this study and used for phylogenetic analyses and morphological studies.

Species ^a^	Isolate No. ^b,c^	Genotype ^d^	Substrate	Sampling Site	GPS Coordinate	Collector	GenBank Accession No. ^e^					
							*act*	*cmdA*	*his3*	*rpb2*	*tef1*	*tub2*
*Calonectria* *aconidialis*	CSF9779	AAA-AA	Soil (*Eucalyptus* plantation)	Hua’an, Zhangzhou, Fujian, China	24°53′49.369″ N, 117°32′45.070″ E	S.F. Chen, Q.L. Liu and F.F. Liu	OK253064	OK253135	OK253279	N/A ^f^	OK253491	OK253844
	CSF9857	AAAAAA	Soil (*Eucalyptus* plantation)	Zhangping, Longyan, Fujian, China	25°17′10.882″ N, 117°27′33.635″ E	S.F. Chen, Q.L. Liu and F.F. Liu	OK253065	OK253136	OK253280	OK253423	OK253492	OK253845
	CSF9937	AAAABA	Soil (*Eucalyptus* plantation)	Xinluo, Longyan, Fujian, China	25°07′08.597″ N, 116°44′42.257″ E	S.F. Chen, Q.L. Liu and F.F. Liu	OK253066	OK253137	OK253281	OK253424	OK253493	OK253846
	CSF9938	AAAABA	Soil (*Eucalyptus* plantation)	Xinluo, Longyan, Fujian, China	25°07′08.597″ N, 116°44′42.257″ E	S.F. Chen, Q.L. Liu and F.F. Liu	OK253067	OK253138	OK253282	OK253425	OK253494	OK253847
	CSF9939	AAAABA	Soil (*Eucalyptus* plantation)	Xinluo, Longyan, Fujian, China	25°07′08.597″ N, 116°44′42.257″ E	S.F. Chen, Q.L. Liu and F.F. Liu	OK253068	OK253139	OK253283	OK253426	OK253495	OK253848
	CSF9809	ABAAAA	Soil (*Eucalyptus* plantation)	Hua’an, Zhangzhou, Fujian, China	24°53′49.369″ N, 117°32′45.070″ E	S.F. Chen, Q.L. Liu and F.F. Liu	OK253069	OK253140	OK253284	OK253427	OK253496	OK253849
	CSF10105	ABAAAA	Soil (*Eucalyptus* plantation)	Minhou, Fuzhou, Fujian, China	26°15′04.285″ N, 119°02′38.917″ E	S.F. Chen, Q.L. Liu and F.F. Liu	OK253070	OK253141	OK253285	OK253428	OK253497	OK253850
	CSF9789	ABAAAB	Soil (*Eucalyptus* plantation)	Hua’an, Zhangzhou, Fujian, China	24°53′49.369″ N, 117°32′45.070″ E	S.F. Chen, Q.L. Liu and F.F. Liu	OK253071	OK253142	OK253286	OK253429	OK253498	OK253851
	CSF9839	ABAAAC	Soil (*Eucalyptus* plantation)	Zhangping, Longyan, Fujian, China	25°17′10.882″ N, 117°27′33.635″ E	S.F. Chen, Q.L. Liu and F.F. Liu	OK253072	OK253143	OK253287	OK253430	OK253499	OK253852
	CSF9844	ABAAAC	Soil (*Eucalyptus* plantation)	Zhangping, Longyan, Fujian, China	25°17′10.882″ N, 117°27′33.635″ E	S.F. Chen, Q.L. Liu and F.F. Liu	OK253073	OK253144	OK253288	OK253431	OK253500	OK253853
	CSF9882	ABAAAD	Soil (*Eucalyptus* plantation)	Zhangping, Longyan, Fujian, China	25°17′10.882″ N, 117°27′33.635″ E	S.F. Chen, Q.L. Liu and F.F. Liu	OK253074	OK253145	OK253289	OK253432	OK253501	OK253854
	CSF9987	ABAAAD	Soil (natural forest area)	Liancheng, Longyan, Fujian, China	25°26′14.348″ N, 116°38′42.400″ E	S.F. Chen, Q.L. Liu and F.F. Liu	OK253075	OK253146	OK253290	OK253433	OK253502	OK253855
	CSF9813	ABAACA	Soil (*Eucalyptus* plantation)	Hua’an, Zhangzhou, Fujian, China	24°53′49.369″ N, 117°32′45.070″ E	S.F. Chen, Q.L. Liu and F.F. Liu	OK253076	OK253147	OK253291	OK253434	OK253503	OK253856
	CSF9841	ABAACA	Soil (*Eucalyptus* plantation)	Zhangping, Longyan, Fujian, China	25°17′10.882″ N, 117°27′33.635″ E	S.F. Chen, Q.L. Liu and F.F. Liu	OK253077	OK253148	OK253292	OK253435	OK253504	OK253857
	CSF9870	ABBAAA	Soil (*Eucalyptus* plantation)	Zhangping, Longyan, Fujian, China	25°17′10.882″ N, 117°27′33.635″ E	S.F. Chen, Q.L. Liu and F.F. Liu	OK253078	OK253149	OK253293	OK253436	OK253505	OK253858
	CSF9875	ABB-AA	Soil (*Eucalyptus* plantation)	Zhangping, Longyan, Fujian, China	25°17′10.882″ N, 117°27′33.635″ E	S.F. Chen, Q.L. Liu and F.F. Liu	OK253079	OK253150	OK253294	N/A	OK253506	OK253859
	CSF9957	ACBAAA	Soil (natural forest area)	Liancheng, Longyan, Fujian, China	25°26′14.348″ N, 116°38′42.400″ E	S.F. Chen, Q.L. Liu and F.F. Liu	OK253080	OK253151	OK253295	OK253437	OK253507	OK253860
*Ca.* *hongkongensis*	CSF7124	AAAAAA	Soil (natural forest area)	Cangshan, Fuzhou, Fujian, China	26°5′16.2″ N, 119°14′19.8″ E	S.F. Chen, Q.L. Liu and F.F. Liu	OK253081	OK253192	OK253336	OK253438	OK253669	OK253900
	CSF9784	AAAAAA	Soil (*Eucalyptus* plantation)	Hua’an, Zhangzhou, Fujian, China	24°53′49.369″ N, 117°32′45.070″ E	S.F. Chen, Q.L. Liu and F.F. Liu	OK253082	OK253193	OK253337	OK253439	OK253670	OK253901
	CSF9794	ABAAAA	Soil (*Eucalyptus* plantation)	Hua’an, Zhangzhou, Fujian, China	24°53′49.369″ N, 117°32′45.070″ E	S.F. Chen, Q.L. Liu and F.F. Liu	OK253083	OK253194	OK253338	OK253440	OK253671	OK253902
	CSF9799	ABAAAA	Soil (*Eucalyptus* plantation)	Hua’an, Zhangzhou, Fujian, China	24°53′49.369″ N, 117°32′45.070″ E	S.F. Chen, Q.L. Liu and F.F. Liu	OK253084	OK253195	OK253339	OK253441	OK253672	OK253903
*Ca. ilicicola*	CSF9862	AAAAAA	Soil (*Eucalyptus* plantation)	Zhangping, Longyan, Fujian, China	25°17′10.882″ N, 117°27′33.635″ E	S.F. Chen, Q.L. Liu and F.F. Liu	OK253085	OK253202	OK253346	OK253442	OK253706	OK253910
	CSF9863	AAAAAA	Soil (*Eucalyptus* plantation)	Zhangping, Longyan, Fujian, China	25°17′10.882″ N, 117°27′33.635″ E	S.F. Chen, Q.L. Liu and F.F. Liu	OK253086	OK253203	OK253347	OK253443	OK253707	OK253911
*Ca. kyotensis*	CSF7130	AAAAAA	Soil (natural forest area)	Cangshan, Fuzhou, Fujian, China	26°5′16.2″ N, 119°14′19.8″ E	S.F. Chen, Q.L. Liu and F.F. Liu	OK253087	OK253207	OK253351	OK253444	OK253711	OK253915
	CSF10088	AAAAAA	Soil (*Eucalyptus* plantation)	Minhou, Fuzhou, Fujian, China	26°15′04.285″ N, 119°02′38.917″ E	S.F. Chen, Q.L. Liu and F.F. Liu	OK253088	OK253208	OK253352	OK253445	OK253712	OK253916
	CSF9834	AAA-AB	Soil (*Eucalyptus* plantation)	Hua’an, Zhangzhou, Fujian, China	24°58′22.263″ N, 117°31′09.708″ E	S.F. Chen, Q.L. Liu and F.F. Liu	OK253089	OK253209	OK253353	N/A	OK253713	OK253917
	CSF9910	AAAAAB	Soil (*Phyllostachys* *heterocycla*)	Xinluo, Longyan, Fujian, China	25°07′31.133″ N, 116°51′37.485″ E	S.F. Chen, Q.L. Liu and F.F. Liu	OK253090	OK253210	OK253354	OK253446	OK253714	OK253918
	CSF10014	AAAAAC	Soil (*Eucalyptus* plantation)	Yongan, Sanming, Fujian, China	25°55′10.860″ N, 117°16′39.591″ E	S.F. Chen, Q.L. Liu and F.F. Liu	OK253091	OK253211	OK253355	OK253447	OK253715	OK253919
	CSF10080	AAAAAD	Soil (*Eucalyptus* plantation)	Minhou, Fuzhou, Fujian, China	26°15′04.285″ N, 119°02′38.917″ E	S.F. Chen, Q.L. Liu and F.F. Liu	OK253092	OK253212	OK253356	OK253448	OK253716	OK253920
	CSF10086	AAAAAE	Soil (*Eucalyptus* plantation)	Minhou, Fuzhou, Fujian, China	26°15′04.285″ N, 119°02′38.917″ E	S.F. Chen, Q.L. Liu and F.F. Liu	OK253093	OK253213	OK253357	OK253449	OK253717	OK253921
	CSF10053	AAAABB	Soil (*Pinus massoniana*)	Qingliu, Sanming, Fujian, China	26°10′54.311″ N, 116°52′50.901″ E	S.F. Chen, Q.L. Liu and F.F. Liu	OK253094	OK253214	OK253358	OK253450	OK253718	OK253922
	CSF10054	AAAABB	Soil (*Pinus massoniana*)	Qingliu, Sanming, Fujian, China	26°10′54.311″ N, 116°52′50.901″ E	S.F. Chen, Q.L. Liu and F.F. Liu	OK253095	OK253215	OK253359	OK253451	OK253719	OK253923
	CSF9922	AAAABF	Soil (*Phyllostachys* *heterocycla*)	Xinluo, Longyan, Fujian, China	25°07′31.133″ N, 116°51′37.485″ E	S.F. Chen, Q.L. Liu and F.F. Liu	OK253096	OK253216	OK253360	OK253452	OK253720	OK253924
	CSF9923	AAAABF	Soil (*Phyllostachys* *heterocycla*)	Xinluo, Longyan, Fujian, China	25°07′31.133″ N, 116°51′37.485″ E	S.F. Chen, Q.L. Liu and F.F. Liu	OK253097	OK253217	OK253361	OK253453	OK253721	OK253925
	CSF9949	AAAADB	Soil (*Eucalyptus* plantation)	Xinluo, Longyan, Fujian, China	25°07′08.597″ N, 116°44′42.257″ E	S.F. Chen, Q.L. Liu and F.F. Liu	OK253098	OK253218	OK253362	OK253454	OK253722	OK253926
	CSF9951	AAAADB	Soil (*Eucalyptus* plantation)	Xinluo, Longyan, Fujian, China	25°07′08.597″ N, 116°44′42.257″ E	S.F. Chen, Q.L. Liu and F.F. Liu	OK253099	OK253219	OK253363	OK253455	OK253723	OK253927
	CSF9932	AAAADG	Soil (*Eucalyptus* plantation)	Xinluo, Longyan, Fujian, China	25°07′08.597″ N, 116°44′42.257″ E	S.F. Chen, Q.L. Liu and F.F. Liu	OK253100	OK253220	OK253364	OK253456	OK253724	OK253928
	CSF9935	AAAADG	Soil (*Eucalyptus* plantation)	Xinluo, Longyan, Fujian, China	25°07′08.597″ N, 116°44′42.257″ E	S.F. Chen, Q.L. Liu and F.F. Liu	OK253101	OK253221	OK253365	OK253457	OK253725	OK253929
	CSF9936	AAAADG	Soil (*Eucalyptus* plantation)	Xinluo, Longyan, Fujian, China	25°07′08.597″ N, 116°44′42.257″ E	S.F. Chen, Q.L. Liu and F.F. Liu	OK253102	OK253222	OK253366	OK253458	OK253726	OK253930
	CSF10020	AAAAEA	Soil (*Eucalyptus* plantation)	Yongan, Sanming, Fujian, China	25°55′10.860″ N, 117°16′39.591″ E	S.F. Chen, Q.L. Liu and F.F. Liu	OK253103	OK253223	OK253367	OK253459	OK253727	OK253931
	CSF10021	AAAAEA	Soil (*Eucalyptus* plantation)	Yongan, Sanming, Fujian, China	25°55′10.860″ N, 117°16′39.591″ E	S.F. Chen, Q.L. Liu and F.F. Liu	OK253104	OK253224	OK253368	OK253460	OK253728	OK253932
	CSF10009	AAABBH	Soil (*Eucalyptus* plantation)	Liancheng, Longyan, Fujian, China	25°33′06.994″ N, 116°41′42.328″ E	S.F. Chen, Q.L. Liu and F.F. Liu	OK253105	OK253225	OK253369	OK253461	OK253729	OK253933
	CSF10010	AAABBH	Soil (*Eucalyptus* plantation)	Liancheng, Longyan, Fujian, China	25°33′06.994″ N, 116°41′42.328″ E	S.F. Chen, Q.L. Liu and F.F. Liu	OK253106	OK253226	OK253370	OK253462	OK253730	OK253934
	CSF9997	AABAAB	Soil (*Eucalyptus* plantation)	Liancheng, Longyan, Fujian, China	25°33′06.994″ N, 116°41′42.328″ E	S.F. Chen, Q.L. Liu and F.F. Liu	OK253107	OK253227	OK253371	OK253463	OK253731	OK253935
	CSF9969	AABACB	Soil (natural forest area)	Liancheng, Longyan, Fujian, China	25°26′14.348″ N, 116°38′42.400″ E	S.F. Chen, Q.L. Liu and F.F. Liu	OK253108	OK253228	OK253372	OK253464	OK253732	OK253936
	CSF9972	AABACB	Soil (natural forest area)	Liancheng, Longyan, Fujian, China	25°26′14.348″ N, 116°38′42.400″ E	S.F. Chen, Q.L. Liu and F.F. Liu	OK253109	OK253229	OK253373	OK253465	OK253733	OK253937
	CSF9973	AABACB	Soil (natural forest area)	Liancheng, Longyan, Fujian, China	25°26′14.348″ N, 116°38′42.400″ E	S.F. Chen, Q.L. Liu and F.F. Liu	OK253110	OK253230	OK253374	OK253466	OK253734	OK253938
	CSF10126	AACAAA	Soil (*Eucalyptus* plantation)	Minhou, Fuzhou, Fujian, China	26°15′04.285″ N, 119°02′38.917″ E	S.F. Chen, Q.L. Liu and F.F. Liu	OK253111	OK253231	OK253375	OK253467	OK253735	OK253939
	CSF9962	AACAAD	Soil (natural forest area)	Liancheng, Longyan, Fujian, China	25°26′14.348″ N, 116°38′42.400″ E	S.F. Chen, Q.L. Liu and F.F. Liu	OK253112	OK253232	OK253376	OK253468	OK253736	OK253940
	CSF10019	AADABB	Soil (*Eucalyptus* plantation)	Yongan, Sanming, Fujian, China	25°55′10.860″ N, 117°16′39.591″ E	S.F. Chen, Q.L. Liu and F.F. Liu	OK253113	OK253233	OK253377	OK253469	OK253737	OK253941
	CSF10022	AADABB	Soil (*Eucalyptus* plantation)	Yongan, Sanming, Fujian, China	25°55′10.860″ N, 117°16′39.591″ E	S.F. Chen, Q.L. Liu and F.F. Liu	OK253114	OK253234	OK253378	OK253470	OK253738	OK253942
	CSF10023	AADABB	Soil (*Eucalyptus* plantation)	Yongan, Sanming, Fujian, China	25°55′10.860″ N, 117°16′39.591″ E	S.F. Chen, Q.L. Liu and F.F. Liu	OK253115	OK253235	OK253379	OK253471	OK253739	OK253943
	CSF10045	ABAAAB	Soil (*Cunninghamia* *lanceolata*)	Qingliu, Sanming, Fujian, China	26°07′23.497″ N, 116°53′00.762″ E	S.F. Chen, Q.L. Liu and F.F. Liu	OK253116	OK253236	OK253380	OK253472	OK253740	OK253944
	CSF10047	ABAAAB	Soil (*Cunninghamia* *lanceolata*)	Qingliu, Sanming, Fujian, China	26°07′23.497″ N, 116°53′00.762″ E	S.F. Chen, Q.L. Liu and F.F. Liu	OK253117	OK253237	OK253381	OK253473	OK253741	OK253945
	CSF9824	ACBAAC	Soil (*Eucalyptus* plantation)	Hua’an, Zhangzhou, Fujian, China	24°53′49.369″ N, 117°32′45.070″ E	S.F. Chen, Q.L. Liu and F.F. Liu	OK253118	OK253238	OK253382	OK253474	OK253742	OK253946
	CSF10004	ADAACB	Soil (*Eucalyptus* plantation)	Liancheng, Longyan, Fujian, China	25°33′06.994″ N, 116°41′42.328″ E	S.F. Chen, Q.L. Liu and F.F. Liu	OK253119	OK253239	OK253383	OK253475	OK253743	OK253947
	CSF10005	ADAACB	Soil (*Eucalyptus* plantation)	Liancheng, Longyan, Fujian, China	25°33′06.994″ N, 116°41′42.328″ E	S.F. Chen, Q.L. Liu and F.F. Liu	OK253120	OK253240	OK253384	OK253476	OK253744	OK253948
*Ca. minensis* *sp. nov.*	CSF9941 ^g–i^; CGMCC3.18877	AAAAAA	Soil (*Eucalyptus* plantation)	Xinluo, Longyan, Fujian, China	25°07′08.597″ N, 116°44′42.257″ E	S.F. Chen, Q.L. Liu and F.F. Liu	OK253121	OK253259	OK253403	OK253477	OK253814	OK253967
	CSF9974	AAAAAA	Soil (natural forest area)	Liancheng, Longyan, Fujian, China	25°26′14.348″ N, 116°38′42.400″ E	S.F. Chen, Q.L. Liu and F.F. Liu	OK253122	OK253260	OK253404	OK253478	OK253815	OK253968
	CSF9975 ^g,h^; CGMCC3.18881	AAAAAA	Soil (natural forest area)	Liancheng, Longyan, Fujian, China	25°26′14.348″ N, 116°38′42.400″ E	S.F. Chen, Q.L. Liu and F.F. Liu	OK253123	OK253261	OK253405	OK253479	OK253816	OK253969
	CSF9976	AAAAAA	Soil (natural forest area)	Liancheng, Longyan, Fujian, China	25°26′14.348″ N, 116°38′42.400″ E	S.F. Chen, Q.L. Liu and F.F. Liu	OK253124	OK253262	OK253406	OK253480	OK253817	OK253970
	CSF9977	AAAAAA	Soil (natural forest area)	Liancheng, Longyan, Fujian, China	25°26′14.348″ N, 116°38′42.400″ E	S.F. Chen, Q.L. Liu and F.F. Liu	OK253125	OK253263	OK253407	OK253481	OK253818	OK253971
	CSF9978	AAAAAA	Soil (natural forest area)	Liancheng, Longyan, Fujian, China	25°26′14.348″ N, 116°38′42.400″ E	S.F. Chen, Q.L. Liu and F.F. Liu	OK253126	OK253264	OK253408	OK253482	OK253819	OK253972
	CSF9933 ^g,h^; CGMCC3.18875	ABBABB	Soil (*Eucalyptus* plantation)	Xinluo, Longyan, Fujian, China	25°07′08.597″ N, 116°44′42.257″ E	S.F. Chen, Q.L. Liu and F.F. Liu	OK253127	OK253265	OK253409	OK253483	OK253820	OK253973
	CSF9934	ABBABB	Soil (*Eucalyptus* plantation)	Xinluo, Longyan, Fujian, China	25°07′08.597″ N, 116°44′42.257″ E	S.F. Chen, Q.L. Liu and F.F. Liu	OK253128	OK253266	OK253410	OK253484	OK253821	OK253974
*Ca. pacifica*	CSF10024	AAAAAA	Soil (*Eucalyptus* plantation)	Yongan, Sanming, Fujian, China	25°55′10.860″ N, 117°16′39.591″ E	S.F. Chen, Q.L. Liu and F.F. Liu	OK253129	OK253267	OK253411	OK253485	OK253822	OK253975
	CSF10129	BAAAAA	Soil (*Eucalyptus* plantation)	Minhou, Fuzhou, Fujian, China	26°15′04.285″ N, 119°02′38.917″ E	S.F. Chen, Q.L. Liu and F.F. Liu	OK253130	OK253268	OK253412	OK253486	OK253823	OK253976
	CSF10070	CABAAA	Soil (natural forest area)	Yanping, Nanping, Fujian, China	26°42′26.672″ N, 118°07′58.317″ E	S.F. Chen, Q.L. Liu and F.F. Liu	OK253131	OK253269	OK253413	OK253487	OK253824	OK253977
	CSF10077	CABAAA	Soil (natural forest area)	Yanping, Nanping, Fujian, China	26°42′26.672″ N, 118°07′58.317″ E	S.F. Chen, Q.L. Liu and F.F. Liu	OK253132	OK253270	OK253414	OK253488	OK253825	OK253978
*Ca.* *pseudoreteaudii*	CSF10059	AAAAAA	Soil (*Eucalyptus* plantation)	Yanping, Nanping, Fujian, China	26°46′19.651″ N, 117°57′37.233″ E	S.F. Chen, Q.L. Liu and F.F. Liu	OK253133	OK253274	OK253418	OK253489	OK253839	OK253982
	CSF10060 ^g,h^	AAAAAA	Soil (*Eucalyptus* plantation)	Yanping, Nanping, Fujian, China	26°46′19.651″ N, 117°57′37.233″ E	S.F. Chen, Q.L. Liu and F.F. Liu	OK253134	OK253275	OK253419	OK253490	OK253840	OK253983

^a^ New species described in this study are indicated in bold. ^b^ *CSF* = Culture Collection from Southern Forests (CSF), ZhanJiang, Guangdong Province, China; *CGMCC* = China General Microbiological Culture Collection Center, Beijing, China. ^c^ Isolates used in phylogenetic analyses. ^d^ Genotype within each identified species, determined by sequences of *act*, *cmdA*, *his3*, *rpb2*, *tef1* and *tub2* regions; ‘-’ means not available. ^e^ *act* = actin; *cmdA* = calmodulin; *his3* = histone H3; *rpb2* = the second largest subunit of RNA polymerase; *tef1* = translation elongation factor 1-alpha; *tub2 =* β-tubulin. ^f^ *N/A* represents sequences that are not available. ^g^ Isolates used in morphological and culture growth studies. ^h^ Isolates used for mating studies. ^i^ Isolates that represent ex-type cultures are indicated in bold.

**Table 4:** Statistics resulting from phylogenetic analyses in this study.

Dataset	No. of Taxa	No. of bp ^a^	Maximum Parsimony						
			PIC ^b^	No. of Trees	Tree Length	CI ^c^	RI ^d^	RC ^e^	HI ^f^
*act*	147	278	111	4	258	0.636	0.968	0.615	0.364
*cmdA*	147	672	291	433	677	0.647	0.968	0.626	0.353
*his3*	143	464	183	1000	830	0.475	0.928	0.440	0.525
*rpb2*	134	863	269	1000	683	0.530	0.959	0.508	0.470
*tef1*	149	532	267	1000	758	0.637	0.963	0.613	0.363
*tub2*	135	597	286	1000	826	0.609	0.958	0.584	0.391
*act/cmdA/his3/rpb2/tef1/tub2*	149	3406	1407	3000	4408	0.532	0.949	0.504	0.468
**Dataset**	**Maximum Likelihood**								
	**Subst. Mode ^g^**	**NST ^h^**	**Rate Matrix**					**Rates**	
*act*	TPM2 + G	6	0.5990	4.0516	0.5990	1.0000	4.0516	Gamma	
*cmdA*	TrN + G	6	1.0000	4.1556	1.0000	1.0000	7.1231	Gamma	
*his3*	TPM2uf + I + G	6	1.2442	6.0957	1.2442	1.0000	6.0957	Gamma	
*rpb2*	TrNef + I + G	6	1.0000	9.0443	1.0000	1.0000	13.4319	Gamma	
*tef1*	GTR + G	6	0.9651	1.7160	1.1302	0.5271	3.1484	Gamma	
*tub2*	TPM3uf + I + G	6	1.4044	4.4908	1.0000	1.4044	4.4908	Gamma	
*act/cmdA/his3/rpb2/tef1/tub2*	GTR + I + G	6	1.4593	4.5939	1.1370	0.9972	6.3874	Gamma	

^a^ *bp* = Base pairs. ^b^ *PIC* = Number of parsimony informative characters. ^c^ *CI* = Consistency index. ^d^ *RI* = Retention index. ^e^ *RC* = Rescaled consistency index. ^f^ *HI* = Homoplasy index. ^g^ *Subst. model* = best fit substitution model. ^h^ *NST* = Number of substitution rate categories.

**Table 5:** Morphological comparisons of *Calonectria* species obtained in this study and other phylogenetically closely related species.

Species	References or Source of Data	Ascospores (L × W) ^a,b,c^	Ascospores Average (L × W) ^a,b^	Ascospores Septation	Macroconidia (L × W) ^a,b,c^	Macroconidia Average (L × W) ^a,b^	Macroconidia Septation	Vesicle (Min.–Max.) ^a^
*Calonectria minensis*	this study	(38.5–)46.5–64.5(–80.5) × (6–)6.5–8(–8.5)	55.5 × 7	3	(51–)55–66(–79) × (4.5–)5–6(–7.5)	60.5 × 5.5	(1–)3	3–5
*Ca. aciculata*	[4]	N/A ^d^	N/A	N/A	(53–)62–76(–86) × (4.5–)5–6(–7)6	69 × 5.5	3	(2–)2.5–3.5(–5)
*Ca. colhounii*	[17]	(30–)50–65(–75) × (4–)5–6(–8)	55 × 6	(1–)3	(45–)60–70(–80) × (4–)5–(–6)	65 × 5	(1–)3	3–4
*Ca. eucalypti*	[37]	(25–)30–36(–56) × (3–)5–6(–8)	33 × 6	(1–)3	(66–)69–75(–80) × (5–)6	72 × 6	3	4–6
*Ca. honghensis*	[4]	(35–)43–55(–65) × (4.5–)5.5–6.5(–7.5)	49 × 6	3	(43–)49–59(–66) × (4.5–)5–5.5(–6)	54 × 5.5	3	(2.5–)3–4.5(–5.5)

^a^ All measurements are in µm. ^b^ L × W = length × width. ^c^ Measurements are presented in the format [(minimum–) (average–standard deviation)–(average + standard deviation) (–maximum)]. ^d^ N/A represents data that is not available.

## Data Availability

The sequences from the current study were submitted to the NCBI database (https://www.ncbi.nlm.nih.gov/, accessed on 24 July 2022) and the accession numbers were listed in Table 2.

## Appendix A. Species of *Calonectria* Collected in This Study

**Table A1:** Species of *Calonectria* collected in this study.

Species ^a^	Isolate No. ^b^	Genotype ^c^	Substrate	Sampling Site	GPS Coordinate	Collector	GenBank Accession No. ^d^					
							*act*	*cmdA*	*his3*	*rpb2*	*tef1*	*tub2*
*Calonectria* *aconidialis*	CSF9779 ^e^	AAA-AA	Soil (*Eucalyptus* plantation)	Hua’an, Zhangzhou, Fujian, China	24°53′49.369″ N, 117°32′45.070″ E	S.F. Chen, Q.L. Liu and F.F. Liu	OK253064	OK253135	OK253279	N/A ^f^	OK253491	OK253844
	CSF9857 ^e^	AAAAAA	Soil (*Eucalyptus* plantation)	Zhangping, Longyan, Fujian, China	25°17′10.882″ N, 117°27′33.635″ E	S.F. Chen, Q.L. Liu and F.F. Liu	OK253065	OK253136	OK253280	OK253423	OK253492	OK253845
	CSF9937 ^e^	AAAABA	Soil (*Eucalyptus* plantation)	Xinluo, Longyan, Fujian, China	25°07′08.597″ N, 116°44′42.257″ E	S.F. Chen, Q.L. Liu and F.F. Liu	OK253066	OK253137	OK253281	OK253424	OK253493	OK253846
	CSF9938 ^e^	AAAABA	Soil (*Eucalyptus* plantation)	Xinluo, Longyan, Fujian, China	25°07′08.597″ N, 116°44′42.257″ E	S.F. Chen, Q.L. Liu and F.F. Liu	OK253067	OK253138	OK253282	OK253425	OK253494	OK253847
	CSF9939 ^e^	AAAABA	Soil (*Eucalyptus* plantation)	Xinluo, Longyan, Fujian, China	25°07′08.597″ N, 116°44′42.257″ E	S.F. Chen, Q.L. Liu and F.F. Liu	OK253068	OK253139	OK253283	OK253426	OK253495	OK253848
	CSF9809 ^e^	ABAAAA	Soil (*Eucalyptus* plantation)	Hua’an, Zhangzhou, Fujian, China	24°53′49.369″ N, 117°32′45.070″ E	S.F. Chen, Q.L. Liu and F.F. Liu	OK253069	OK253140	OK253284	OK253427	OK253496	OK253849
	CSF10105 ^e^	ABAAAA	Soil (*Eucalyptus* plantation)	Minhou, Fuzhou, Fujian, China	26°15′04.285″ N, 119°02′38.917″ E	S.F. Chen, Q.L. Liu and F.F. Liu	OK253070	OK253141	OK253285	OK253428	OK253497	OK253850
	CSF9789 ^e^	ABAAAB	Soil (*Eucalyptus* plantation)	Hua’an, Zhangzhou, Fujian, China	24°53′49.369″ N, 117°32′45.070″ E	S.F. Chen, Q.L. Liu and F.F. Liu	OK253071	OK253142	OK253286	OK253429	OK253498	OK253851
	CSF9839 ^e^	ABAAAC	Soil (*Eucalyptus* plantation)	Zhangping, Longyan, Fujian, China	25°17′10.882″ N, 117°27′33.635″ E	S.F. Chen, Q.L. Liu and F.F. Liu	OK253072	OK253143	OK253287	OK253430	OK253499	OK253852
	CSF9844 ^e^	ABAAAC	Soil (*Eucalyptus* plantation)	Zhangping, Longyan, Fujian, China	25°17′10.882″ N, 117°27′33.635″ E	S.F. Chen, Q.L. Liu and F.F. Liu	OK253073	OK253144	OK253288	OK253431	OK253500	OK253853
	CSF9882 ^e^	ABAAAD	Soil (*Eucalyptus* plantation)	Zhangping, Longyan, Fujian, China	25°17′10.882″ N, 117°27′33.635″ E	S.F. Chen, Q.L. Liu and F.F. Liu	OK253074	OK253145	OK253289	OK253432	OK253501	OK253854
	CSF9987 ^e^	ABAAAD	Soil (natural forest area)	Liancheng, Longyan, Fujian, China	25°26′14.348″ N, 116°38′42.400″ E	S.F. Chen, Q.L. Liu and F.F. Liu	OK253075	OK253146	OK253290	OK253433	OK253502	OK253855
	CSF9813 ^e^	ABAACA	Soil (*Eucalyptus* plantation)	Hua’an, Zhangzhou, Fujian, China	24°53′49.369″ N, 117°32′45.070″ E	S.F. Chen, Q.L. Liu and F.F. Liu	OK253076	OK253147	OK253291	OK253434	OK253503	OK253856
	CSF9841 ^e^	ABAACA	Soil (*Eucalyptus* plantation)	Zhangping, Longyan, Fujian, China	25°17′10.882″ N, 117°27′33.635″ E	S.F. Chen, Q.L. Liu and F.F. Liu	OK253077	OK253148	OK253292	OK253435	OK253504	OK253857
	CSF9870 ^e^	ABBAAA	Soil (*Eucalyptus* plantation)	Zhangping, Longyan, Fujian, China	25°17′10.882″ N, 117°27′33.635″ E	S.F. Chen, Q.L. Liu and F.F. Liu	OK253078	OK253149	OK253293	OK253436	OK253505	OK253858
	CSF9875 ^e^	ABB-AA	Soil (*Eucalyptus* plantation)	Zhangping, Longyan, Fujian, China	25°17′10.882″ N, 117°27′33.635″ E	S.F. Chen, Q.L. Liu and F.F. Liu	OK253079	OK253150	OK253294	N/A	OK253506	OK253859
	CSF9957 ^e^	ACBAAA	Soil (natural forest area)	Liancheng, Longyan, Fujian, China	25°26′14.348″ N, 116°38′42.400″ E	S.F. Chen, Q.L. Liu and F.F. Liu	OK253080	OK253151	OK253295	OK253437	OK253507	OK253860
	CSF9851	-AA-AA	Soil (*Eucalyptus* plantation)	Zhangping, Longyan, Fujian, China	25°17′10.882″ N, 117°27′33.635″ E	S.F. Chen, Q.L. Liu and F.F. Liu	– ^g^	OK253152	OK253296	–	OK253508	OK253861
	CSF9943	-AA-AA	Soil (*Eucalyptus* plantation)	Xinluo, Longyan, Fujian, China	25°07′08.597″ N, 116°44′42.257″ E	S.F. Chen, Q.L. Liu and F.F. Liu	–	OK253153	OK253297	–	OK253509	OK253862
	CSF9812	-BA-AA	Soil (*Eucalyptus* plantation)	Hua’an, Zhangzhou, Fujian, China	24°53′49.369″ N, 117°32′45.070″ E	S.F. Chen, Q.L. Liu and F.F. Liu	–	OK253154	OK253298	–	OK253510	OK253863
	CSF9831	-BA-AA	Soil (*Eucalyptus* plantation)	Hua’an, Zhangzhou, Fujian, China	24°58′22.263″ N, 117°31′09.708″ E	S.F. Chen, Q.L. Liu and F.F. Liu	–	OK253155	OK253299	–	OK253511	OK253864
	CSF9846	-BA-AA	Soil (*Eucalyptus* plantation)	Zhangping, Longyan, Fujian, China	25°17′10.882″ N, 117°27′33.635″ E	S.F. Chen, Q.L. Liu and F.F. Liu	–	OK253156	OK253300	–	OK253512	OK253865
	CSF9880	-BA-AA	Soil (*Eucalyptus* plantation)	Zhangping, Longyan, Fujian, China	25°17′10.882″ N, 117°27′33.635″ E	S.F. Chen, Q.L. Liu and F.F. Liu	–	OK253157	OK253301	–	OK253513	OK253866
	CSF9895	-BA-AA	Soil (*Eucalyptus* plantation)	Zhangping, Longyan, Fujian, China	25°17′10.882″ N, 117°27′33.635″ E	S.F. Chen, Q.L. Liu and F.F. Liu	–	OK253158	OK253302	–	OK253514	OK253867
	CSF9905	-BA-AA	Soil (*Eucalyptus* plantation)	Zhangping, Longyan, Fujian, China	25°17′10.882″ N, 117°27′33.635″ E	S.F. Chen, Q.L. Liu and F.F. Liu	–	OK253159	OK253303	–	OK253515	OK253868
	CSF9930	-BA-AA	Soil (*Phyllostachys heterocycla*)	Xinluo, Longyan, Fujian, China	25°07′31.133″ N, 116°51′37.485″ E	S.F. Chen, Q.L. Liu and F.F. Liu	–	OK253160	OK253304	–	OK253516	OK253869
	CSF9964	-BA-AA	Soil (natural forest area)	Liancheng, Longyan, Fujian, China	25°26′14.348″ N, 116°38′42.400″ E	S.F. Chen, Q.L. Liu and F.F. Liu	–	OK253161	OK253305	–	OK253517	OK253870
	CSF9970	-BA-AA	Soil (natural forest area)	Liancheng, Longyan, Fujian, China	25°26′14.348″ N, 116°38′42.400″ E	S.F. Chen, Q.L. Liu and F.F. Liu	–	OK253162	OK253306	–	OK253518	OK253871
	CSF9982	-BA-AA	Soil (natural forest area)	Liancheng, Longyan, Fujian, China	25°26′14.348″ N, 116°38′42.400″ E	S.F. Chen, Q.L. Liu and F.F. Liu	–	OK253163	OK253307	–	OK253519	OK253872
	CSF9989	-BA-AA	Soil (natural forest area)	Liancheng, Longyan, Fujian, China	25°26′14.348″ N, 116°38′42.400″ E	S.F. Chen, Q.L. Liu and F.F. Liu	–	OK253164	OK253308	–	OK253520	OK253873
	CSF10032	-BA-AA	Soil (*Eucalyptus* plantation)	Yongan, Sanming, Fujian, China	25°55′10.860″ N, 117°16′39.591″ E	S.F. Chen, Q.L. Liu and F.F. Liu	–	OK253165	OK253309	–	OK253521	OK253874
	CSF10034	-BA-AA	Soil (*Eucalyptus* plantation)	Yongan, Sanming, Fujian, China	25°55′10.860″ N, 117°16′39.591″ E	S.F. Chen, Q.L. Liu and F.F. Liu	–	OK253166	OK253310	–	OK253522	OK253875
	CSF10041	-BA-AA	Soil (*Eucalyptus* plantation)	Yongan, Sanming, Fujian, China	25°55′10.860″ N, 117°16′39.591″ E	S.F. Chen, Q.L. Liu and F.F. Liu	–	OK253167	OK253311	–	OK253523	OK253876
	CSF10050	-BA-AA	Soil (*Cunninghamia lanceolata*)	Qingliu, Sanming, Fujian, China	26°07′23.497″ N, 116°53′00.762″ E	S.F. Chen, Q.L. Liu and F.F. Liu	–	OK253168	OK253312	–	OK253524	OK253877
	CSF10064	-BA-AA	Soil (*Eucalyptus* plantation)	Yanping, Nanping, Fujian, China	26°46′19.651″ N, 117°57′37.233″ E	S.F. Chen, Q.L. Liu and F.F. Liu	–	OK253169	OK253313	–	OK253525	OK253878
	CSF10068	-BA-AA	Soil (*Eucalyptus* plantation)	Yanping, Nanping, Fujian, China	26°46′19.651″ N, 117°57′37.233″ E	S.F. Chen, Q.L. Liu and F.F. Liu	–	OK253170	OK253314	–	OK253526	OK253879
	CSF10073	-BA-AA	Soil (natural forest area)	Yanping, Nanping, Fujian, China	26°42′26.672″ N, 118°07′58.317″ E	S.F. Chen, Q.L. Liu and F.F. Liu	–	OK253171	OK253315	–	OK253527	OK253880
	CSF10075	-BA-AA	Soil (natural forest area)	Yanping, Nanping, Fujian, China	26°42′26.672″ N, 118°07′58.317″ E	S.F. Chen, Q.L. Liu and F.F. Liu	–	OK253172	OK253316	–	OK253528	OK253881
	CSF10081	-BA-AA	Soil (*Eucalyptus* plantation)	Minhou, Fuzhou, Fujian, China	26°15′04.285″ N, 119°02′38.917″ E	S.F. Chen, Q.L. Liu and F.F. Liu	–	OK253173	OK253317	–	OK253529	OK253882
	CSF10082	-BA-AA	Soil (*Eucalyptus* plantation)	Minhou, Fuzhou, Fujian, China	26°15′04.285″ N, 119°02′38.917″ E	S.F. Chen, Q.L. Liu and F.F. Liu	–	OK253174	OK253318	–	OK253530	OK253883
	CSF10097	-BA-AA	Soil (*Eucalyptus* plantation)	Minhou, Fuzhou, Fujian, China	26°15′04.285″ N, 119°02′38.917″ E	S.F. Chen, Q.L. Liu and F.F. Liu	–	OK253175	OK253319	–	OK253531	OK253884
	CSF10098	-BA-AA	Soil (*Eucalyptus* plantation)	Minhou, Fuzhou, Fujian, China	26°15′04.285″ N, 119°02′38.917″ E	S.F. Chen, Q.L. Liu and F.F. Liu	–	OK253176	OK253320	–	OK253532	OK253885
	CSF10110	-BA-A-	Soil (*Eucalyptus* plantation)	Minhou, Fuzhou, Fujian, China	26°15′04.285″ N, 119°02′38.917″ E	S.F. Chen, Q.L. Liu and F.F. Liu	–	OK253177	OK253321	–	OK253533	N/A
	CSF10112	-BA-AA	Soil (*Eucalyptus* plantation)	Minhou, Fuzhou, Fujian, China	26°15′04.285″ N, 119°02′38.917″ E	S.F. Chen, Q.L. Liu and F.F. Liu	–	OK253178	OK253322	–	OK253534	OK253886
	CSF10119	-BA-AA	Soil (*Eucalyptus* plantation)	Minhou, Fuzhou, Fujian, China	26°15′04.285″ N, 119°02′38.917″ E	S.F. Chen, Q.L. Liu and F.F. Liu	–	OK253179	OK253323	–	OK253535	OK253887
	CSF10125	-BA-AA	Soil (*Eucalyptus* plantation)	Minhou, Fuzhou, Fujian, China	26°15′04.285″ N, 119°02′38.917″ E	S.F. Chen, Q.L. Liu and F.F. Liu	–	OK253180	OK253324	–	OK253536	OK253888
	CSF9856	-BA-AC	Soil (*Eucalyptus* plantation)	Zhangping, Longyan, Fujian, China	25°17′10.882″ N, 117°27′33.635″ E	S.F. Chen, Q.L. Liu and F.F. Liu	–	OK253181	OK253325	–	OK253537	OK253889
	CSF9897	-BA-AC	Soil (*Eucalyptus* plantation)	Zhangping, Longyan, Fujian, China	25°17′10.882″ N, 117°27′33.635″ E	S.F. Chen, Q.L. Liu and F.F. Liu	–	OK253182	OK253326	–	OK253538	OK253890
	CSF9814	-BA-CA	Soil (*Eucalyptus* plantation)	Hua’an, Zhangzhou, Fujian, China	24°53′49.369″ N, 117°32′45.070″ E	S.F. Chen, Q.L. Liu and F.F. Liu	–	OK253183	OK253327	–	OK253539	OK253891
	CSF9815	-BA-CA	Soil (*Eucalyptus* plantation)	Hua’an, Zhangzhou, Fujian, China	24°53′49.369″ N, 117°32′45.070″ E	S.F. Chen, Q.L. Liu and F.F. Liu	–	OK253184	OK253328	–	OK253540	OK253892
	CSF9842	-BA-CA	Soil (*Eucalyptus* plantation)	Zhangping, Longyan, Fujian, China	25°17′10.882″ N, 117°27′33.635″ E	S.F. Chen, Q.L. Liu and F.F. Liu	–	OK253185	OK253329	–	OK253541	OK253893
	CSF9843	-BA-CA	Soil (*Eucalyptus* plantation)	Zhangping, Longyan, Fujian, China	25°17′10.882″ N, 117°27′33.635″ E	S.F. Chen, Q.L. Liu and F.F. Liu	–	OK253186	OK253330	–	OK253542	OK253894
	CSF9887	-BA-CA	Soil (*Eucalyptus* plantation)	Zhangping, Longyan, Fujian, China	25°17′10.882″ N, 117°27′33.635″ E	S.F. Chen, Q.L. Liu and F.F. Liu	–	OK253187	OK253331	–	OK253543	OK253895
	CSF9888	-BA-CA	Soil (*Eucalyptus* plantation)	Zhangping, Longyan, Fujian, China	25°17′10.882″ N, 117°27′33.635″ E	S.F. Chen, Q.L. Liu and F.F. Liu	–	OK253188	OK253332	–	OK253544	OK253896
	CSF9889	-BA-CA	Soil (*Eucalyptus* plantation)	Zhangping, Longyan, Fujian, China	25°17′10.882″ N, 117°27′33.635″ E	S.F. Chen, Q.L. Liu and F.F. Liu	–	OK253189	OK253333	–	OK253545	OK253897
	CSF9890	-BA-CA	Soil (*Eucalyptus* plantation)	Zhangping, Longyan, Fujian, China	25°17′10.882″ N, 117°27′33.635″ E	S.F. Chen, Q.L. Liu and F.F. Liu	–	OK253190	OK253334	–	OK253546	OK253898
	CSF9891	-BA-CA	Soil (*Eucalyptus* plantation)	Zhangping, Longyan, Fujian, China	25°17′10.882″ N, 117°27′33.635″ E	S.F. Chen, Q.L. Liu and F.F. Liu	–	OK253191	OK253335	–	OK253547	OK253899
	CSF9776	----A-	Soil (*Eucalyptus* plantation)	Hua’an, Zhangzhou, Fujian, China	24°53′49.369″ N, 117°32′45.070″ E	S.F. Chen, Q.L. Liu and F.F. Liu	–	–	–	–	OK253548	–
	CSF9777	----A-	Soil (*Eucalyptus* plantation)	Hua’an, Zhangzhou, Fujian, China	24°53′49.369″ N, 117°32′45.070″ E	S.F. Chen, Q.L. Liu and F.F. Liu	–	–	–	–	OK253549	–
	CSF9778	----A-	Soil (*Eucalyptus* plantation)	Hua’an, Zhangzhou, Fujian, China	24°53′49.369″ N, 117°32′45.070″ E	S.F. Chen, Q.L. Liu and F.F. Liu	–	–	–	–	OK253550	–
	CSF9780	----A-	Soil (*Eucalyptus* plantation)	Hua’an, Zhangzhou, Fujian, China	24°53′49.369″ N, 117°32′45.070″ E	S.F. Chen, Q.L. Liu and F.F. Liu	–	–	–	–	OK253551	–
	CSF9787	----A-	Soil (*Eucalyptus* plantation)	Hua’an, Zhangzhou, Fujian, China	24°53′49.369″ N, 117°32′45.070″ E	S.F. Chen, Q.L. Liu and F.F. Liu	–	–	–	–	OK253552	–
	CSF9788	----A-	Soil (*Eucalyptus* plantation)	Hua’an, Zhangzhou, Fujian, China	24°53′49.369″ N, 117°32′45.070″ E	S.F. Chen, Q.L. Liu and F.F. Liu	–	–	–	–	OK253553	–
	CSF9790	----A-	Soil (*Eucalyptus* plantation)	Hua’an, Zhangzhou, Fujian, China	24°53′49.369″ N, 117°32′45.070″ E	S.F. Chen, Q.L. Liu and F.F. Liu	–	–	–	–	OK253554	–
	CSF9806	----A-	Soil (*Eucalyptus* plantation)	Hua’an, Zhangzhou, Fujian, China	24°53′49.369″ N, 117°32′45.070″ E	S.F. Chen, Q.L. Liu and F.F. Liu	–	–	–	–	OK253555	–
	CSF9807	----A-	Soil (*Eucalyptus* plantation)	Hua’an, Zhangzhou, Fujian, China	24°53′49.369″ N, 117°32′45.070″ E	S.F. Chen, Q.L. Liu and F.F. Liu	–	–	–	–	OK253556	–
	CSF9808	----A-	Soil (*Eucalyptus* plantation)	Hua’an, Zhangzhou, Fujian, China	24°53′49.369″ N, 117°32′45.070″ E	S.F. Chen, Q.L. Liu and F.F. Liu	–	–	–	–	OK253557	–
	CSF9810	----A-	Soil (*Eucalyptus* plantation)	Hua’an, Zhangzhou, Fujian, China	24°53′49.369″ N, 117°32′45.070″ E	S.F. Chen, Q.L. Liu and F.F. Liu	–	–	–	–	OK253558	–
	CSF9836	----A-	Soil (*Eucalyptus* plantation)	Zhangping, Longyan, Fujian, China	25°17′10.882″ N, 117°27′33.635″ E	S.F. Chen, Q.L. Liu and F.F. Liu	–	–	–	–	OK253559	–
	CSF9837	----A-	Soil (*Eucalyptus* plantation)	Zhangping, Longyan, Fujian, China	25°17′10.882″ N, 117°27′33.635″ E	S.F. Chen, Q.L. Liu and F.F. Liu	–	–	–	–	OK253560	–
	CSF9838	----A-	Soil (*Eucalyptus* plantation)	Zhangping, Longyan, Fujian, China	25°17′10.882″ N, 117°27′33.635″ E	S.F. Chen, Q.L. Liu and F.F. Liu	–	–	–	–	OK253561	–
	CSF9840	----A-	Soil (*Eucalyptus* plantation)	Zhangping, Longyan, Fujian, China	25°17′10.882″ N, 117°27′33.635″ E	S.F. Chen, Q.L. Liu and F.F. Liu	–	–	–	–	OK253562	–
	CSF9845	----A-	Soil (*Eucalyptus* plantation)	Zhangping, Longyan, Fujian, China	25°17′10.882″ N, 117°27′33.635″ E	S.F. Chen, Q.L. Liu and F.F. Liu	–	–	–	–	OK253563	–
	CSF9847	----A-	Soil (*Eucalyptus* plantation)	Zhangping, Longyan, Fujian, China	25°17′10.882″ N, 117°27′33.635″ E	S.F. Chen, Q.L. Liu and F.F. Liu	–	–	–	–	OK253564	–
	CSF9848	----A-	Soil (*Eucalyptus* plantation)	Zhangping, Longyan, Fujian, China	25°17′10.882″ N, 117°27′33.635″ E	S.F. Chen, Q.L. Liu and F.F. Liu	–	–	–	–	OK253565	–
	CSF9849	----A-	Soil (*Eucalyptus* plantation)	Zhangping, Longyan, Fujian, China	25°17′10.882″ N, 117°27′33.635″ E	S.F. Chen, Q.L. Liu and F.F. Liu	–	–	–	–	OK253566	–
	CSF9850	----A-	Soil (*Eucalyptus* plantation)	Zhangping, Longyan, Fujian, China	25°17′10.882″ N, 117°27′33.635″ E	S.F. Chen, Q.L. Liu and F.F. Liu	–	–	–	–	OK253567	–
	CSF9852	----A-	Soil (*Eucalyptus* plantation)	Zhangping, Longyan, Fujian, China	25°17′10.882″ N, 117°27′33.635″ E	S.F. Chen, Q.L. Liu and F.F. Liu	–	–	–	–	OK253568	–
	CSF9853	----A-	Soil (*Eucalyptus* plantation)	Zhangping, Longyan, Fujian, China	25°17′10.882″ N, 117°27′33.635″ E	S.F. Chen, Q.L. Liu and F.F. Liu	–	–	–	–	OK253569	–
	CSF9854	----A-	Soil (*Eucalyptus* plantation)	Zhangping, Longyan, Fujian, China	25°17′10.882″ N, 117°27′33.635″ E	S.F. Chen, Q.L. Liu and F.F. Liu	–	–	–	–	OK253570	–
	CSF9855	----A-	Soil (*Eucalyptus* plantation)	Zhangping, Longyan, Fujian, China	25°17′10.882″ N, 117°27′33.635″ E	S.F. Chen, Q.L. Liu and F.F. Liu	–	–	–	–	OK253571	–
	CSF9858	----A-	Soil (*Eucalyptus* plantation)	Zhangping, Longyan, Fujian, China	25°17′10.882″ N, 117°27′33.635″ E	S.F. Chen, Q.L. Liu and F.F. Liu	–	–	–	–	OK253572	–
	CSF9859	----A-	Soil (*Eucalyptus* plantation)	Zhangping, Longyan, Fujian, China	25°17′10.882″ N, 117°27′33.635″ E	S.F. Chen, Q.L. Liu and F.F. Liu	–	–	–	–	OK253573	–
	CSF9860	----A-	Soil (*Eucalyptus* plantation)	Zhangping, Longyan, Fujian, China	25°17′10.882″ N, 117°27′33.635″ E	S.F. Chen, Q.L. Liu and F.F. Liu	–	–	–	–	OK253574	–
	CSF9861	----A-	Soil (*Eucalyptus* plantation)	Zhangping, Longyan, Fujian, China	25°17′10.882″ N, 117°27′33.635″ E	S.F. Chen, Q.L. Liu and F.F. Liu	–	–	–	–	OK253575	–
	CSF9867	----A-	Soil (*Eucalyptus* plantation)	Zhangping, Longyan, Fujian, China	25°17′10.882″ N, 117°27′33.635″ E	S.F. Chen, Q.L. Liu and F.F. Liu	–	–	–	–	OK253576	–
	CSF9868	----A-	Soil (*Eucalyptus* plantation)	Zhangping, Longyan, Fujian, China	25°17′10.882″ N, 117°27′33.635″ E	S.F. Chen, Q.L. Liu and F.F. Liu	–	–	–	–	OK253577	–
	CSF9869	----A-	Soil (*Eucalyptus* plantation)	Zhangping, Longyan, Fujian, China	25°17′10.882″ N, 117°27′33.635″ E	S.F. Chen, Q.L. Liu and F.F. Liu	–	–	–	–	OK253578	–
	CSF9871	----A-	Soil (*Eucalyptus* plantation)	Zhangping, Longyan, Fujian, China	25°17′10.882″ N, 117°27′33.635″ E	S.F. Chen, Q.L. Liu and F.F. Liu	–	–	–	–	OK253579	–
	CSF9872	----A-	Soil (*Eucalyptus* plantation)	Zhangping, Longyan, Fujian, China	25°17′10.882″ N, 117°27′33.635″ E	S.F. Chen, Q.L. Liu and F.F. Liu	–	–	–	–	OK253580	–
	CSF9873	----A-	Soil (*Eucalyptus* plantation)	Zhangping, Longyan, Fujian, China	25°17′10.882″ N, 117°27′33.635″ E	S.F. Chen, Q.L. Liu and F.F. Liu	–	–	–	–	OK253581	–
	CSF9874	----A-	Soil (*Eucalyptus* plantation)	Zhangping, Longyan, Fujian, China	25°17′10.882″ N, 117°27′33.635″ E	S.F. Chen, Q.L. Liu and F.F. Liu	–	–	–	–	OK253582	–
	CSF9876	----A-	Soil (*Eucalyptus* plantation)	Zhangping, Longyan, Fujian, China	25°17′10.882″ N, 117°27′33.635″ E	S.F. Chen, Q.L. Liu and F.F. Liu	–	–	–	–	OK253583	–
	CSF9877	----A-	Soil (*Eucalyptus* plantation)	Zhangping, Longyan, Fujian, China	25°17′10.882″ N, 117°27′33.635″ E	S.F. Chen, Q.L. Liu and F.F. Liu	–	–	–	–	OK253584	–
	CSF9878	----A-	Soil (*Eucalyptus* plantation)	Zhangping, Longyan, Fujian, China	25°17′10.882″ N, 117°27′33.635″ E	S.F. Chen, Q.L. Liu and F.F. Liu	–	–	–	–	OK253585	–
	CSF9879	----A-	Soil (*Eucalyptus* plantation)	Zhangping, Longyan, Fujian, China	25°17′10.882″ N, 117°27′33.635″ E	S.F. Chen, Q.L. Liu and F.F. Liu	–	–	–	–	OK253586	–
	CSF9881	----A-	Soil (*Eucalyptus* plantation)	Zhangping, Longyan, Fujian, China	25°17′10.882″ N, 117°27′33.635″ E	S.F. Chen, Q.L. Liu and F.F. Liu	–	–	–	–	OK253587	–
	CSF9883	----A-	Soil (*Eucalyptus* plantation)	Zhangping, Longyan, Fujian, China	25°17′10.882″ N, 117°27′33.635″ E	S.F. Chen, Q.L. Liu and F.F. Liu	–	–	–	–	OK253588	–
	CSF9884	----A-	Soil (*Eucalyptus* plantation)	Zhangping, Longyan, Fujian, China	25°17′10.882″ N, 117°27′33.635″ E	S.F. Chen, Q.L. Liu and F.F. Liu	–	–	–	–	OK253589	–
	CSF9885	----A-	Soil (*Eucalyptus* plantation)	Zhangping, Longyan, Fujian, China	25°17′10.882″ N, 117°27′33.635″ E	S.F. Chen, Q.L. Liu and F.F. Liu	–	–	–	–	OK253590	–
	CSF9886	----A-	Soil (*Eucalyptus* plantation)	Zhangping, Longyan, Fujian, China	25°17′10.882″ N, 117°27′33.635″ E	S.F. Chen, Q.L. Liu and F.F. Liu	–	–	–	–	OK253591	–
	CSF9892	----A-	Soil (*Eucalyptus* plantation)	Zhangping, Longyan, Fujian, China	25°17′10.882″ N, 117°27′33.635″ E	S.F. Chen, Q.L. Liu and F.F. Liu	–	–	–	–	OK253592	–
	CSF9893	----A-	Soil (*Eucalyptus* plantation)	Zhangping, Longyan, Fujian, China	25°17′10.882″ N, 117°27′33.635″ E	S.F. Chen, Q.L. Liu and F.F. Liu	–	–	–	–	OK253593	–
	CSF9894	----A-	Soil (*Eucalyptus* plantation)	Zhangping, Longyan, Fujian, China	25°17′10.882″ N, 117°27′33.635″ E	S.F. Chen, Q.L. Liu and F.F. Liu	–	–	–	–	OK253594	–
	CSF9896	----A-	Soil (*Eucalyptus* plantation)	Zhangping, Longyan, Fujian, China	25°17′10.882″ N, 117°27′33.635″ E	S.F. Chen, Q.L. Liu and F.F. Liu	–	–	–	–	OK253595	–
	CSF9898	----A-	Soil (*Eucalyptus* plantation)	Zhangping, Longyan, Fujian, China	25°17′10.882″ N, 117°27′33.635″ E	S.F. Chen, Q.L. Liu and F.F. Liu	–	–	–	–	OK253596	–
	CSF9899	----A-	Soil (*Eucalyptus* plantation)	Zhangping, Longyan, Fujian, China	25°17′10.882″ N, 117°27′33.635″ E	S.F. Chen, Q.L. Liu and F.F. Liu	–	–	–	–	OK253597	–
	CSF9900	----A-	Soil (*Eucalyptus* plantation)	Zhangping, Longyan, Fujian, China	25°17′10.882″ N, 117°27′33.635″ E	S.F. Chen, Q.L. Liu and F.F. Liu	–	–	–	–	OK253598	–
	CSF9901	----A-	Soil (*Eucalyptus* plantation)	Zhangping, Longyan, Fujian, China	25°17′10.882″ N, 117°27′33.635″ E	S.F. Chen, Q.L. Liu and F.F. Liu	–	–	–	–	OK253599	–
	CSF9902	----A-	Soil (*Eucalyptus* plantation)	Zhangping, Longyan, Fujian, China	25°17′10.882″ N, 117°27′33.635″ E	S.F. Chen, Q.L. Liu and F.F. Liu	–	–	–	–	OK253600	–
	CSF9903	----A-	Soil (*Eucalyptus* plantation)	Zhangping, Longyan, Fujian, China	25°17′10.882″ N, 117°27′33.635″ E	S.F. Chen, Q.L. Liu and F.F. Liu	–	–	–	–	OK253601	–
	CSF9904	----A-	Soil (*Eucalyptus* plantation)	Zhangping, Longyan, Fujian, China	25°17′10.882″ N, 117°27′33.635″ E	S.F. Chen, Q.L. Liu and F.F. Liu	–	–	–	–	OK253602	–
	CSF9906	----A-	Soil (*Eucalyptus* plantation)	Zhangping, Longyan, Fujian, China	25°17′10.882″ N, 117°27′33.635″ E	S.F. Chen, Q.L. Liu and F.F. Liu	–	–	–	–	OK253603	–
	CSF9927	----A-	Soil (*Phyllostachys heterocycla*)	Xinluo, Longyan, Fujian, China	25°07′31.133″ N, 116°51′37.485″ E	S.F. Chen, Q.L. Liu and F.F. Liu	–	–	–	–	OK253604	–
	CSF9928	----A-	Soil (*Phyllostachys heterocycla*)	Xinluo, Longyan, Fujian, China	25°07′31.133″ N, 116°51′37.485″ E	S.F. Chen, Q.L. Liu and F.F. Liu	–	–	–	–	OK253605	–
	CSF9929	----A-	Soil (*Phyllostachys heterocycla*)	Xinluo, Longyan, Fujian, China	25°07′31.133″ N, 116°51′37.485″ E	S.F. Chen, Q.L. Liu and F.F. Liu	–	–	–	–	OK253606	–
	CSF9931	----A-	Soil (*Phyllostachys heterocycla*)	Xinluo, Longyan, Fujian, China	25°07′31.133″ N, 116°51′37.485″ E	S.F. Chen, Q.L. Liu and F.F. Liu	–	–	–	–	OK253607	–
	CSF9940	----A-	Soil (*Eucalyptus* plantation)	Xinluo, Longyan, Fujian, China	25°07′08.597″ N, 116°44′42.257″ E	S.F. Chen, Q.L. Liu and F.F. Liu	–	–	–	–	OK253608	–
	CSF9944	----A-	Soil (*Eucalyptus* plantation)	Xinluo, Longyan, Fujian, China	25°07′08.597″ N, 116°44′42.257″ E	S.F. Chen, Q.L. Liu and F.F. Liu	–	–	–	–	OK253609	–
	CSF9954	----A-	Soil (natural forest area)	Liancheng, Longyan, Fujian, China	25°26′14.348″ N, 116°38′42.400″ E	S.F. Chen, Q.L. Liu and F.F. Liu	–	–	–	–	OK253610	–
	CSF9955	----A-	Soil (natural forest area)	Liancheng, Longyan, Fujian, China	25°26′14.348″ N, 116°38′42.400″ E	S.F. Chen, Q.L. Liu and F.F. Liu	–	–	–	–	OK253611	–
	CSF9956	----A-	Soil (natural forest area)	Liancheng, Longyan, Fujian, China	25°26′14.348″ N, 116°38′42.400″ E	S.F. Chen, Q.L. Liu and F.F. Liu	–	–	–	–	OK253612	–
	CSF9958	----A-	Soil (natural forest area)	Liancheng, Longyan, Fujian, China	25°26′14.348″ N, 116°38′42.400″ E	S.F. Chen, Q.L. Liu and F.F. Liu	–	–	–	–	OK253613	–
	CSF9965	----A-	Soil (natural forest area)	Liancheng, Longyan, Fujian, China	25°26′14.348″ N, 116°38′42.400″ E	S.F. Chen, Q.L. Liu and F.F. Liu	–	–	–	–	OK253614	–
	CSF9966	----A-	Soil (natural forest area)	Liancheng, Longyan, Fujian, China	25°26′14.348″ N, 116°38′42.400″ E	S.F. Chen, Q.L. Liu and F.F. Liu	–	–	–	–	OK253615	–
	CSF9967	----A-	Soil (natural forest area)	Liancheng, Longyan, Fujian, China	25°26′14.348″ N, 116°38′42.400″ E	S.F. Chen, Q.L. Liu and F.F. Liu	–	–	–	–	OK253616	–
	CSF9968	----A-	Soil (natural forest area)	Liancheng, Longyan, Fujian, China	25°26′14.348″ N, 116°38′42.400″ E	S.F. Chen, Q.L. Liu and F.F. Liu	–	–	–	–	OK253617	–
	CSF9971	----A-	Soil (natural forest area)	Liancheng, Longyan, Fujian, China	25°26′14.348″ N, 116°38′42.400″ E	S.F. Chen, Q.L. Liu and F.F. Liu	–	–	–	–	OK253618	–
	CSF9979	----A-	Soil (natural forest area)	Liancheng, Longyan, Fujian, China	25°26′14.348″ N, 116°38′42.400″ E	S.F. Chen, Q.L. Liu and F.F. Liu	–	–	–	–	OK253619	–
	CSF9980	----A-	Soil (natural forest area)	Liancheng, Longyan, Fujian, China	25°26′14.348″ N, 116°38′42.400″ E	S.F. Chen, Q.L. Liu and F.F. Liu	–	–	–	–	OK253620	–
	CSF9981	----A-	Soil (natural forest area)	Liancheng, Longyan, Fujian, China	25°26′14.348″ N, 116°38′42.400″ E	S.F. Chen, Q.L. Liu and F.F. Liu	–	–	–	–	OK253621	–
	CSF9983	----A-	Soil (natural forest area)	Liancheng, Longyan, Fujian, China	25°26′14.348″ N, 116°38′42.400″ E	S.F. Chen, Q.L. Liu and F.F. Liu	–	–	–	–	OK253622	–
	CSF9984	----A-	Soil (natural forest area)	Liancheng, Longyan, Fujian, China	25°26′14.348″ N, 116°38′42.400″ E	S.F. Chen, Q.L. Liu and F.F. Liu	–	–	–	–	OK253623	–
	CSF9985	----A-	Soil (natural forest area)	Liancheng, Longyan, Fujian, China	25°26′14.348″ N, 116°38′42.400″ E	S.F. Chen, Q.L. Liu and F.F. Liu	–	–	–	–	OK253624	–
	CSF9986	----A-	Soil (natural forest area)	Liancheng, Longyan, Fujian, China	25°26′14.348″ N, 116°38′42.400″ E	S.F. Chen, Q.L. Liu and F.F. Liu	–	–	–	–	OK253625	–
	CSF9988	----A-	Soil (natural forest area)	Liancheng, Longyan, Fujian, China	25°26′14.348″ N, 116°38′42.400″ E	S.F. Chen, Q.L. Liu and F.F. Liu	–	–	–	–	OK253626	–
	CSF9990	----A-	Soil (natural forest area)	Liancheng, Longyan, Fujian, China	25°26′14.348″ N, 116°38′42.400″ E	S.F. Chen, Q.L. Liu and F.F. Liu	–	–	–	–	OK253627	–
	CSF9991	----A-	Soil (natural forest area)	Liancheng, Longyan, Fujian, China	25°26′14.348″ N, 116°38′42.400″ E	S.F. Chen, Q.L. Liu and F.F. Liu	–	–	–	–	OK253628	–
	CSF9992	----A-	Soil (natural forest area)	Liancheng, Longyan, Fujian, China	25°26′14.348″ N, 116°38′42.400″ E	S.F. Chen, Q.L. Liu and F.F. Liu	–	–	–	–	OK253629	–
	CSF9993	----A-	Soil (natural forest area)	Liancheng, Longyan, Fujian, China	25°26′14.348″ N, 116°38′42.400″ E	S.F. Chen, Q.L. Liu and F.F. Liu	–	–	–	–	OK253630	–
	CSF10029	----A-	Soil (*Eucalyptus* plantation)	Yongan, Sanming, Fujian, China	25°55′10.860″ N, 117°16′39.591″ E	S.F. Chen, Q.L. Liu and F.F. Liu	–	–	–	–	OK253631	–
	CSF10030	----A-	Soil (*Eucalyptus* plantation)	Yongan, Sanming, Fujian, China	25°55′10.860″ N, 117°16′39.591″ E	S.F. Chen, Q.L. Liu and F.F. Liu	–	–	–	–	OK253632	–
	CSF10031	----A-	Soil (*Eucalyptus* plantation)	Yongan, Sanming, Fujian, China	25°55′10.860″ N, 117°16′39.591″ E	S.F. Chen, Q.L. Liu and F.F. Liu	–	–	–	–	OK253633	–
	CSF10033	----A-	Soil (*Eucalyptus* plantation)	Yongan, Sanming, Fujian, China	25°55′10.860″ N, 117°16′39.591″ E	S.F. Chen, Q.L. Liu and F.F. Liu	–	–	–	–	OK253634	–
	CSF10035	----A-	Soil (*Eucalyptus* plantation)	Yongan, Sanming, Fujian, China	25°55′10.860″ N, 117°16′39.591″ E	S.F. Chen, Q.L. Liu and F.F. Liu	–	–	–	–	OK253635	–
	CSF10036	----A-	Soil (*Eucalyptus* plantation)	Yongan, Sanming, Fujian, China	25°55′10.860″ N, 117°16′39.591″ E	S.F. Chen, Q.L. Liu and F.F. Liu	–	–	–	–	OK253636	–
	CSF10037	----A-	Soil (*Eucalyptus* plantation)	Yongan, Sanming, Fujian, China	25°55′10.860″ N, 117°16′39.591″ E	S.F. Chen, Q.L. Liu and F.F. Liu	–	–	–	–	OK253637	–
	CSF10042	----A-	Soil (*Eucalyptus* plantation)	Yongan, Sanming, Fujian, China	25°55′10.860″ N, 117°16′39.591″ E	S.F. Chen, Q.L. Liu and F.F. Liu	–	–	–	–	OK253638	–
	CSF10043	----A-	Soil (*Eucalyptus* plantation)	Yongan, Sanming, Fujian, China	25°55′10.860″ N, 117°16′39.591″ E	S.F. Chen, Q.L. Liu and F.F. Liu	–	–	–	–	OK253639	–
	CSF10048	----A-	Soil (*Cunninghamia lanceolata*)	Qingliu, Sanming, Fujian, China	26°07′23.497″ N, 116°53′00.762″ E	S.F. Chen, Q.L. Liu and F.F. Liu	–	–	–	–	OK253640	–
	CSF10049	----A-	Soil (*Cunninghamia lanceolata*)	Qingliu, Sanming, Fujian, China	26°07′23.497″ N, 116°53′00.762″ E	S.F. Chen, Q.L. Liu and F.F. Liu	–	–	–	–	OK253641	–
	CSF10051	----A-	Soil (*Cunninghamia lanceolata*)	Qingliu, Sanming, Fujian, China	26°07′23.497″ N, 116°53′00.762″ E	S.F. Chen, Q.L. Liu and F.F. Liu	–	–	–	–	OK253642	–
	CSF10052	----A-	Soil (*Cunninghamia lanceolata*)	Qingliu, Sanming, Fujian, China	26°07′23.497″ N, 116°53′00.762″ E	S.F. Chen, Q.L. Liu and F.F. Liu	–	–	–	–	OK253643	–
	CSF10063	----A-	Soil (*Eucalyptus* plantation)	Yanping, Nanping, Fujian, China	26°46′19.651″ N, 117°57′37.233″ E	S.F. Chen, Q.L. Liu and F.F. Liu	–	–	–	–	OK253644	–
	CSF10065	----A-	Soil (*Eucalyptus* plantation)	Yanping, Nanping, Fujian, China	26°46′19.651″ N, 117°57′37.233″ E	S.F. Chen, Q.L. Liu and F.F. Liu	–	–	–	–	OK253645	–
	CSF10066	----A-	Soil (*Eucalyptus* plantation)	Yanping, Nanping, Fujian, China	26°46′19.651″ N, 117°57′37.233″ E	S.F. Chen, Q.L. Liu and F.F. Liu	–	–	–	–	OK253646	–
	CSF10067	----A-	Soil (*Eucalyptus* plantation)	Yanping, Nanping, Fujian, China	26°46′19.651″ N, 117°57′37.233″ E	S.F. Chen, Q.L. Liu and F.F. Liu	–	–	–	–	OK253647	–
	CSF10069	----A-	Soil (*Eucalyptus* plantation)	Yanping, Nanping, Fujian, China	26°46′19.651″ N, 117°57′37.233″ E	S.F. Chen, Q.L. Liu and F.F. Liu	–	–	–	–	OK253648	–
	CSF10074	----A-	Soil (natural forest area)	Yanping, Nanping, Fujian, China	26°42′26.672″ N, 118°07′58.317″ E	S.F. Chen, Q.L. Liu and F.F. Liu	–	–	–	–	OK253649	–
	CSF10083	----A-	Soil (*Eucalyptus* plantation)	Minhou, Fuzhou, Fujian, China	26°15′04.285″ N, 119°02′38.917″ E	S.F. Chen, Q.L. Liu and F.F. Liu	–	–	–	–	OK253650	–
	CSF10099	----A-	Soil (*Eucalyptus* plantation)	Minhou, Fuzhou, Fujian, China	26°15′04.285″ N, 119°02′38.917″ E	S.F. Chen, Q.L. Liu and F.F. Liu	–	–	–	–	OK253651	–
	CSF10100	----A-	Soil (*Eucalyptus* plantation)	Minhou, Fuzhou, Fujian, China	26°15′04.285″ N, 119°02′38.917″ E	S.F. Chen, Q.L. Liu and F.F. Liu	–	–	–	–	OK253652	–
	CSF10101	----A-	Soil (*Eucalyptus* plantation)	Minhou, Fuzhou, Fujian, China	26°15′04.285″ N, 119°02′38.917″ E	S.F. Chen, Q.L. Liu and F.F. Liu	–	–	–	–	OK253653	–
	CSF10102	----A-	Soil (*Eucalyptus* plantation)	Minhou, Fuzhou, Fujian, China	26°15′04.285″ N, 119°02′38.917″ E	S.F. Chen, Q.L. Liu and F.F. Liu	–	–	–	–	OK253654	–
	CSF10103	----A-	Soil (*Eucalyptus* plantation)	Minhou, Fuzhou, Fujian, China	26°15′04.285″ N, 119°02′38.917″ E	S.F. Chen, Q.L. Liu and F.F. Liu	–	–	–	–	OK253655	–
	CSF10104	----A-	Soil (*Eucalyptus* plantation)	Minhou, Fuzhou, Fujian, China	26°15′04.285″ N, 119°02′38.917″ E	S.F. Chen, Q.L. Liu and F.F. Liu	–	–	–	–	OK253656	–
	CSF10106	----A-	Soil (*Eucalyptus* plantation)	Minhou, Fuzhou, Fujian, China	26°15′04.285″ N, 119°02′38.917″ E	S.F. Chen, Q.L. Liu and F.F. Liu	–	–	–	–	OK253657	–
	CSF10107	----A-	Soil (*Eucalyptus* plantation)	Minhou, Fuzhou, Fujian, China	26°15′04.285″ N, 119°02′38.917″ E	S.F. Chen, Q.L. Liu and F.F. Liu	–	–	–	–	OK253658	–
	CSF10108	----A-	Soil (*Eucalyptus* plantation)	Minhou, Fuzhou, Fujian, China	26°15′04.285″ N, 119°02′38.917″ E	S.F. Chen, Q.L. Liu and F.F. Liu	–	–	–	–	OK253659	–
	CSF10109	----A-	Soil (*Eucalyptus* plantation)	Minhou, Fuzhou, Fujian, China	26°15′04.285″ N, 119°02′38.917″ E	S.F. Chen, Q.L. Liu and F.F. Liu	–	–	–	–	OK253660	–
	CSF10111	----A-	Soil (*Eucalyptus* plantation)	Minhou, Fuzhou, Fujian, China	26°15′04.285″ N, 119°02′38.917″ E	S.F. Chen, Q.L. Liu and F.F. Liu	–	–	–	–	OK253661	–
	CSF10113	----A-	Soil (*Eucalyptus* plantation)	Minhou, Fuzhou, Fujian, China	26°15′04.285″ N, 119°02′38.917″ E	S.F. Chen, Q.L. Liu and F.F. Liu	–	–	–	–	OK253662	–
	CSF10114	----A-	Soil (*Eucalyptus* plantation)	Minhou, Fuzhou, Fujian, China	26°15′04.285″ N, 119°02′38.917″ E	S.F. Chen, Q.L. Liu and F.F. Liu	–	–	–	–	OK253663	–
	CSF10115	----A-	Soil (*Eucalyptus* plantation)	Minhou, Fuzhou, Fujian, China	26°15′04.285″ N, 119°02′38.917″ E	S.F. Chen, Q.L. Liu and F.F. Liu	–	–	–	–	OK253664	–
	CSF10116	----A-	Soil (*Eucalyptus* plantation)	Minhou, Fuzhou, Fujian, China	26°15′04.285″ N, 119°02′38.917″ E	S.F. Chen, Q.L. Liu and F.F. Liu	–	–	–	–	OK253665	–
	CSF10117	----A-	Soil (*Eucalyptus* plantation)	Minhou, Fuzhou, Fujian, China	26°15′04.285″ N, 119°02′38.917″ E	S.F. Chen, Q.L. Liu and F.F. Liu	–	–	–	–	OK253666	–
	CSF10118	----A-	Soil (*Eucalyptus* plantation)	Minhou, Fuzhou, Fujian, China	26°15′04.285″ N, 119°02′38.917″ E	S.F. Chen, Q.L. Liu and F.F. Liu	–	–	–	–	OK253667	–
	CSF10120	----A-	Soil (*Eucalyptus* plantation)	Minhou, Fuzhou, Fujian, China	26°15′04.285″ N, 119°02′38.917″ E	S.F. Chen, Q.L. Liu and F.F. Liu	–	–	–	–	OK253668	–
*Ca. hongkongensis*	CSF7124 ^e^	AAAAAA	Soil (natural forest area)	Cangshan, Fuzhou, Fujian, China	26°5′16.2″ N, 119°14′19.8″ E	S.F. Chen, Q.L. Liu and F.F. Liu	OK253081	OK253192	OK253336	OK253438	OK253669	OK253900
	CSF9784 ^e^	AAAAAA	Soil (*Eucalyptus* plantation)	Hua’an, Zhangzhou, Fujian, China	24°53′49.369″ N, 117°32′45.070″ E	S.F. Chen, Q.L. Liu and F.F. Liu	OK253082	OK253193	OK253337	OK253439	OK253670	OK253901
	CSF9794 ^e^	ABAAAA	Soil (*Eucalyptus* plantation)	Hua’an, Zhangzhou, Fujian, China	24°53′49.369″ N, 117°32′45.070″ E	S.F. Chen, Q.L. Liu and F.F. Liu	OK253083	OK253194	OK253338	OK253440	OK253671	OK253902
	CSF9799 ^e^	ABAAAA	Soil (*Eucalyptus* plantation)	Hua’an, Zhangzhou, Fujian, China	24°53′49.369″ N, 117°32′45.070″ E	S.F. Chen, Q.L. Liu and F.F. Liu	OK253084	OK253195	OK253339	OK253441	OK253672	OK253903
	CSF7139	-AA-AA	Soil (natural forest area)	Cangshan, Fuzhou, Fujian, China	26°5′16.2″ N, 119°14′19.8″ E	S.F. Chen, Q.L. Liu and F.F. Liu	–	OK253196	OK253340	–	OK253673	OK253904
	CSF9804	-AA-AA	Soil (*Eucalyptus* plantation)	Hua’an, Zhangzhou, Fujian, China	24°53′49.369″ N, 117°32′45.070″ E	S.F. Chen, Q.L. Liu and F.F. Liu	–	OK253197	OK253341	–	OK253674	OK253905
	CSF9819	-AA-AA	Soil (*Eucalyptus* plantation)	Hua’an, Zhangzhou, Fujian, China	24°53′49.369″ N, 117°32′45.070″ E	S.F. Chen, Q.L. Liu and F.F. Liu	–	OK253198	OK253342	–	OK253675	OK253906
	CSF10093	-AA-AA	Soil (*Eucalyptus* plantation)	Minhou, Fuzhou, Fujian, China	26°15′04.285″ N, 119°02′38.917″ E	S.F. Chen, Q.L. Liu and F.F. Liu	–	OK253199	OK253343	–	OK253676	OK253907
	CSF10096	-AA-AA	Soil (*Eucalyptus* plantation)	Minhou, Fuzhou, Fujian, China	26°15′04.285″ N, 119°02′38.917″ E	S.F. Chen, Q.L. Liu and F.F. Liu	–	OK253200	OK253344	–	OK253677	OK253908
	CSF9829	-BA-AA	Soil (*Eucalyptus* plantation)	Hua’an, Zhangzhou, Fujian, China	24°58′22.263″ N, 117°31′09.708″ E	S.F. Chen, Q.L. Liu and F.F. Liu	–	OK253201	OK253345	–	OK253678	OK253909
	CSF7134	----A-	Soil (natural forest area)	Cangshan, Fuzhou, Fujian, China	26°5′16.2″ N, 119°14′19.8″ E	S.F. Chen, Q.L. Liu and F.F. Liu	–	–	–	–	OK253679	–
	CSF7135	----A-	Soil (natural forest area)	Cangshan, Fuzhou, Fujian, China	26°5′16.2″ N, 119°14′19.8″ E	S.F. Chen, Q.L. Liu and F.F. Liu	–	–	–	–	OK253680	–
	CSF7136	----A-	Soil (natural forest area)	Cangshan, Fuzhou, Fujian, China	26°5′16.2″ N, 119°14′19.8″ E	S.F. Chen, Q.L. Liu and F.F. Liu	–	–	–	–	OK253681	–
	CSF7138	----A-	Soil (natural forest area)	Cangshan, Fuzhou, Fujian, China	26°5′16.2″ N, 119°14′19.8″ E	S.F. Chen, Q.L. Liu and F.F. Liu	–	–	–	–	OK253682	–
	CSF9781	----A-	Soil (*Eucalyptus* plantation)	Hua’an, Zhangzhou, Fujian, China	24°53′49.369″ N, 117°32′45.070″ E	S.F. Chen, Q.L. Liu and F.F. Liu	–	–	–	–	OK253683	–
	CSF9782	----A-	Soil (*Eucalyptus* plantation)	Hua’an, Zhangzhou, Fujian, China	24°53′49.369″ N, 117°32′45.070″ E	S.F. Chen, Q.L. Liu and F.F. Liu	–	–	–	–	OK253684	–
	CSF9783	----A-	Soil (*Eucalyptus* plantation)	Hua’an, Zhangzhou, Fujian, China	24°53′49.369″ N, 117°32′45.070″ E	S.F. Chen, Q.L. Liu and F.F. Liu	–	–	–	–	OK253685	–
	CSF9785	----A-	Soil (*Eucalyptus* plantation)	Hua’an, Zhangzhou, Fujian, China	24°53′49.369″ N, 117°32′45.070″ E	S.F. Chen, Q.L. Liu and F.F. Liu	–	–	–	–	OK253686	–
	CSF9791	----A-	Soil (*Eucalyptus* plantation)	Hua’an, Zhangzhou, Fujian, China	24°53′49.369″ N, 117°32′45.070″ E	S.F. Chen, Q.L. Liu and F.F. Liu	–	–	–	–	OK253687	–
	CSF9792	----A-	Soil (*Eucalyptus* plantation)	Hua’an, Zhangzhou, Fujian, China	24°53′49.369″ N, 117°32′45.070″ E	S.F. Chen, Q.L. Liu and F.F. Liu	–	–	–	–	OK253688	–
	CSF9793	----A-	Soil (*Eucalyptus* plantation)	Hua’an, Zhangzhou, Fujian, China	24°53′49.369″ N, 117°32′45.070″ E	S.F. Chen, Q.L. Liu and F.F. Liu	–	–	–	–	OK253689	–
	CSF9795	----A-	Soil (*Eucalyptus* plantation)	Hua’an, Zhangzhou, Fujian, China	24°53′49.369″ N, 117°32′45.070″ E	S.F. Chen, Q.L. Liu and F.F. Liu	–	–	–	–	OK253690	–
	CSF9796	----A-	Soil (*Eucalyptus* plantation)	Hua’an, Zhangzhou, Fujian, China	24°53′49.369″ N, 117°32′45.070″ E	S.F. Chen, Q.L. Liu and F.F. Liu	–	–	–	–	OK253691	–
	CSF9800	----A-	Soil (*Eucalyptus* plantation)	Hua’an, Zhangzhou, Fujian, China	24°53′49.369″ N, 117°32′45.070″ E	S.F. Chen, Q.L. Liu and F.F. Liu	–	–	–	–	OK253692	–
	CSF9801	----A-	Soil (*Eucalyptus* plantation)	Hua’an, Zhangzhou, Fujian, China	24°53′49.369″ N, 117°32′45.070″ E	S.F. Chen, Q.L. Liu and F.F. Liu	–	–	–	–	OK253693	–
	CSF9802	----A-	Soil (*Eucalyptus* plantation)	Hua’an, Zhangzhou, Fujian, China	24°53′49.369″ N, 117°32′45.070″ E	S.F. Chen, Q.L. Liu and F.F. Liu	–	–	–	–	OK253694	–
	CSF9803	----A-	Soil (*Eucalyptus* plantation)	Hua’an, Zhangzhou, Fujian, China	24°53′49.369″ N, 117°32′45.070″ E	S.F. Chen, Q.L. Liu and F.F. Liu	–	–	–	–	OK253695	–
	CSF9816	----A-	Soil (*Eucalyptus* plantation)	Hua’an, Zhangzhou, Fujian, China	24°53′49.369″ N, 117°32′45.070″ E	S.F. Chen, Q.L. Liu and F.F. Liu	–	–	–	–	OK253696	–
	CSF9817	----A-	Soil (*Eucalyptus* plantation)	Hua’an, Zhangzhou, Fujian, China	24°53′49.369″ N, 117°32′45.070″ E	S.F. Chen, Q.L. Liu and F.F. Liu	–	–	–	–	OK253697	–
	CSF9818	----A-	Soil (*Eucalyptus* plantation)	Hua’an, Zhangzhou, Fujian, China	24°53′49.369″ N, 117°32′45.070″ E	S.F. Chen, Q.L. Liu and F.F. Liu	–	–	–	–	OK253698	–
	CSF9820	----A-	Soil (*Eucalyptus* plantation)	Hua’an, Zhangzhou, Fujian, China	24°53′49.369″ N, 117°32′45.070″ E	S.F. Chen, Q.L. Liu and F.F. Liu	–	–	–	–	OK253699	–
	CSF9826	----A-	Soil (*Eucalyptus* plantation)	Hua’an, Zhangzhou, Fujian, China	24°58′22.263″ N, 117°31′09.708″ E	S.F. Chen, Q.L. Liu and F.F. Liu	–	–	–	–	OK253700	–
	CSF9827	----A-	Soil (*Eucalyptus* plantation)	Hua’an, Zhangzhou, Fujian, China	24°58′22.263″ N, 117°31′09.708″ E	S.F. Chen, Q.L. Liu and F.F. Liu	–	–	–	–	OK253701	–
	CSF9828	----A-	Soil (*Eucalyptus* plantation)	Hua’an, Zhangzhou, Fujian, China	24°58′22.263″ N, 117°31′09.708″ E	S.F. Chen, Q.L. Liu and F.F. Liu	–	–	–	–	OK253702	–
	CSF9830	----A-	Soil (*Eucalyptus* plantation)	Hua’an, Zhangzhou, Fujian, China	24°58′22.263″ N, 117°31′09.708″ E	S.F. Chen, Q.L. Liu and F.F. Liu	–	–	–	–	OK253703	–
	CSF10094	----A-	Soil (*Eucalyptus* plantation)	Minhou, Fuzhou, Fujian, China	26°15′04.285″ N, 119°02′38.917″ E	S.F. Chen, Q.L. Liu and F.F. Liu	–	–	–	–	OK253704	–
	CSF10095	----A-	Soil (*Eucalyptus* plantation)	Minhou, Fuzhou, Fujian, China	26°15′04.285″ N, 119°02′38.917″ E	S.F. Chen, Q.L. Liu and F.F. Liu	–	–	–	–	OK253705	–
*Ca. ilicicola*	CSF9862 ^e^	AAAAAA	Soil (*Eucalyptus* plantation)	Zhangping, Longyan, Fujian, China	25°17′10.882″ N, 117°27′33.635″ E	S.F. Chen, Q.L. Liu and F.F. Liu	OK253085	OK253202	OK253346	OK253442	OK253706	OK253910
	CSF9863 ^e^	AAAAAA	Soil (*Eucalyptus* plantation)	Zhangping, Longyan, Fujian, China	25°17′10.882″ N, 117°27′33.635″ E	S.F. Chen, Q.L. Liu and F.F. Liu	OK253086	OK253203	OK253347	OK253443	OK253707	OK253911
	CSF9864	-AA-AA	Soil (*Eucalyptus* plantation)	Zhangping, Longyan, Fujian, China	25°17′10.882″ N, 117°27′33.635″ E	S.F. Chen, Q.L. Liu and F.F. Liu	–	OK253204	OK253348	–	OK253708	OK253912
	CSF9865	-AA-AA	Soil (*Eucalyptus* plantation)	Zhangping, Longyan, Fujian, China	25°17′10.882″ N, 117°27′33.635″ E	S.F. Chen, Q.L. Liu and F.F. Liu	–	OK253205	OK253349	–	OK253709	OK253913
	CSF9866	-AA-AA	Soil (*Eucalyptus* plantation)	Zhangping, Longyan, Fujian, China	25°17′10.882″ N, 117°27′33.635″ E	S.F. Chen, Q.L. Liu and F.F. Liu	–	OK253206	OK253350	–	OK253710	OK253914
*Ca. kyotensis*	CSF7130 ^e^	AAAAAA	Soil (natural forest area)	Cangshan, Fuzhou, Fujian, China	26°5′16.2″ N, 119°14′19.8″ E	S.F. Chen, Q.L. Liu and F.F. Liu	OK253087	OK253207	OK253351	OK253444	OK253711	OK253915
	CSF10088 ^e^	AAAAAA	Soil (*Eucalyptus* plantation)	Minhou, Fuzhou, Fujian, China	26°15′04.285″ N, 119°02′38.917″ E	S.F. Chen, Q.L. Liu and F.F. Liu	OK253088	OK253208	OK253352	OK253445	OK253712	OK253916
	CSF9834 ^e^	AAA-AB	Soil (*Eucalyptus* plantation)	Hua’an, Zhangzhou, Fujian, China	24°58′22.263″ N, 117°31′09.708″ E	S.F. Chen, Q.L. Liu and F.F. Liu	OK253089	OK253209	OK253353	N/A	OK253713	OK253917
	CSF9910 ^e^	AAAAAB	Soil (*Phyllostachys heterocycla*)	Xinluo, Longyan, Fujian, China	25°07′31.133″ N, 116°51′37.485″ E	S.F. Chen, Q.L. Liu and F.F. Liu	OK253090	OK253210	OK253354	OK253446	OK253714	OK253918
	CSF10014 ^e^	AAAAAC	Soil (*Eucalyptus* plantation)	Yongan, Sanming, Fujian, China	25°55′10.860″ N, 117°16′39.591″ E	S.F. Chen, Q.L. Liu and F.F. Liu	OK253091	OK253211	OK253355	OK253447	OK253715	OK253919
	CSF10080 ^e^	AAAAAD	Soil (*Eucalyptus* plantation)	Minhou, Fuzhou, Fujian, China	26°15′04.285″ N, 119°02′38.917″ E	S.F. Chen, Q.L. Liu and F.F. Liu	OK253092	OK253212	OK253356	OK253448	OK253716	OK253920
	CSF10086 ^e^	AAAAAE	Soil (*Eucalyptus* plantation)	Minhou, Fuzhou, Fujian, China	26°15′04.285″ N, 119°02′38.917″ E	S.F. Chen, Q.L. Liu and F.F. Liu	OK253093	OK253213	OK253357	OK253449	OK253717	OK253921
	CSF10053 ^e^	AAAABB	Soil (*Pinus massoniana*)	Qingliu, Sanming, Fujian, China	26°10′54.311″ N, 116°52′50.901″ E	S.F. Chen, Q.L. Liu and F.F. Liu	OK253094	OK253214	OK253358	OK253450	OK253718	OK253922
	CSF10054 ^e^	AAAABB	Soil (*Pinus massoniana*)	Qingliu, Sanming, Fujian, China	26°10′54.311″ N, 116°52′50.901″ E	S.F. Chen, Q.L. Liu and F.F. Liu	OK253095	OK253215	OK253359	OK253451	OK253719	OK253923
	CSF9922 ^e^	AAAABF	Soil (*Phyllostachys heterocycla*)	Xinluo, Longyan, Fujian, China	25°07′31.133″ N, 116°51′37.485″ E	S.F. Chen, Q.L. Liu and F.F. Liu	OK253096	OK253216	OK253360	OK253452	OK253720	OK253924
	CSF9923 ^e^	AAAABF	Soil (*Phyllostachys heterocycla*)	Xinluo, Longyan, Fujian, China	25°07′31.133″ N, 116°51′37.485″ E	S.F. Chen, Q.L. Liu and F.F. Liu	OK253097	OK253217	OK253361	OK253453	OK253721	OK253925
	CSF9949 ^e^	AAAADB	Soil (*Eucalyptus* plantation)	Xinluo, Longyan, Fujian, China	25°07′08.597″ N, 116°44′42.257″ E	S.F. Chen, Q.L. Liu and F.F. Liu	OK253098	OK253218	OK253362	OK253454	OK253722	OK253926
	CSF9951 ^e^	AAAADB	Soil (*Eucalyptus* plantation)	Xinluo, Longyan, Fujian, China	25°07′08.597″ N, 116°44′42.257″ E	S.F. Chen, Q.L. Liu and F.F. Liu	OK253099	OK253219	OK253363	OK253455	OK253723	OK253927
	CSF9932 ^e^	AAAADG	Soil (*Eucalyptus* plantation)	Xinluo, Longyan, Fujian, China	25°07′08.597″ N, 116°44′42.257″ E	S.F. Chen, Q.L. Liu and F.F. Liu	OK253100	OK253220	OK253364	OK253456	OK253724	OK253928
	CSF9935 ^e^	AAAADG	Soil (*Eucalyptus* plantation)	Xinluo, Longyan, Fujian, China	25°07′08.597″ N, 116°44′42.257″ E	S.F. Chen, Q.L. Liu and F.F. Liu	OK253101	OK253221	OK253365	OK253457	OK253725	OK253929
	CSF9936 ^e^	AAAADG	Soil (*Eucalyptus* plantation)	Xinluo, Longyan, Fujian, China	25°07′08.597″ N, 116°44′42.257″ E	S.F. Chen, Q.L. Liu and F.F. Liu	OK253102	OK253222	OK253366	OK253458	OK253726	OK253930
	CSF10020 ^e^	AAAAEA	Soil (*Eucalyptus* plantation)	Yongan, Sanming, Fujian, China	25°55′10.860″ N, 117°16′39.591″ E	S.F. Chen, Q.L. Liu and F.F. Liu	OK253103	OK253223	OK253367	OK253459	OK253727	OK253931
	CSF10021 ^e^	AAAAEA	Soil (*Eucalyptus* plantation)	Yongan, Sanming, Fujian, China	25°55′10.860″ N, 117°16′39.591″ E	S.F. Chen, Q.L. Liu and F.F. Liu	OK253104	OK253224	OK253368	OK253460	OK253728	OK253932
	CSF10009 ^e^	AAABBH	Soil (*Eucalyptus* plantation)	Liancheng, Longyan, Fujian, China	25°33′06.994″ N, 116°41′42.328″ E	S.F. Chen, Q.L. Liu and F.F. Liu	OK253105	OK253225	OK253369	OK253461	OK253729	OK253933
	CSF10010 ^e^	AAABBH	Soil (*Eucalyptus* plantation)	Liancheng, Longyan, Fujian, China	25°33′06.994″ N, 116°41′42.328″ E	S.F. Chen, Q.L. Liu and F.F. Liu	OK253106	OK253226	OK253370	OK253462	OK253730	OK253934
	CSF9997 ^e^	AABAAB	Soil (*Eucalyptus* plantation)	Liancheng, Longyan, Fujian, China	25°33′06.994″ N, 116°41′42.328″ E	S.F. Chen, Q.L. Liu and F.F. Liu	OK253107	OK253227	OK253371	OK253463	OK253731	OK253935
	CSF9969 ^e^	AABACB	Soil (natural forest area)	Liancheng, Longyan, Fujian, China	25°26′14.348″ N, 116°38′42.400″ E	S.F. Chen, Q.L. Liu and F.F. Liu	OK253108	OK253228	OK253372	OK253464	OK253732	OK253936
	CSF9972 ^e^	AABACB	Soil (natural forest area)	Liancheng, Longyan, Fujian, China	25°26′14.348″ N, 116°38′42.400″ E	S.F. Chen, Q.L. Liu and F.F. Liu	OK253109	OK253229	OK253373	OK253465	OK253733	OK253937
	CSF9973 ^e^	AABACB	Soil (natural forest area)	Liancheng, Longyan, Fujian, China	25°26′14.348″ N, 116°38′42.400″ E	S.F. Chen, Q.L. Liu and F.F. Liu	OK253110	OK253230	OK253374	OK253466	OK253734	OK253938
	CSF10126 ^e^	AACAAA	Soil (*Eucalyptus* plantation)	Minhou, Fuzhou, Fujian, China	26°15′04.285″ N, 119°02′38.917″ E	S.F. Chen, Q.L. Liu and F.F. Liu	OK253111	OK253231	OK253375	OK253467	OK253735	OK253939
	CSF9962 ^e^	AACAAD	Soil (natural forest area)	Liancheng, Longyan, Fujian, China	25°26′14.348″ N, 116°38′42.400″ E	S.F. Chen, Q.L. Liu and F.F. Liu	OK253112	OK253232	OK253376	OK253468	OK253736	OK253940
	CSF10019 ^e^	AADABB	Soil (*Eucalyptus* plantation)	Yongan, Sanming, Fujian, China	25°55′10.860″ N, 117°16′39.591″ E	S.F. Chen, Q.L. Liu and F.F. Liu	OK253113	OK253233	OK253377	OK253469	OK253737	OK253941
	CSF10022 ^e^	AADABB	Soil (*Eucalyptus* plantation)	Yongan, Sanming, Fujian, China	25°55′10.860″ N, 117°16′39.591″ E	S.F. Chen, Q.L. Liu and F.F. Liu	OK253114	OK253234	OK253378	OK253470	OK253738	OK253942
	CSF10023 ^e^	AADABB	Soil (*Eucalyptus* plantation)	Yongan, Sanming, Fujian, China	25°55′10.860″ N, 117°16′39.591″ E	S.F. Chen, Q.L. Liu and F.F. Liu	OK253115	OK253235	OK253379	OK253471	OK253739	OK253943
	CSF10045 ^e^	ABAAAB	Soil (*Cunninghamia lanceolata*)	Qingliu, Sanming, Fujian, China	26°07′23.497″ N, 116°53′00.762″ E	S.F. Chen, Q.L. Liu and F.F. Liu	OK253116	OK253236	OK253380	OK253472	OK253740	OK253944
	CSF10047 ^e^	ABAAAB	Soil (*Cunninghamia lanceolata*)	Qingliu, Sanming, Fujian, China	26°07′23.497″ N, 116°53′00.762″ E	S.F. Chen, Q.L. Liu and F.F. Liu	OK253117	OK253237	OK253381	OK253473	OK253741	OK253945
	CSF9824 ^e^	ACBAAC	Soil (*Eucalyptus* plantation)	Hua’an, Zhangzhou, Fujian, China	24°53′49.369″ N, 117°32′45.070″ E	S.F. Chen, Q.L. Liu and F.F. Liu	OK253118	OK253238	OK253382	OK253474	OK253742	OK253946
	CSF10004 ^e^	ADAACB	Soil (*Eucalyptus* plantation)	Liancheng, Longyan, Fujian, China	25°33′06.994″ N, 116°41′42.328″ E	S.F. Chen, Q.L. Liu and F.F. Liu	OK253119	OK253239	OK253383	OK253475	OK253743	OK253947
	CSF10005 ^e^	ADAACB	Soil (*Eucalyptus* plantation)	Liancheng, Longyan, Fujian, China	25°33′06.994″ N, 116°41′42.328″ E	S.F. Chen, Q.L. Liu and F.F. Liu	OK253120	OK253240	OK253384	OK253476	OK253744	OK253948
	CSF7123	-AA-AB	Soil (natural forest area)	Cangshan, Fuzhou, Fujian, China	26°5′16.2″ N, 119°14′19.8″ E	S.F. Chen, Q.L. Liu and F.F. Liu	–	OK253241	OK253385	–	OK253745	OK253949
	CSF9915	-AA-AB	Soil (*Phyllostachys heterocycla*)	Xinluo, Longyan, Fujian, China	25°07′31.133″ N, 116°51′37.485″ E	S.F. Chen, Q.L. Liu and F.F. Liu	–	OK253242	OK253386	–	OK253746	OK253950
	CSF9999	-AA-AB	Soil (*Eucalyptus* plantation)	Liancheng, Longyan, Fujian, China	25°33′06.994″ N, 116°41′42.328″ E	S.F. Chen, Q.L. Liu and F.F. Liu	–	OK253243	OK253387	–	OK253747	OK253951
	CSF10124	-AA-AB	Soil (*Eucalyptus* plantation)	Minhou, Fuzhou, Fujian, China	26°15′04.285″ N, 119°02′38.917″ E	S.F. Chen, Q.L. Liu and F.F. Liu	–	OK253244	OK253388	–	OK253748	OK253952
	CSF10055	-AA-BB	Soil (*Pinus massoniana*)	Qingliu, Sanming, Fujian, China	26°10′54.311″ N, 116°52′50.901″ E	S.F. Chen, Q.L. Liu and F.F. Liu	–	OK253245	OK253389	–	OK253749	OK253953
	CSF10056	-AA-BB	Soil (*Pinus massoniana*)	Qingliu, Sanming, Fujian, China	26°10′54.311″ N, 116°52′50.901″ E	S.F. Chen, Q.L. Liu and F.F. Liu	–	OK253246	OK253390	–	OK253750	OK253954
	CSF10057	-AA-BB	Soil (*Pinus massoniana*)	Qingliu, Sanming, Fujian, China	26°10′54.311″ N, 116°52′50.901″ E	S.F. Chen, Q.L. Liu and F.F. Liu	–	OK253247	OK253391	–	OK253751	OK253955
	CSF9952	-AA-DB	Soil (*Eucalyptus* plantation)	Xinluo, Longyan, Fujian, China	25°07′08.597″ N, 116°44′42.257″ E	S.F. Chen, Q.L. Liu and F.F. Liu	–	OK253248	OK253392	–	OK253752	OK253956
	CSF9953	-AA-DB	Soil (*Eucalyptus* plantation)	Xinluo, Longyan, Fujian, China	25°07′08.597″ N, 116°44′42.257″ E	S.F. Chen, Q.L. Liu and F.F. Liu	–	OK253249	OK253393	–	OK253753	OK253957
	CSF9924	-AA-BF	Soil (*Phyllostachys heterocycla*)	Xinluo, Longyan, Fujian, China	25°07′31.133″ N, 116°51′37.485″ E	S.F. Chen, Q.L. Liu and F.F. Liu	–	OK253250	OK253394	–	OK253754	OK253958
	CSF9925	-AA-BF	Soil (*Phyllostachys heterocycla*)	Xinluo, Longyan, Fujian, China	25°07′31.133″ N, 116°51′37.485″ E	S.F. Chen, Q.L. Liu and F.F. Liu	–	OK253251	OK253395	–	OK253755	OK253959
	CSF9926	-AA-BF	Soil (*Phyllostachys heterocycla*)	Xinluo, Longyan, Fujian, China	25°07′31.133″ N, 116°51′37.485″ E	S.F. Chen, Q.L. Liu and F.F. Liu	–	OK253252	OK253396	–	OK253756	OK253960
	CSF10011	-AA-BH	Soil (*Eucalyptus* plantation)	Liancheng, Longyan, Fujian, China	25°33′06.994″ N, 116°41′42.328″ E	S.F. Chen, Q.L. Liu and F.F. Liu	–	OK253253	OK253397	–	OK253757	OK253961
	CSF10012	-AA-BH	Soil (*Eucalyptus* plantation)	Liancheng, Longyan, Fujian, China	25°33′06.994″ N, 116°41′42.328″ E	S.F. Chen, Q.L. Liu and F.F. Liu	–	OK253254	OK253398	–	OK253758	OK253962
	CSF10013	-AA-BH	Soil (*Eucalyptus* plantation)	Liancheng, Longyan, Fujian, China	25°33′06.994″ N, 116°41′42.328″ E	S.F. Chen, Q.L. Liu and F.F. Liu	–	OK253255	OK253399	–	OK253759	OK253963
	CSF10006	-DA-CB	Soil (*Eucalyptus* plantation)	Liancheng, Longyan, Fujian, China	25°33′06.994″ N, 116°41′42.328″ E	S.F. Chen, Q.L. Liu and F.F. Liu	–	OK253256	OK253400	–	OK253760	OK253964
	CSF10007	-DA-CB	Soil (*Eucalyptus* plantation)	Liancheng, Longyan, Fujian, China	25°33′06.994″ N, 116°41′42.328″ E	S.F. Chen, Q.L. Liu and F.F. Liu	–	OK253257	OK253401	–	OK253761	OK253965
	CSF10008	-DA-CB	Soil (*Eucalyptus* plantation)	Liancheng, Longyan, Fujian, China	25°33′06.994″ N, 116°41′42.328″ E	S.F. Chen, Q.L. Liu and F.F. Liu	–	OK253258	OK253402	–	OK253762	OK253966
	CSF9821	----A-	Soil (*Eucalyptus* plantation)	Hua’an, Zhangzhou, Fujian, China	24°53′49.369″ N, 117°32′45.070″ E	S.F. Chen, Q.L. Liu and F.F. Liu	–	–	–	–	OK253763	–
	CSF9822	----A-	Soil (*Eucalyptus* plantation)	Hua’an, Zhangzhou, Fujian, China	24°53′49.369″ N, 117°32′45.070″ E	S.F. Chen, Q.L. Liu and F.F. Liu	–	–	–	–	OK253764	–
	CSF9823	----A-	Soil (*Eucalyptus* plantation)	Hua’an, Zhangzhou, Fujian, China	24°53′49.369″ N, 117°32′45.070″ E	S.F. Chen, Q.L. Liu and F.F. Liu	–	–	–	–	OK253765	–
	CSF9825	----A-	Soil (*Eucalyptus* plantation)	Hua’an, Zhangzhou, Fujian, China	24°53′49.369″ N, 117°32′45.070″ E	S.F. Chen, Q.L. Liu and F.F. Liu	–	–	–	–	OK253766	–
	CSF9832	----A-	Soil (*Eucalyptus* plantation)	Hua’an, Zhangzhou, Fujian, China	24°58′22.263″ N, 117°31′09.708″ E	S.F. Chen, Q.L. Liu and F.F. Liu	–	–	–	–	OK253767	–
	CSF9833	----A-	Soil (*Eucalyptus* plantation)	Hua’an, Zhangzhou, Fujian, China	24°58′22.263″ N, 117°31′09.708″ E	S.F. Chen, Q.L. Liu and F.F. Liu	–	–	–	–	OK253768	–
	CSF9835	----A-	Soil (*Eucalyptus* plantation)	Hua’an, Zhangzhou, Fujian, China	24°58′22.263″ N, 117°31′09.708″ E	S.F. Chen, Q.L. Liu and F.F. Liu	–	–	–	–	OK253769	–
	CSF9907	----A-	Soil (*Phyllostachys heterocycla*)	Xinluo, Longyan, Fujian, China	25°07′31.133″ N, 116°51′37.485″ E	S.F. Chen, Q.L. Liu and F.F. Liu	–	–	–	–	OK253770	–
	CSF9908	----A-	Soil (*Phyllostachys heterocycla*)	Xinluo, Longyan, Fujian, China	25°07′31.133″ N, 116°51′37.485″ E	S.F. Chen, Q.L. Liu and F.F. Liu	–	–	–	–	OK253771	–
	CSF9909	----A-	Soil (*Phyllostachys heterocycla*)	Xinluo, Longyan, Fujian, China	25°07′31.133″ N, 116°51′37.485″ E	S.F. Chen, Q.L. Liu and F.F. Liu	–	–	–	–	OK253772	–
	CSF9911	----A-	Soil (*Phyllostachys heterocycla*)	Xinluo, Longyan, Fujian, China	25°07′31.133″ N, 116°51′37.485″ E	S.F. Chen, Q.L. Liu and F.F. Liu	–	–	–	–	OK253773	–
	CSF9912	----A-	Soil (*Phyllostachys heterocycla*)	Xinluo, Longyan, Fujian, China	25°07′31.133″ N, 116°51′37.485″ E	S.F. Chen, Q.L. Liu and F.F. Liu	–	–	–	–	OK253774	–
	CSF9913	----A-	Soil (*Phyllostachys heterocycla*)	Xinluo, Longyan, Fujian, China	25°07′31.133″ N, 116°51′37.485″ E	S.F. Chen, Q.L. Liu and F.F. Liu	–	–	–	–	OK253775	–
	CSF9914	----A-	Soil (*Phyllostachys heterocycla*)	Xinluo, Longyan, Fujian, China	25°07′31.133″ N, 116°51′37.485″ E	S.F. Chen, Q.L. Liu and F.F. Liu	–	–	–	–	OK253776	–
	CSF9916	----A-	Soil (*Phyllostachys heterocycla*)	Xinluo, Longyan, Fujian, China	25°07′31.133″ N, 116°51′37.485″ E	S.F. Chen, Q.L. Liu and F.F. Liu	–	–	–	–	OK253777	–
	CSF9917	----A-	Soil (*Phyllostachys heterocycla*)	Xinluo, Longyan, Fujian, China	25°07′31.133″ N, 116°51′37.485″ E	S.F. Chen, Q.L. Liu and F.F. Liu	–	–	–	–	OK253778	–
	CSF9918	----A-	Soil (*Phyllostachys heterocycla*)	Xinluo, Longyan, Fujian, China	25°07′31.133″ N, 116°51′37.485″ E	S.F. Chen, Q.L. Liu and F.F. Liu	–	–	–	–	OK253779	–
	CSF9919	----A-	Soil (*Phyllostachys heterocycla*)	Xinluo, Longyan, Fujian, China	25°07′31.133″ N, 116°51′37.485″ E	S.F. Chen, Q.L. Liu and F.F. Liu	–	–	–	–	OK253780	–
	CSF9920	----A-	Soil (*Phyllostachys heterocycla*)	Xinluo, Longyan, Fujian, China	25°07′31.133″ N, 116°51′37.485″ E	S.F. Chen, Q.L. Liu and F.F. Liu	–	–	–	–	OK253781	–
	CSF9921	----A-	Soil (*Phyllostachys heterocycla*)	Xinluo, Longyan, Fujian, China	25°07′31.133″ N, 116°51′37.485″ E	S.F. Chen, Q.L. Liu and F.F. Liu	–	–	–	–	OK253782	–
	CSF9959	----A-	Soil (natural forest area)	Liancheng, Longyan, Fujian, China	25°26′14.348″ N, 116°38′42.400″ E	S.F. Chen, Q.L. Liu and F.F. Liu	–	–	–	–	OK253783	–
	CSF9960	----A-	Soil (natural forest area)	Liancheng, Longyan, Fujian, China	25°26′14.348″ N, 116°38′42.400″ E	S.F. Chen, Q.L. Liu and F.F. Liu	–	–	–	–	OK253784	–
	CSF9961	----A-	Soil (natural forest area)	Liancheng, Longyan, Fujian, China	25°26′14.348″ N, 116°38′42.400″ E	S.F. Chen, Q.L. Liu and F.F. Liu	–	–	–	–	OK253785	–
	CSF9963	----A-	Soil (natural forest area)	Liancheng, Longyan, Fujian, China	25°26′14.348″ N, 116°38′42.400″ E	S.F. Chen, Q.L. Liu and F.F. Liu	–	–	–	–	OK253786	–
	CSF9994	----A-	Soil (*Eucalyptus* plantation)	Liancheng, Longyan, Fujian, China	25°33′06.994″ N, 116°41′42.328″ E	S.F. Chen, Q.L. Liu and F.F. Liu	–	–	–	–	OK253787	–
	CSF9995	----A-	Soil (*Eucalyptus* plantation)	Liancheng, Longyan, Fujian, China	25°33′06.994″ N, 116°41′42.328″ E	S.F. Chen, Q.L. Liu and F.F. Liu	–	–	–	–	OK253788	–
	CSF9996	----A-	Soil (*Eucalyptus* plantation)	Liancheng, Longyan, Fujian, China	25°33′06.994″ N, 116°41′42.328″ E	S.F. Chen, Q.L. Liu and F.F. Liu	–	–	–	–	OK253789	–
	CSF9998	----A-	Soil (*Eucalyptus* plantation)	Liancheng, Longyan, Fujian, China	25°33′06.994″ N, 116°41′42.328″ E	S.F. Chen, Q.L. Liu and F.F. Liu	–	–	–	–	OK253790	–
	CSF10000	----A-	Soil (*Eucalyptus* plantation)	Liancheng, Longyan, Fujian, China	25°33′06.994″ N, 116°41′42.328″ E	S.F. Chen, Q.L. Liu and F.F. Liu	–	–	–	–	OK253791	–
	CSF10001	----A-	Soil (*Eucalyptus* plantation)	Liancheng, Longyan, Fujian, China	25°33′06.994″ N, 116°41′42.328″ E	S.F. Chen, Q.L. Liu and F.F. Liu	–	–	–	–	OK253792	–
	CSF10002	----A-	Soil (*Eucalyptus* plantation)	Liancheng, Longyan, Fujian, China	25°33′06.994″ N, 116°41′42.328″ E	S.F. Chen, Q.L. Liu and F.F. Liu	–	–	–	–	OK253793	–
	CSF10003	----A-	Soil (*Eucalyptus* plantation)	Liancheng, Longyan, Fujian, China	25°33′06.994″ N, 116°41′42.328″ E	S.F. Chen, Q.L. Liu and F.F. Liu	–	–	–	–	OK253794	–
	CSF10015	----A-	Soil (*Eucalyptus* plantation)	Yongan, Sanming, Fujian, China	25°55′10.860″ N, 117°16′39.591″ E	S.F. Chen, Q.L. Liu and F.F. Liu	–	–	–	–	OK253795	–
	CSF10016	----A-	Soil (*Eucalyptus* plantation)	Yongan, Sanming, Fujian, China	25°55′10.860″ N, 117°16′39.591″ E	S.F. Chen, Q.L. Liu and F.F. Liu	–	–	–	–	OK253796	–
	CSF10044	----A-	Soil (*Cunninghamia lanceolata*)	Qingliu, Sanming, Fujian, China	26°07′23.497″ N, 116°53′00.762″ E	S.F. Chen, Q.L. Liu and F.F. Liu	–	–	–	–	OK253797	–
	CSF10046	----A-	Soil (*Cunninghamia lanceolata*)	Qingliu, Sanming, Fujian, China	26°07′23.497″ N, 116°53′00.762″ E	S.F. Chen, Q.L. Liu and F.F. Liu	–	–	–	–	OK253798	–
	CSF10084	----A-	Soil (*Eucalyptus* plantation)	Minhou, Fuzhou, Fujian, China	26°15′04.285″ N, 119°02′38.917″ E	S.F. Chen, Q.L. Liu and F.F. Liu	–	–	–	–	OK253799	–
	CSF10085	----A-	Soil (*Eucalyptus* plantation)	Minhou, Fuzhou, Fujian, China	26°15′04.285″ N, 119°02′38.917″ E	S.F. Chen, Q.L. Liu and F.F. Liu	–	–	–	–	OK253800	–
	CSF10087	----A-	Soil (*Eucalyptus* plantation)	Minhou, Fuzhou, Fujian, China	26°15′04.285″ N, 119°02′38.917″ E	S.F. Chen, Q.L. Liu and F.F. Liu	–	–	–	–	OK253801	–
	CSF10089	----A-	Soil (*Eucalyptus* plantation)	Minhou, Fuzhou, Fujian, China	26°15′04.285″ N, 119°02′38.917″ E	S.F. Chen, Q.L. Liu and F.F. Liu	–	–	–	–	OK253802	–
	CSF10090	----A-	Soil (*Eucalyptus* plantation)	Minhou, Fuzhou, Fujian, China	26°15′04.285″ N, 119°02′38.917″ E	S.F. Chen, Q.L. Liu and F.F. Liu	–	–	–	–	OK253803	–
	CSF10091	----A-	Soil (*Eucalyptus* plantation)	Minhou, Fuzhou, Fujian, China	26°15′04.285″ N, 119°02′38.917″ E	S.F. Chen, Q.L. Liu and F.F. Liu	–	–	–	–	OK253804	–
	CSF10092	----A-	Soil (*Eucalyptus* plantation)	Minhou, Fuzhou, Fujian, China	26°15′04.285″ N, 119°02′38.917″ E	S.F. Chen, Q.L. Liu and F.F. Liu	–	–	–	–	OK253805	–
	CSF10121	----A-	Soil (*Eucalyptus* plantation)	Minhou, Fuzhou, Fujian, China	26°15′04.285″ N, 119°02′38.917″ E	S.F. Chen, Q.L. Liu and F.F. Liu	–	–	–	–	OK253806	–
	CSF10122	----A-	Soil (*Eucalyptus* plantation)	Minhou, Fuzhou, Fujian, China	26°15′04.285″ N, 119°02′38.917″ E	S.F. Chen, Q.L. Liu and F.F. Liu	–	–	–	–	OK253807	–
	CSF10123	----A-	Soil (*Eucalyptus* plantation)	Minhou, Fuzhou, Fujian, China	26°15′04.285″ N, 119°02′38.917″ E	S.F. Chen, Q.L. Liu and F.F. Liu	–	–	–	–	OK253808	–
	CSF10127	----A-	Soil (*Eucalyptus* plantation)	Minhou, Fuzhou, Fujian, China	26°15′04.285″ N, 119°02′38.917″ E	S.F. Chen, Q.L. Liu and F.F. Liu	–	–	–	–	OK253809	–
	CSF10128	----A-	Soil (*Eucalyptus* plantation)	Minhou, Fuzhou, Fujian, China	26°15′04.285″ N, 119°02′38.917″ E	S.F. Chen, Q.L. Liu and F.F. Liu	–	–	–	–	OK253810	–
	CSF7122	----A-	Soil (natural forest area)	Cangshan, Fuzhou, Fujian, China	26°5′16.2″ N, 119°14′19.8″ E	S.F. Chen, Q.L. Liu and F.F. Liu	–	–	–	–	OK253811	–
	CSF7128	----A-	Soil (natural forest area)	Cangshan, Fuzhou, Fujian, China	26°5′16.2″ N, 119°14′19.8″ E	S.F. Chen, Q.L. Liu and F.F. Liu	–	–	–	–	OK253812	–
	CSF7129	----A-	Soil (natural forest area)	Cangshan, Fuzhou, Fujian, China	26°5′16.2″ N, 119°14′19.8″ E	S.F. Chen, Q.L. Liu and F.F. Liu	–	–	–	–	OK253813	–
*Ca. minensis * sp. nov.	CSF9941 ^e,h–j^; CGMCC3.18877	AAAAAA	Soil (*Eucalyptus* plantation)	Xinluo, Longyan, Fujian, China	25°07′08.597″ N, 116°44′42.257″ E	S.F. Chen, Q.L. Liu and F.F. Liu	OK253121	OK253259	OK253403	OK253477	OK253814	OK253967
	CSF9974 ^e^	AAAAAA	Soil (natural forest area)	Liancheng, Longyan, Fujian, China	25°26′14.348″ N, 116°38′42.400″ E	S.F. Chen, Q.L. Liu and F.F. Liu	OK253122	OK253260	OK253404	OK253478	OK253815	OK253968
	CSF9975 ^e,h,i^; CGMCC3.18881	AAAAAA	Soil (natural forest area)	Liancheng, Longyan, Fujian, China	25°26′14.348″ N, 116°38′42.400″ E	S.F. Chen, Q.L. Liu and F.F. Liu	OK253123	OK253261	OK253405	OK253479	OK253816	OK253969
	CSF9976 ^e^	AAAAAA	Soil (natural forest area)	Liancheng, Longyan, Fujian, China	25°26′14.348″ N, 116°38′42.400″ E	S.F. Chen, Q.L. Liu and F.F. Liu	OK253124	OK253262	OK253406	OK253480	OK253817	OK253970
	CSF9977 ^e^	AAAAAA	Soil (natural forest area)	Liancheng, Longyan, Fujian, China	25°26′14.348″ N, 116°38′42.400″ E	S.F. Chen, Q.L. Liu and F.F. Liu	OK253125	OK253263	OK253407	OK253481	OK253818	OK253971
	CSF9978 ^e^	AAAAAA	Soil (natural forest area)	Liancheng, Longyan, Fujian, China	25°26′14.348″ N, 116°38′42.400″ E	S.F. Chen, Q.L. Liu and F.F. Liu	OK253126	OK253264	OK253408	OK253482	OK253819	OK253972
	CSF9933 ^e,h,i^; CGMCC3.18875	ABBABB	Soil (*Eucalyptus* plantation)	Xinluo, Longyan, Fujian, China	25°07′08.597″ N, 116°44′42.257″ E	S.F. Chen, Q.L. Liu and F.F. Liu	OK253127	OK253265	OK253409	OK253483	OK253820	OK253973
	CSF9934 ^e^	ABBABB	Soil (*Eucalyptus* plantation)	Xinluo, Longyan, Fujian, China	25°07′08.597″ N, 116°44′42.257″ E	S.F. Chen, Q.L. Liu and F.F. Liu	OK253128	OK253266	OK253410	OK253484	OK253821	OK253974
*Ca. pacifica*	CSF10024 ^e^	AAAAAA	Soil (*Eucalyptus* plantation)	Yongan, Sanming, Fujian, China	25°55′10.860″ N, 117°16′39.591″ E	S.F. Chen, Q.L. Liu and F.F. Liu	OK253129	OK253267	OK253411	OK253485	OK253822	OK253975
	CSF10129 ^e^	BAAAAA	Soil (*Eucalyptus* plantation)	Minhou, Fuzhou, Fujian, China	26°15′04.285″ N, 119°02′38.917″ E	S.F. Chen, Q.L. Liu and F.F. Liu	OK253130	OK253268	OK253412	OK253486	OK253823	OK253976
	CSF10070 ^e^	CABAAA	Soil (natural forest area)	Yanping, Nanping, Fujian, China	26°42′26.672″ N, 118°07′58.317″ E	S.F. Chen, Q.L. Liu and F.F. Liu	OK253131	OK253269	OK253413	OK253487	OK253824	OK253977
	CSF10077 ^e^	CABAAA	Soil (natural forest area)	Yanping, Nanping, Fujian, China	26°42′26.672″ N, 118°07′58.317″ E	S.F. Chen, Q.L. Liu and F.F. Liu	OK253132	OK253270	OK253414	OK253488	OK253825	OK253978
	CSF10027	-AA-AA	Soil (*Eucalyptus* plantation)	Yongan, Sanming, Fujian, China	25°55′10.860″ N, 117°16′39.591″ E	S.F. Chen, Q.L. Liu and F.F. Liu	–	OK253271	OK253415	–	OK253826	OK253979
	CSF10039	-AA-AA	Soil (*Eucalyptus* plantation)	Yongan, Sanming, Fujian, China	25°55′10.860″ N, 117°16′39.591″ E	S.F. Chen, Q.L. Liu and F.F. Liu	–	OK253272	OK253416	–	OK253827	OK253980
	CSF10130	-AA-AA	Soil (*Eucalyptus* plantation)	Minhou, Fuzhou, Fujian, China	26°15′04.285″ N, 119°02′38.917″ E	S.F. Chen, Q.L. Liu and F.F. Liu	–	OK253273	OK253417	–	OK253828	OK253981
	CSF10025	----A-	Soil (*Eucalyptus* plantation)	Yongan, Sanming, Fujian, China	25°55′10.860″ N, 117°16′39.591″ E	S.F. Chen, Q.L. Liu and F.F. Liu	–	–	–	–	OK253829	–
	CSF10026	----A-	Soil (*Eucalyptus* plantation)	Yongan, Sanming, Fujian, China	25°55′10.860″ N, 117°16′39.591″ E	S.F. Chen, Q.L. Liu and F.F. Liu	–	–	–	–	OK253830	–
	CSF10028	----A-	Soil (*Eucalyptus* plantation)	Yongan, Sanming, Fujian, China	25°55′10.860″ N, 117°16′39.591″ E	S.F. Chen, Q.L. Liu and F.F. Liu	–	–	–	–	OK253831	–
	CSF10038	----A-	Soil (*Eucalyptus* plantation)	Yongan, Sanming, Fujian, China	25°55′10.860″ N, 117°16′39.591″ E	S.F. Chen, Q.L. Liu and F.F. Liu	–	–	–	–	OK253832	–
	CSF10040	----A-	Soil (*Eucalyptus* plantation)	Yongan, Sanming, Fujian, China	25°55′10.860″ N, 117°16′39.591″ E	S.F. Chen, Q.L. Liu and F.F. Liu	–	–	–	–	OK253833	–
	CSF10071	----A-	Soil (natural forest area)	Yanping, Nanping, Fujian, China	26°42′26.672″ N, 118°07′58.317″ E	S.F. Chen, Q.L. Liu and F.F. Liu	–	–	–	–	OK253834	–
	CSF10072	----A-	Soil (natural forest area)	Yanping, Nanping, Fujian, China	26°42′26.672″ N, 118°07′58.317″ E	S.F. Chen, Q.L. Liu and F.F. Liu	–	–	–	–	OK253835	–
	CSF10076	----A-	Soil (natural forest area)	Yanping, Nanping, Fujian, China	26°42′26.672″ N, 118°07′58.317″ E	S.F. Chen, Q.L. Liu and F.F. Liu	–	–	–	–	OK253836	–
	CSF10078	----A-	Soil (natural forest area)	Yanping, Nanping, Fujian, China	26°42′26.672″ N, 118°07′58.317″ E	S.F. Chen, Q.L. Liu and F.F. Liu	–	–	–	–	OK253837	–
	CSF10079	----A-	Soil (natural forest area)	Yanping, Nanping, Fujian, China	26°42′26.672″ N, 118°07′58.317″ E	S.F. Chen, Q.L. Liu and F.F. Liu	–	–	–	–	OK253838	–
*Ca. pseudoreteaudii*	CSF10059 ^e^	AAAAAA	Soil (*Eucalyptus* plantation)	Yanping, Nanping, Fujian, China	26°46′19.651″ N, 117°57′37.233″ E	S.F. Chen, Q.L. Liu and F.F. Liu	OK253133	OK253274	OK253418	OK253489	OK253839	OK253982
	CSF10060 ^e^	AAAAAA	Soil (*Eucalyptus* plantation)	Yanping, Nanping, Fujian, China	26°46′19.651″ N, 117°57′37.233″ E	S.F. Chen, Q.L. Liu and F.F. Liu	OK253134	OK253275	OK253419	OK253490	OK253840	OK253983
	CSF10058	-AA-AA	Soil (*Eucalyptus* plantation)	Yanping, Nanping, Fujian, China	26°46′19.651″ N, 117°57′37.233″ E	S.F. Chen, Q.L. Liu and F.F. Liu	–	OK253276	OK253420	–	OK253841	OK253984
	CSF10061	-AA-AA	Soil (*Eucalyptus* plantation)	Yanping, Nanping, Fujian, China	26°46′19.651″ N, 117°57′37.233″ E	S.F. Chen, Q.L. Liu and F.F. Liu	–	OK253277	OK253421	–	OK253842	OK253985
	CSF10062	-AA-AA	Soil (*Eucalyptus* plantation)	Yanping, Nanping, Fujian, China	26°46′19.651″ N, 117°57′37.233″ E	S.F. Chen, Q.L. Liu and F.F. Liu	–	OK253278	OK253422	–	OK253843	OK253986

^a^ New species described in this study are indicated in bold. ^b^ *CSF* = Culture Collection at the Research Institute of Fast-growing Trees (RIFT)/China Eucalypt Research Centre (CERC), Chinese Academy of Forestry (CAF), ZhanJiang, Guangdong Province, China; *CGMCC* = China General Microbiological Culture Collection Center, Beijing, China. ^c^ Genotype within each identified species, determined by sequences of *act*, *cmdA*, *his3*, *rpb2*, *tef1* and *tub2* regions; ‘-’ means not available. ^d^ *act* = actin; *cmdA* = calmodulin; *his3* = histone H3; *rpb2* = the second largest subunit of RNA polymerase; *tef1* = translation elongation factor 1-alpha; *tub2* = β-tubulin. ^e^ Isolates used in phylogenetic analyses. ^f^ *N/A* represents the relative locus was not successfully amplified in the current study. ^g^ ‘–’ represents the relative locus was not amplified in the current study. ^h^ Isolates used in morphological and culture growth studies. ^i^ Isolates used for mating studies. ^j^ Isolates that represent ex-type cultures are indicated in bold.

## Appendix C. Morphology of Six Previously Described *Calonectria* Species Collected in This Study

***Calonectria aconidialis***

Description: *Ascomata* perithecial, solitary or in groups of two, orange, becoming orange-brown with age; in section, apex and body orange, base red-brown, subglobose to ovoid, 368–491 μm high, 335–455 μm diam, body turning dark orange to red, and base dark red-brown in 3% KOH+; ascomatal wall rough, consisting of two thick-walled layers; outer layer of *textura globulosa*, 23–82 μm thick, cells becoming more compressed towards the inner layer of *textura angularis*, 8–21 μm thick, cells becoming thin-walled and hyaline towards the centre; outermost cells 21–35 × 7–21 μm, cells of inner layer 9–34 × 2–9 μm; ascomatal base up to 201 μm wide, consisting of dark red, angular cells, merging with an erumpent stroma; cells of the outer wall layer continuous with the pseudoparenchymatous cells of the erumpent stroma. *Asci* 8-spored, clavate, 68–143 × 10–22 μm, tapering into a long thin stalk. *Ascospores* aggregated in the upper third of the ascus, hyaline, guttulate, fusoid with rounded ends, straight to slightly curved, 1-septate, constricted at the septum, (24.5–)30.5–37.5(–42.5) × (4–)4.5–5.5(–7) μm (av. = 34 × 5 μm). *Macroconidiophores* consisting of a stipe, a suite of penicillate arranged fertile branches, a stipe extension, and a terminal vesicle; stipe septate, hyaline, smooth, 27–134 × 4–6 μm, stipe extension septate, straight to flexuous 64–129 μm long, 2–4 μm wide at the apical septum, terminating in a obpyriform to sphaeropedunculate vesicle, 3–7 μm diam; lateral stipe extensions (90° to main axis) moderate, 31–88 μm long, 1.5–3 μm wide at the apical septum, terminating in obpyriform vesicles, 2−5 μm. *Conidiogenous apparatus* 37–134 μm wide, and 41–128 μm long; primary branches aseptate, 15–27 × 3–5 μm; secondary branches aseptate, 12–20 × 3–4.5 μm; tertiary branches aseptate, 11–15 × 3–4 μm; quaternary branches aseptate, 8−16 × 3–5 μm, each terminal branch producing 2–6 phialides; phialides elongate doliiform to reniform, hyaline, aseptate, 9–18 × 2–5 μm, apex with minute periclinal thickening and inconspicuous collarette. *Macroconidia* cylindrical, rounded at both ends, straight, (40–)46–54.5(–63.5) × (3.5–)4.5–5(–6) μm (av. = 50 × 5 μm), 1-septate, lacking a visible abscission scar, held in parallel cylindrical clusters by colourless slime. Mega- and microconidia not observed.
Culture characteristics: Colonies producing abundant white to cinnamon (62) aerial mycelium at 25 °C on MEA, moderate sporulation on the medium surface; reverse sienna (8) to umber (9) after 7 d; chlamydospores extensive throughout the medium forming microsclerotia. Optimal growth temperature 25 °C, no growth at 5 °C and 35 °C, after 7 d, colonies at 10 °C, 15 °C, 20 °C, 25 °C and 30 °C reached 21.5 mm, 31.2 mm, 57.1 mm, 81.3 mm and 55.2 mm, respectively.
Specimens examined: China: Fujian Province, Longyan Region, Xinluo District (25°07′08.597″ N, 116°44′42.257″ E), from soil collected in a *Eucalyptus* plantation, 6 November 2016, S.F. Chen, Q.L. Liu and F.F. Liu (HMAS249929, culture CSF9937); Fujian Province, Longyan Region, Liancheng County (25°26′14.348″ N, 116°38′42.400″ E), from soil under a natural forest, 6 November 2016, S.F. Chen, Q.L. Liu and F.F. Liu (HMAS249930, culture CSF9957).
Notes: Isolates CSF9937, CSF9938 and CSF9957 were crossed with each other in all possible combinations on MSA to which autoclaved toothpicks had been placed, randomly distributed on the agar surface. Isolates CSF9937 and CSF9938 readily formed protoperithecia within two weeks, and perithecia with viable ascospores were produced within four weeks, when they crossed with themselves. After eight weeks of incubation, isolate CSF9957 failed to form sexual structures in any combination. *Calonectria aconidialis* is a species in the *Ca. kyotensis* species complex. The ascospores of *Ca. aconidialis* obtained in this study (av. = 34 × 5 μm) were smaller than those of the originally described *Ca. aconidialis* (av. = 36 × 6 μm) [11].

***Calonectria hongkongensis***

Description: *Ascomata* perithecial, solitary or in groups of up to three, orange, becoming red-brown with age; in section, apex and body orange, base dark red-brown, subglobose to ovoid, 243–376 μm high, 219–355 μm diam, body turning red, and base dark red-brown in 3% KOH+; ascomatal wall rough, consisting of two thick-walled layers; outer layer of *textura globulosa*, 31–54 μm thick, cells becoming more compressed towards the inner layer of *textura angularis*, 10–28 μm thick, cells becoming thin-walled and hyaline towards the centre; outermost cells 10–25 × 9–23 μm, cells of inner layer 6–24 × 2–6 μm; ascomatal base up to 168 μm wide, consisting of dark red, angular cells, merging with an erumpent stroma; cells of the outer wall layer continuous with the pseudoparenchymatous cells of the erumpent stroma. *Asci* 8-spored, clavate, 82–148 × 12–32 μm, tapering into a long thin stalk. *Ascospores* aggregated in the upper third of the ascus, hyaline, guttulate, fusoid with rounded ends, straight to slightly curved, 1-septate, constricted at the septum, (23–)25–30(–34) × (4–)5–7(–8) μm (av. = 28 × 6 μm). *Macroconidiophores* consisting of a stipe, a suite of penicillate arranged fertile branches, a stipe extension, and a terminal vesicle; stipe septate, hyaline, smooth, 47–117 × 4–8 μm, stipe extension septate, straight to flexuous 68–198 μm long, 1–4 μm wide at the apical septum, terminating in a sphaeropedunculate vesicle, 4–10 μm diam; lateral stipe extensions (90° to main axis) abundant, 42–111 μm long, 1–3 μm wide at the apical septum, terminating in obpyriform vesicles, 2−6 μm. *Conidiogenous apparatus* 37–146 μm wide, and 41–111 μm long; primary branches aseptate, 12–28 × 3–5.5 μm; secondary branches aseptate, 9.5–19 × 3–6 μm; tertiary branches aseptate, 9–13 × 3–5 μm, additional branches –5, aseptate, 8–15 × 2–4.5 μm, each terminal branch producing 2–4 phialides; phialides elongate doliiform to reniform, hyaline, aseptate, 8–14 × 2–5 μm, apex with minute periclinal thickening and inconspicuous collarette. *Macroconidia* cylindrical, rounded at both ends, straight, (34–)37–41(–44) × (3–)3.5–4(–5) μm (av. = 39 × 4 μm), 1-septate, lacking a visible abscission scar, held in parallel cylindrical clusters by colourless slime. Mega- and microconidia not observed.
Culture characteristics: Colonies forming abundant white to sienna (8) aerial mycelium at 25 °C on MEA, with irregular margins, abundant sporulation; surface rust-coloured (39); reverse sienna (8) to umber (9) after 7 d. Chlamydospores extensive throughout the medium forming microsclerotia. Optimal growth temperature 25 °C, no growth at 5 °C and 35 °C, after 7 d, colonies at 10 °C, 15 °C, 20 °C, 25 °C and 30 °C reached 21.2 mm, 26.1 mm, 46.3 mm, 69.1 mm and 64.1 mm, respectively.
Specimens examined: China: Fujian Province, Zhangzhou Region, Hua’an county (24°53′49.369″ N, 117°32′45.070″ E), from soil collected in a *Eucalyptus* plantation, 5 November 2016, S.F. Chen, Q.L. Liu and F.F. Liu (HMAS249931, culture CSF9784).
Notes: Isolates CSF7124, CSF9784 and CSF9794 were crossed with each other in all possible combinations on MSA. Isolates CSF7124 and CSF9784 readily formed protoperithecia within two weeks, and perithecia with viable ascospores were produced within four weeks, when they crossed with themselves. After eight weeks of incubation, isolate CSF9794 failed to form sexual structures in any combination. *Calonectria hongkongensis* is a species in the *Ca. kyotensis* species complex. The ascospores and macroconidia of *Ca. hongkongensis* obtained in this study (ascospores: av. = 28 × 6 μm; macroconidia: av. = 39 × 4 μm) were shorter than those of the originally described *Ca. hongkongensis* (ascospores: av. = 31 × 6 μm; macroconidia: av. = 46.5 × 4 μm) [23]. The vesicle of *Ca.* *hongkongensis* obtained in this study (4–10 μm) was narrower than those of the originally described *Ca. hongkongensis* (8–14 μm) [23].

***Calonectria ilicicola***

Description: *Ascomata* perithecial, solitary or in groups of two, orange to red, becoming red-brown with age; in section, apex and body red-brown, base dark red-brown, subglobose to ovoid, 375–509 μm high, 363–474 μm diam, body turning dark red, and base dark red-brown in 3% KOH+; ascomatal wall rough, consisting of two thick-walled layers; outer layer of *textura globulosa*, 47–75 μm thick, cells becoming more compressed towards the inner layer of *textura angularis*, 14–30 μm thick, cells becoming thin-walled and hyaline towards the centre; outermost cells 9–40 × 8–36 μm, cells of inner layer 10–23 × 2–7 μm; ascomatal base up to 208 μm wide, consisting of dark red, angular cells, merging with an erumpent stroma; cells of the outer wall layer continuous with the pseudoparenchymatous cells of the erumpent stroma. *Asci* 8-spored, clavate, 70–137 × 12–34 μm, tapering into a long thin stalk. *Ascospores* aggregated in the upper third of the ascus, hyaline, guttulate, fusoid with rounded ends, straight to slightly curved, 1-septate, not or slightly constricted at the septum, (30–)37–46.5(–58) × (4–)5–6(–8) μm (av. = 42 × 5 μm). *Macroconidiophores* consisting of a stipe, a suite of penicillate arranged fertile branches, a stipe extension, and a terminal vesicle; stipe septate, hyaline, smooth, 12–98 × 4–7 μm, stipe extension septate, straight to flexuous 111–216 μm long, 2–4.5 μm wide at the apical septum, terminating in an ovoid to sphaeropedunculate vesicle, 6–13 μm diam; lateral stipe extensions (90° to main axis) absent. *Conidiogenous apparatus* 32–94 μm wide, and 49–106 μm long; primary branches aseptate, 12–34 × 4–6 μm; secondary branches aseptate, 4–21 × 3.5–6 μm; tertiary branches aseptate, 9–17 × 4–6 μm, each terminal branch producing 2–4 phialides; phialides elongate doliiform to reniform, hyaline, aseptate, 8–15 × 3–5 μm, apex with minute periclinal thickening and inconspicuous collarette. *Macroconidia* cylindrical, rounded at both ends, straight, (58–)63–70(–76) × 6–7(–8) μm (av. = 67 × 7 μm), (1–)3-septate, lacking a visible abscission scar, held in parallel cylindrical clusters by colourless slime. Mega- and microconidia not observed.
Culture characteristics: Colonies forming abundant white to cinnamon (62) aerial mycelium at 25 °C on MEA, with irregular margins, profuse sporulation; reverse with cinnamon (62) outer margin, and rust (39) inner region after 7 d. Chlamydospores extensive throughout the medium forming microsclerotia. Optimal growth temperature 25 °C, no growth at 5 °C and 35 °C, after 7 d, colonies at 10 °C, 15 °C, 20 °C, 25 °C and 30 °C reached 16.1 mm, 24.9 mm, 54.8 mm, 74.3 mm and 66.4 mm, respectively.
Specimens examined: China: Fujian Province, Longyan Region, Zhangping County (25°17′10.882″ N, 117°27′33.635″ E), from soil collected in a *Eucalyptus* plantation, 6 November 2016, S.F. Chen, Q.L. Liu and F.F. Liu (HMAS249932, culture CSF9862).
Notes: Isolates CSF9862 and CSF9863 were crossed with each other on MSA and they were readily formed protoperithecia within two weeks, and perithecia with viable ascospores were produced within four weeks, when they crossed with themselves. *Calonectria ilicicola* is a species in the *Ca. kyotensis* species. The ascospores of *Ca. ilicicola* (av. = 42 × 5.5 μm) obtained in this study were smaller than those of the originally described *Ca. ilicicola* (av. = 45 × 6 μm) [17], and the macroconidia of *Ca. ilicicola* (av. = 67 × 7 μm) were larger than those of the originally described *Ca. ilicicola* (av. = 62 × 6 μm) [17], and they share similar vesicle dimensions.

***Calonectria kyotensis***

Description: *Ascomata* perithecial, solitary or in groups of up to four, orange, becoming red-brown with age; in section, apex and body orange, base dark red-brown, subglobose to ovoid, 322–482 μm high, 296–432 μm diam, body turning red, and base dark red-brown in 3% KOH+; ascomatal wall rough, consisting of two thick-walled layers; outer layer of *textura globulosa*, 8–24 μm thick, cells becoming more compressed towards the inner layer of *textura angularis*, 25–59 μm thick, cells becoming thin-walled and hyaline towards the centre; outermost cells 14–25 × 8–13 μm, cells of inner layer 10–30 × 2–6 μm; ascomatal base up to 234 μm wide, consisting of dark red, angular cells, merging with an erumpent stroma; cells of the outer wall layer continuous with the pseudoparenchymatous cells of the erumpent stroma. *Asci* 8-spored, clavate, 73–125 × 15–29 μm, tapering into a long thin stalk. *Ascospores* aggregated in the upper third of the ascus, hyaline, guttulate, fusoid with rounded ends, straight to slightly curved, 1(–3)-septate, constricted at the septum, (26–)31–38.5(–43.5) × (5–)5.5–7.5(–9.5) μm (av. = 34.5 × 6.5 μm). *Macroconidiophores* consisting of a stipe, a suite of penicillate arranged fertile branches, a stipe extension, and a terminal vesicle; stipe septate, hyaline, smooth, 36–135 × 4–9 μm, stipe extension septate, straight to flexuous 69.5–222 μm long, 2–4 μm wide at the apical septum, terminating in a sphaeropedunculate vesicle, 4–10 μm diam; lateral stipe extensions (90° to main axis) abundant, 41–108 μm long, 1–3 μm wide at the apical septum, terminating in sphaeropedunculate vesicles, 3−7 μm. *Conidiogenous apparatus* 40–110 μm wide, and 36–108 μm long; primary branches aseptate, 14–31 × 4–6 μm; secondary branches aseptate, 9–22 × 3–5 μm; tertiary branches aseptate, 7–16 × 3–5 μm, quaternary branches aseptate, 8−11 × 3–5 μm, each terminal branch producing 2–4 phialides; phialides doliiform to reniform, hyaline, aseptate, 6–10 × 2–4 μm, apex with minute periclinal thickening and inconspicuous collarette. *Macroconidia* cylindrical, rounded at both ends, straight, (28–)32–35.5(–39.5) × (2.5–)3–4(–4.5) μm (av. = 33.5 × 3.5 μm), 1-septate, lacking a visible abscission scar, held in parallel cylindrical clusters by colourless slime. Mega- and microconidia not observed.
Culture characteristics: Colonies forming abundant white to sienna (8) aerial mycelium at 25 °C on MEA, with feather, irregular margins, profuse sporulation; reverse sienna (8) to umber (9) after 7 d. Chlamydospores extensive throughout the medium forming microsclerotia. Optimal growth temperature 25 °C, no growth at 5 °C and 35 °C, after 7 d, colonies at 10 °C, 15 °C, 20 °C, 25 °C and 30 °C reached 16.2 mm, 23.2 mm, 52.1 mm, 66.3 mm and 61.5 mm, respectively.
Specimens examined: China: Fujian Province, Zhangzhou Region, Hua’an county (24°53′49.369″ N, 117°32′45.070″ E), from soil collected in a *Eucalyptus* plantation, 5 November 2016, S.F. Chen, Q.L. Liu and F.F. Liu (HMAS249933, culture CSF9824); Fujian Province, Longyan Region, Liancheng county (25°33′06.994″ N, 116°41′42.328″ E), from soil collected in a *Eucalyptus* plantation, 6 November 2016, S.F. Chen, Q.L. Liu and F.F. Liu (HMAS249934, culture CSF10004).
Notes: Isolates CSF7130, CSF9824 and CSF10004 were crossed with each other in all possible combinations on MSA. Isolates CSF9824 readily formed protoperithecia within two weeks. After eight weeks of incubation, isolates CSF7130 and CSF10004 failed to form sexual structures in any combination. *Calonectria kyotensis* is a species in the *Ca. kyotensis* species. The ascospores of *Ca. kyotensis* (av. = 34.5 × 6.5 μm) obtained in this study were longer than those of the originally described *Ca. kyotensis* (av. = 29 × 6 μm) [47], while the macroconidia of *Ca. kyotensis* (av. = 33.5 × 3.5 μm) in this study were shorter than those of the originally described *Ca. kyotensis* (av. = 41 × 4 μm) [47], and the vesicle in this study (4–10 μm) was narrower than those of originally described *Ca. kyotensis* (8.8–19 μm) [47].

***Calonectria pacifica***

Description: *Sexual morph* unknown. *Macroconidiophores* consisting of a stipe, a suite of penicillate arranged fertile branches, a stipe extension, and a terminal vesicle; stipe septate, hyaline, smooth, 44–115 × 4–7 µm; stipe extensions septate, straight to flexuous 73.5–171 µm long, 2–3.5 µm wide, at the apical septum, terminating in an ovoid to sphaeropedunculate vesicle, 4–10 µm diam; lateral stipe extensions (90° to main axis) abundant, 36–98 μm long, 1.5–2.5 μm wide at the apical septum, terminating in an ovoid vesicles, 3–5 µm diam. *Conidiogenous apparatus* 45–105 μm wide, and 35–81 μm long; primary branches aseptate, 12.5–23 × 4–6 μm; secondary branches aseptate, 10–20 × 3–6 μm; tertiary branches aseptate, 10–15 × 3–5 μm, each terminal branch producing 2–4 phialides; phialides doliiform to reniform, hyaline, aseptate, 6−15 × 3–5 μm, apex with minute periclinal thickening and inconspicuous collarette. *Macroconidia* cylindrical, rounded at both ends, straight, (36–)40–46(–48) × (3.5–)4–5(–6) μm, (av. = 43 × 5 μm), 1-septate, lacking a visible abscission scar, held in parallel cylindrical clusters by colourless slime. Mega- and microconidia not observed.
Culture characteristics: Colonies forming sparse white to sienna (8) aerial mycelium at 25 °C on MEA, with feathery, irregular margins at the edges, abundant sporulation; reverse sienna (8) to umber (9) after 7 d. Optimal growth temperature 25 °C, no growth at 5 °C and 35 °C, after 7 d, colonies at 10 °C, 15 °C, 20 °C, 25 °C and 30 °C reached 15.1 mm, 21.4 mm, 45.1 mm, 58.2 mm and 42.1 mm, respectively.
Specimens examined: China: Fujian Province, Nanping Region, Yanping District (26°42′26.672″ N, 118°07′58.317″ E), from soil under a natural forest, 08 November 2016, S.F. Chen, Q.L. Liu and F.F. Liu (HMAS249938, culture CSF10070); Fujian Province: Nanping Region, Yanping District (26°42′26.672″ N, 118°07′58.317″ E), from soil under a natural forest, 08 November 2016, S.F. Chen, Q.L. Liu and F.F. Liu (HMAS249939, culture CSF10077).
Notes: Isolates CSF10024, CSF10070 and CSF10077 were crossed with each other in all possible combinations on MSA and failed to form sexual structures in any combination. *Calonectria pacifica* is a species in the *Ca. kyotensis* species complex. The macroconidia of *Ca. pacifica* (av. = 43 × 5 μm) obtained in this study were shorter than those of the originally described *Ca. pacifica* (av. = 55 × 4.5 μm) [17], and the vesicles were narrower than those of originally described strains of *Ca. pacifica* (7–15 μm) [17].

***Calonectria pseudoreteaudii***

Description: *Sexual morph* unknown. *Macroconidiophores* consisting of a stipe, a suite of penicillate arranged fertile branches, a stipe extension, and a terminal vesicle; stipe septate, hyaline, smooth, 81–145 × 3–8 µm; stipe extensions septate, straight to flexuous 150–268 µm long, 5–7 µm wide, at the apical septum, terminating in a narrowly clavate vesicle, 3–5 µm diam. *Conidiogenous apparatus* 68–140 µm long, and 30–92 µm wide; primary branches aseptate or 1-septate, 19–34 × 4–6 μm; secondary branches aseptate, 16–25 × 4–5 μm; tertiary branches aseptate, 13–22 × 3–5 μm, each terminal branch producing 1–3 phialides; phialides cylindrical to allantoid, hyaline, aseptate, 10−18 × 3–5 μm, apex with minute periclinal thickening and inconspicuous collarette. *Macroconidia* cylindrical, rounded at the apex, flattened at the base, straight, (54.5–)73–88.5(–96) × (6–)6.5–8(–9) μm, (av. = 81 × 7.5 μm), 5-septate, lacking a visible abscission scar, held in parallel cylindrical clusters by colourless slime. Mega- and microconidia not observed.
Culture characteristics: Colonies forming white to sienna (8) aerial mycelium at 25 °C on MEA, with feathery, regular margins at the edges, abundant sporulation; reverse sienna (8) to chestnut (40) after 7 d; chlamydospores extensive throughout the medium, forming microsclerotia. Optimal growth temperature 25 °C, no growth at 5 °C and 35 °C, after 7 d, colonies at 10 °C, 15 °C, 20 °C, 25 °C and 30 °C reached 19.3 mm, 25.1 mm, 49.2 mm, 59.1 mm and 47.1 mm, respectively.
Specimens examined: China: Fujian Province, Nanping Region, Yanping District (26°46′19.651″ N, 117°57′37.233″ E), from soil collected in a *Eucalyptus* plantation, 08 November 2016, S.F. Chen, Q.L. Liu and F.F. Liu (HMAS249940, culture CSF10059); Fujian Province: Nanping Region, Yanping District (26°46′19.651″ N, 117°57′37.233″ E), from soil collected in a *Eucalyptus* plantation, 08 November 2016, S.F. Chen, Q.L. Liu and F.F. Liu (HMAS249941, culture CSF10060).
Notes: Isolates CSF10059 and CSF10060 were crossed with each other on MSA and failed to form sexual structures in any combination. *Calonectria pseudoreteaudii* is a species in the *Ca. reteaudii* species complex. The macroconidia of isolates obtained in this study (av. = 81 × 7.5 μm) were much shorter than those of the originally described strains of *Ca. pseudoreteaudii* (av. = 104 × 8 μm) [24].
